# Integrating protein structural dynamics and evolutionary analysis with Bio3D

**DOI:** 10.1186/s12859-014-0399-6

**Published:** 2014-12-10

**Authors:** Lars Skjærven, Xin-Qiu Yao, Guido Scarabelli, Barry J Grant

**Affiliations:** Department of Biomedicine, University of Bergen, Bergen, Norway; Structural and Computational Biology Unit, European Molecular Biology Laboratory, Heidelberg, Germany; Department of Computational Medicine and Bioinformatics, University of Michigan, Ann Arbor, Michigan USA

**Keywords:** Protein structure, Protein dynamics, Allostery, Normal mode analysis, Molecular dynamics, Principal component analysis, Evolution

## Abstract

**Background:**

Popular bioinformatics approaches for studying protein functional dynamics include comparisons of crystallographic structures, molecular dynamics simulations and normal mode analysis. However, determining how observed displacements and predicted motions from these traditionally separate analyses relate to each other, as well as to the evolution of sequence, structure and function within large protein families, remains a considerable challenge. This is in part due to the general lack of tools that integrate information of molecular structure, dynamics and evolution.

**Results:**

Here, we describe the integration of new methodologies for evolutionary sequence, structure and simulation analysis into the Bio3D package. This major update includes unique high-throughput normal mode analysis for examining and contrasting the dynamics of related proteins with non-identical sequences and structures, as well as new methods for quantifying dynamical couplings and their residue-wise dissection from correlation network analysis. These new methodologies are integrated with major biomolecular databases as well as established methods for evolutionary sequence and comparative structural analysis. New functionality for directly comparing results derived from normal modes, molecular dynamics and principal component analysis of heterogeneous experimental structure distributions is also included. We demonstrate these integrated capabilities with example applications to dihydrofolate reductase and heterotrimeric G-protein families along with a discussion of the mechanistic insight provided in each case.

**Conclusions:**

The integration of structural dynamics and evolutionary analysis in Bio3D enables researchers to go beyond a prediction of single protein dynamics to investigate dynamical features across large protein families. The Bio3D package is distributed with full source code and extensive documentation as a platform independent R package under a GPL2 license from http://thegrantlab.org/bio3d/.

**Electronic supplementary material:**

The online version of this article (doi:10.1186/s12859-014-0399-6) contains supplementary material, which is available to authorized users.

## Background

The internal motions and intrinsic dynamics of proteins have increasingly been recognized as essential for protein function and activity [1,2]. Notable examples include the dynamic rearrangements that facilitate many enzyme turnover events [3]; the force producing structural changes of motor proteins [4]; and the conformational and allosteric mechanisms that modulate protein associations in many signal transduction cascades [5,6]. Dissecting these functional motions typically relies on the accumulation and comparison of multiple high-resolution structures for a given protein. The rapidly increasing availability of such data is precipitating the need for new approaches that integrate knowledge of molecular structure, dynamics and evolution in functional analysis. In addition to multiple structure comparisons, computational methods including molecular dynamics (MD) and normal mode analysis (NMA) have emerged as popular approaches for characterizing protein dynamics and flexibility [7-9]. However, the general lack of tools that integrate these traditionally separate analyses with methods for sequence and structural analysis represents a practical bottleneck for the systematic study of the evolution of functional motions in large protein families and superfamilies.

Current software solutions lack much of the flexibility needed for comparative studies of large heterogeneous structural datasets. For example, popular web servers for NMA typically operate on single structures and do not permit high-throughput calculations [10-12]. Software libraries such as the Molecular Modeling ToolKit (MMTK) [13] and the packages ProDy [14] and MAVEN [15] provide more advanced calculation options but generally lack direct functionality for the quantitative comparison of dynamic features of non-identical structures and sequences. These limitations complicate the assessment of functional motions in an evolutionary context. The Bio3D package [16] now provides these essential components thus greatly facilitating the study of evolutionarily related ensembles and their functional dynamics. Here, using selected case studies, we demonstrate the integration of versatile new ensemble NMA approaches and correlation network analysis facilities with enhanced interactive tools for extracting mechanistic information from molecular sequences, crystallographic structural ensembles and MD trajectories. This major update to the Bio3D package includes extensive functionality to analyze and visualize protein dynamics from both experiment and simulation, together with tools for systematic retrieval and analysis of publicly available sequence and structural data.

### Package overview and architecture

Bio3D version 2.0 now provides extensive functionality for high-throughput NMA of an ensemble of protein structures facilitating the study of evolutionary and comparative protein dynamics across protein families. The NMA module couples to major protein structure and sequence databases (PDB, PFAM, UniProt and NR) and associated search tools (including BLAST [17] and HMMER [18]). This enables the automated identification and analysis of related protein structures. Efficient elastic network model (ENM) NMA is implemented with multicore functionality to enable rapid calculation of modes even for large structural ensembles. Results of the *ensemble NMA* algorithm include *aligned* eigenvectors and mode fluctuations for the different structures in the ensemble. These can readily be analyzed and compared with a variety of implemented methodologies. This facilitates the prediction and identification of distinct patterns of flexibility among protein families or between different conformational states of the same protein. The user can perform ensemble NMA by providing a set of either PDB structures or RCSB PDB codes. Alternatively a single protein sequence or structure can be used to search the PDB for similar structures to analyze.

**Figure 1:**
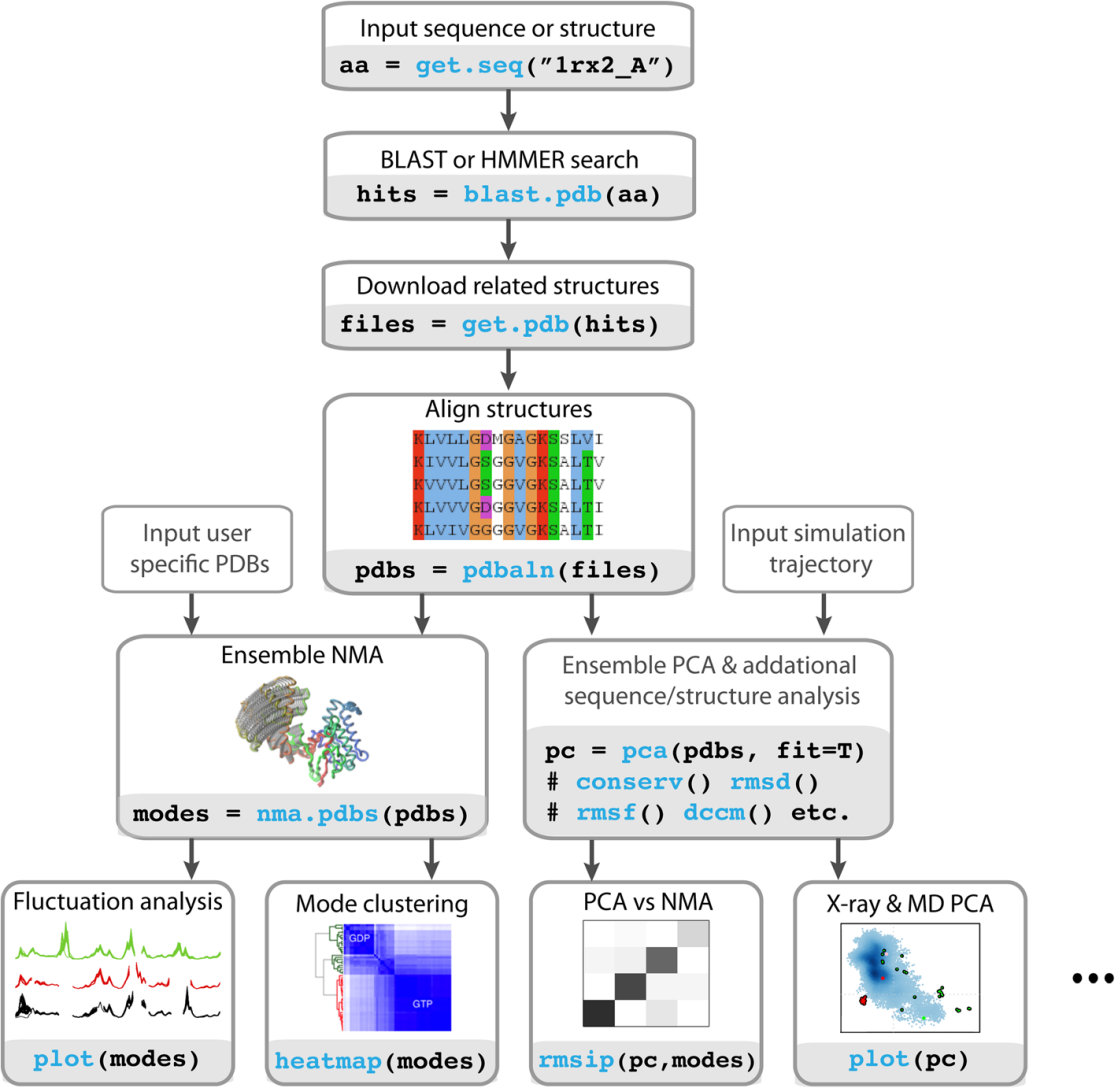
**Example workflow for** ***ensemble*** **NMA and PCA.** In this example the user starts with a single protein identifier, performs a BLAST search to identify related structures, fetches and aligns the identified structures, performs PCA and calculates the normal modes for each structure to obtain *aligned* normal mode vectors. Result interpretation and comparison of mode subsets is made available through various methods for similarity assessment.

A typical user workflow for the comparison of cross-species protein flexibility is depicted in Figure 1. In this example, we begin by fetching the protein sequence of a PDB structure with the **get.seq**() function. This sequence is then used in a BLAST or HMMER search of the full PDB database to identify related protein structures (functions **blast**() or **hmmer**()). Identified structures can then optionally be downloaded (with the function **get.pdb**()) and aligned using the function **pdbaln**(). The output will be a multiple sequence alignment together with aligned coordinate data and associated attributes. Ensemble NMA on all aligned structures can then be carried out with function **nma**(). The function provides an *“eNMA”* object containing *aligned* eigenvectors, mode fluctuations, and all pair-wise root mean squared inner product (RMSIP) values. These results are formatted to facilitate direct comparison of the flexibility patterns between protein structures, as well as clustering based on the pair-wise modes similarity. Also shown in Figure 1 is the typical application of principal component analysis (PCA) on the same experimental structures using the function **pca**(). This provides principal components of the same dimensions as the normal modes facilitating direct comparison of mode fluctuations, or alternatively mode vectors using functions such as **rmsip**() and **overlap**(). Indeed extensive new functions for the analysis of normal modes and principal components are now provided. These include cross-correlation, fluctuations, overlap, vector field, dynamic sub-domain clustering, correlation network analysis and movie generation along with integrated functions for plotting and visualization. Extensive multicore support is also included for a number of commonly used functions. This enables a significant speed-up for time-consuming tasks, such as ensemble NMA for large protein families, modes comparison, domain assignment, correlation analysis for multiple structures, and analysis for long-timescale MD simulations. Comprehensive tutorials integrating NMA with PCA, simulation data from MD, and additional sequence and structure analysis methods, including correlation network analysis, are available in Additional files 1, 2, 3 and 4.

## Implementation

### Elastic network models

A unique collection of multiple ENM force fields is now provided within Bio3D. These include the popular anisotropic network model (ANM) [19], the associated parameter-free ANM [20], and a more sophisticated C-alpha force field derived from fitting to the Amber94 all-atom potential [21]. Also included is the REACH force field employing force constants derived from MD simulations [22], and a recent parameterization providing sequence-specific force constants obtained from an ensemble of 1500 NMR structures [23]. A convenient interface for the application of user-defined force fields is also provided enabling customized normal mode calculations, perturbation analysis, and other more advanced options as detailed online and in Additional file 1.

All implemented ENMs considered here employ a harmonic potential, where the potential energy between residues *i* and *j* is given by:

$$ {U}_{ij}\left(\mathbf{r}\right)=k\left(\left\Vert {\mathbf{r}}_{\boldsymbol{ij}}^0\right\Vert \right){\left(\left\Vert {\mathbf{r}}_{\boldsymbol{ij}}\right\Vert -\left\Vert {\boldsymbol{r}}_{\boldsymbol{ij}}^0\right\Vert \right)}^2 $$

where **r** is the current protein conformation, **r**^**0**^ represents the equilibrium conformation, and ‖**r**_***ij***_‖ the distance between residues *i* and *j* [24,25]. By default, the Bio3D package employs the C-alpha force field [21] derived from fitting to the Amber94 all-atom potential with pair force constants given by

$$ k(r)=\left\{\begin{array}{c}\hfill 8.6\cdot {10}^2\cdot r-2.39\cdot {10}^3,\kern0.24em for\;r<4.0\mathring{\mathrm{A}} \hfill \\ {}\hfill 128\cdot {10}^4\cdot {r}^{-6},\;for\;r\ge 4.0\mathring{\mathrm{A}} \hfill \end{array}\right. $$

with units of *k(r)* given in kJ mol^− 1^ Å^− 2^. The selection of different force fields is described in detail both online and in Additional file 1.

### Ensemble NMA

Integrated multiple sequence and structural alignment methods are utilized to facilitate the analysis of structures of unequal composition and length. From these alignments, equivalent atom positions across structure ensembles are identified and normal mode vectors determined by calculating the effective force-constant Hessian matrix $\widehat{\mathbf{K}}$ as

$$ \widehat{\mathbf{K}}={\mathbf{K}}_{\mathbf{AA}}-{\mathbf{K}}_{\mathbf{AQ}}{\boldsymbol{K}}_{\boldsymbol{QQ}}^{-1}{\mathbf{K}}_{\mathbf{QA}} $$

where **K**_*AA*_ represents the sub-matrix of **K** corresponding to the aligned C-alpha atoms, **K**_*QQ*_ for the gapped regions, and **K**_*AQ*_ and **K**_*QA*_ are the sub-matrices relating the aligned and gapped sites [21,26]. The normal modes of the individual structure in the ensemble can then be obtained by solving the eigenvalue problem

$$ {\mathbf{V}}^T\widehat{\mathbf{K}}\mathbf{V}=\uplambda $$

where **V** is the matrix of eigenvectors and λ the associated eigenvalues.

### Ensemble PCA

Principal component analysis can be performed on any structure dataset of equal or unequal sequence composition and length to capture and characterize inter-conformer relationships. The application of PCA to both distributions of experimental structures and MD trajectories, along with its ability to provide considerable insight into the nature of conformational differences in a range of protein families has been previously discussed [27-30]. Briefly, PCA is based on the diagonalization of the covariance matrix, *C*, with elements *C*_*ij*_ calculated from the aligned and superimposed Cartesian coordinates, *r*, of equivalent Cα atoms:

$$ {C}_{ij}=\left\langle \left({r}_i-\left\langle {r}_i\right\rangle \right)\cdot \left({r}_j-\left\langle {r}_j\right\rangle \right)\right\rangle $$

where *i* and *j* enumerate all 3 *N* Cartesian coordinates (*N* is the number of atoms), and 〈*r*〉 denotes the ensemble average. Projection of the distribution onto the subspace defined by the PCs with the largest eigenvalues provides a low-dimensional representation of the structures facilitating inter-conformer analysis.

### Similarity measures

Multiple similarity measures have been implemented to provide an enhanced framework for the assessment and comparison of ensemble NMA and PCA. These measures also facilitate clustering of proteins based on their predicted modes of motion:

**Root mean square inner product** (RMSIP) measures the cumulative overlap between all pairs of the *l* largest eigenvectors [31], and is defined as:

$$ RMSIP={\left(\frac{1}{l}{\displaystyle \sum_{i=1}^l}{\displaystyle \sum_{j=1}^l}{\left({\mathbf{v}}_i^{\mathrm{A}}\cdot {\mathbf{v}}_j^{\mathrm{B}}\right)}^2\right)}^{\frac{1}{2}} $$

where ${\mathbf{v}}_i^{\mathrm{A}}$ and ${\mathbf{v}}_j^{\mathrm{B}}$ represent the *i*th and *j*th eigenvectors obtained from protein A and B, respectively. *l* is the number of modes to consider which is commonly chosen to be 10. The RMSIP measure varies between 0 (orthogonal) and 1 (identical directionality).

**Covariance overlap** provides a measure of the correspondence between the eigenvectors (**v**_*i*_) similar to the RMSIP measure, but also includes weighing by their associated eigenvalues (λ_*i*_) [32]. It ranges from 0 (orthogonal) to 1 (identical), and is defined as:

$$ CO=1-{\left(\frac{{\displaystyle {\sum}_{i=1}^l}\left({\lambda}_i^A+{\lambda}_i^B\right)-2{\displaystyle {\sum}_{i=1}^l}{\displaystyle {\sum}_{j=1}^l}\sqrt{\lambda_i^A{\lambda}_j^B}{\left({\mathbf{v}}_i^A\cdot {\mathbf{v}}_j^B\right)}^2}{{\displaystyle {\sum}_{i=1}^l}\left({\lambda}_i^A+{\lambda}_i^B\right)}\right)}^{1/2} $$

**Bhattacharyya coefficient** provides a means to compare two covariance matrices derived from NMA or an ensemble of conformers (e.g. simulation or X-ray conformers). For ENM normal modes the covariance matrix (**C**) can be calculated as the pseudo inverse of the mode eigenvectors:

$$ \mathbf{C}={\displaystyle \sum_{i=1}^{3N-6}}\frac{1}{\lambda_i}{\mathbf{v}}_i{\mathbf{v}}_i^T $$

where **v**_*i*_ represents the *i*th eigenvector, λ_*i*_ the corresponding eigenvalue, and *N* the number C-alpha atoms in the protein structure (*3 N-6* non-trivial modes). As formulated by Fuglebakk *et al*. [26,33], the Bhattacharyya coefficient can then be written as

$$ BC= exp\left[-\frac{1}{2q} \ln \left(\frac{\left|\Lambda \right|}{{\left(\left|{\mathbf{Q}}^T{\mathbf{C}}_A\mathbf{Q}\right|\left|{\mathbf{Q}}^T{\mathbf{C}}_{\mathrm{B}}\mathbf{Q}\right|\right)}^{1/2}}\right)\right] $$

where **Q** is the matrix of the principal components of (**C**_A_ + **C**_B_)/2, Λ is diagonal matrix containing the corresponding eigenvalues, and *q* the number of modes needed to capture 90% of the variance of **Q**. The Bhattacharyya coefficient varies between 0 and 1, and equals to 1 if the covariance matrices (**C**_A_ and **C**_B_) are identical.

**Squared Inner Product** (SIP) measures the linear correlation between two atomic fluctuation profiles [33,34]. It varies between 0 and 1 and is defined as

$$ SIP=\frac{{\left({\boldsymbol{w}}_{\boldsymbol{A}}^{\boldsymbol{T}}{\boldsymbol{w}}_{\boldsymbol{B}}\right)}^2}{\left({\boldsymbol{w}}_{\boldsymbol{A}}^{\boldsymbol{T}}{\boldsymbol{w}}_{\boldsymbol{A}}\right)\left({\boldsymbol{w}}_{\boldsymbol{B}}^{\boldsymbol{T}}{\boldsymbol{w}}_{\boldsymbol{B}}\right)} $$

where ***w***_***A***_ and *w*_*B*_***w***_***B***_ are vectors of length *N* containing the fluctuation value (e.g. RMSF) for each atom in protein A and B, respectively.

### PCA of cross-correlation and covariance matrices

New functionality facilitates PCA of residue-residue cross-correlations and covariance matrices derived from ensemble NMA. This analysis can be formulated as

$$ {\mathbf{B}}^T\mathbf{Y} \mathbf{B}=\Gamma $$

where **Υ** is a matrix containing the elements of the *M* correlation/covariance matrices (with one row per structure), **B** the eigenvectors and Γ the associated eigenvalues. Projection into the sub-space defined by the largest eigenvectors enables clustering of the structures based on the largest variance within the cross-correlation or covariance matrices.

All similarity measures described above can be utilized for clustering the ensemble of structures based on their normal modes. Various clustering algorithms are available, such as k-means clustering, as well as hierarchical clustering using the Ward’s minimum variance method, or single, complete and average linkage. The application and comparison of the described similarity measures is presented in Additional file 2.

### Force constants variance weighting

We propose to incorporate knowledge on the accessible conformational ensemble (e.g. all available X-ray structures) to lift the dependency of the force constants on the single structure they were derived from. We weigh the force constants with the variance of the pairwise residue distances derived from the ensemble of structures. The weights (W_*ij*_) and the modified force constants (*k’*_*ij*_(*r*)) can then be calculated as

$$ \begin{array}{c}\hfill {W}_{ij}={\left(1-\frac{S_{ij\ }}{\widehat{s}}\right)}^{\varphi}\hfill \\ {}\hfill {k}_{ij}^{\hbox{'}}(r)={W}_{ij}\cdot {k}_{ij}(r)\hfill \end{array} $$

where *S*_*ij*_ (the elements of matrix **S**) represents the variance of the distance between residues *i* and *j* in the ensemble, *ŝ* is the maximum of such variance for any pair of atoms, and *φ* is an optional scaling factor. The application of force constant weighting is presented in Additional file 1.

### Identification of dynamic domains

Analysis and identification of dynamic domains, *i.e.* parts of the molecule that move as relatively rigid entities within a conformational ensemble, is made available through a new implementation of the GeoStaS algorithm previously presented as a standalone Java program [35]. The algorithm relies on the identification of the best pairwise superimposition of atomic trajectories based on rotation and translation in quaternion space. The resulting *atomic movement similarity matrix* provides a means for clustering the atoms in the system based on their respective similarity. This approach has the advantage of capturing the potential correlation in rotational motions of two atoms placed on opposite sites, which may otherwise be found to be anti-correlated in a standard cross-correlation analysis. The application of GeoStaS is demonstrated in Additional files 1 and 2 for both single structure and ensemble NMA, as well as for ensembles of PDB structures and MD trajectories.

### Correlation network analysis

Correlation network analysis can be employed to identify protein segments with correlated motions. In this approach, a weighted graph is constructed where each residue represents a node and the weight of the connection between nodes, *i* and *j*, represents their respective cross-correlation value, *c*_*ij*_, expressed by either the Pearson-like form [36], or the linear mutual information [37]. Here we propose an approach related to that introduced by Sethi *et al*. [38], but using multiple correlation matrices derived from the input ensemble instead of contact maps. Specifically, the correlation matrix (**C**) is calculated for each structure in the ensemble NMA. Then, edges are added for residue pairs with *c*_*ij*_ ≥ *c*_0_ across all experimental structures, where *c*_0_ is a constant. In addition, edges are added for residues where *c*_*ij*_ ≥ *c*_0_ for at least one of the structures and the Cα-Cα distance, *d*_*ij*_, satisfies *d*_*ij*_ ≤ 10 Å for at least 75% of all conformations. Edges weights are then calculated with − *log*(〈*c*_*ij*_〉), where 〈 ⋅ 〉 denotes the ensemble average. Girvan and Newman betweeness clustering [39] is then performed to generate aggregate nodal clusters, or communities, that are highly intra-connected but loosely inter-connected. Visualization of the resulting network and community structures in both 2D and 3D along with additional clustering and analysis options are also provided. See Additional file 4 for a complete example of the integration of ensemble NMA with correlation network analysis.

## Results and discussion

In this section we demonstrate the application of new Bio3D functionality for analyzing functional motions in two distinct protein systems. Further examples, along with executable code, are provided in Additional files 1, 2, 3 and 4.

### Cross-species analysis of DHFR

Dihydrofolate reductase (DHFR) plays a critical role in promoting cell growth and proliferation in all organisms by catalyzing the reaction of dihydrofolate to tetrahydrofolate, an essential precursor for thymidylate synthesis [40]. DHFR is a major target for several antibiotics and has been subject of extensive structural studies. There are currently more than 500 DHFR structures in the PDB including a multitude of liganded states from a number of species. Here we demonstrate the use of Bio3D to take full advantage of this large structural data set when performing NMA. We first focus on the *E. coli*. DHFR structures before proceeding to a cross species analysis of all available DHFR structures.

**Figure 2:**
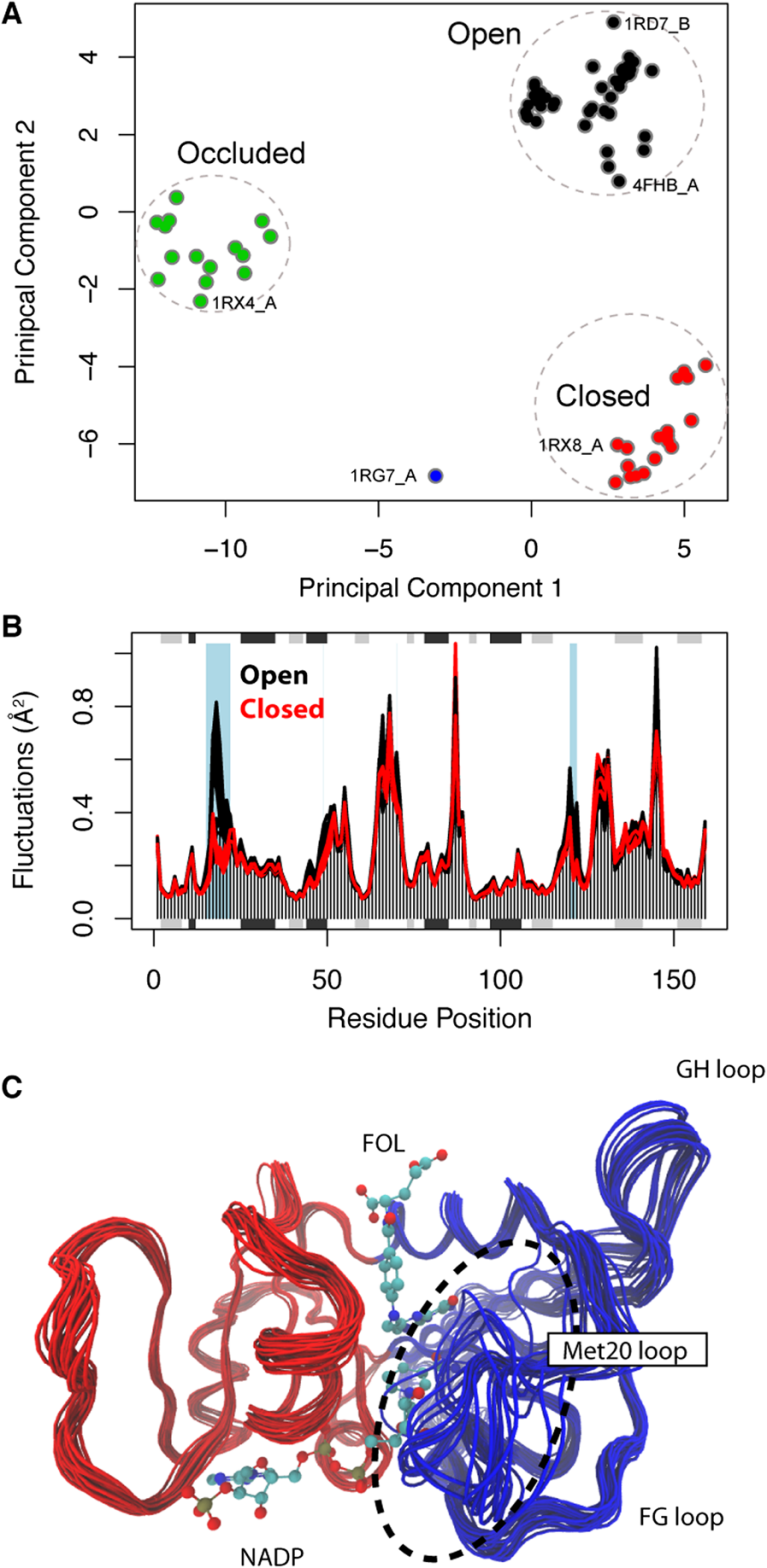
**Results of ensemble PCA and NMA on** ***E. coli*** **DHFR. (A)** Available PDB structures projected onto their first two principal components accounting for a total of 59% of the total variance. **(B)** Comparison of mode fluctuations calculated for open (black) and closed (red) conformations. The figure is generated by automated functions for plotting and the identification of areas of significant differences in residue fluctuations between groups of conformers (light blue boxes). The locations of major secondary structure elements are shown in the plot margins with β strands in gray and α helices in black. **(C)** Conformational ensemble obtained from interpolating along the first five modes of all collected *E. coli* structures. Domain analysis on the generated ensemble reveals the identification of two dynamic sub-domains colored red and blue, respectively. See Additional file 2 for full details and corresponding code for this analysis.

Following the workflow described in Figure 1 (see the *Package overview and architecture* section), we collected all 90 *E. coli*. DHFR structures from the PDB, performed a PCA to investigate the major conformational variation, and calculated the normal modes of each structure to probe for potential differences in structural flexibility. The PCA reveals that the ensemble can be divided into three major groups along their first two principal components (which collectively account for 59% of the total coordinate mean square displacements, Figure 2A). These conformers display either a closed, occluded, or an open conformation of two active site loops (termed the Met20 loop: residues 9-24, and the F-G loop: residues 116-132). NMA reveals that structures obtaining an open conformation show enhanced flexibility for the Met20 loop as compared to both the closed and occluded conformations (Figure 2B). Conversely, the F-G loop shows lower fluctuation values for the open conformation as compared to the occluded state (Additional File 2). These differences in mode fluctuations highlight the importance of considering multiple conformers in NMA, which is greatly facilitated by the Bio3D package. Additional, domain analysis with the function **geostas**() reveals the presence of two dynamic sub-domains corresponding to the adenosine-binding sub-domain and the loop sub-domain, respectively (Figure 2C). These domains are divided by a hinge region corresponding to residues Thr35 and Gln108, in agreement with previous studies [41]. This example demonstrates how integrating PCA, NMA and dynamic domain analysis on *E. coli*. DHFR structures can provide mechanistic insight into protein dynamics of functional relevance.

**Figure 3:**
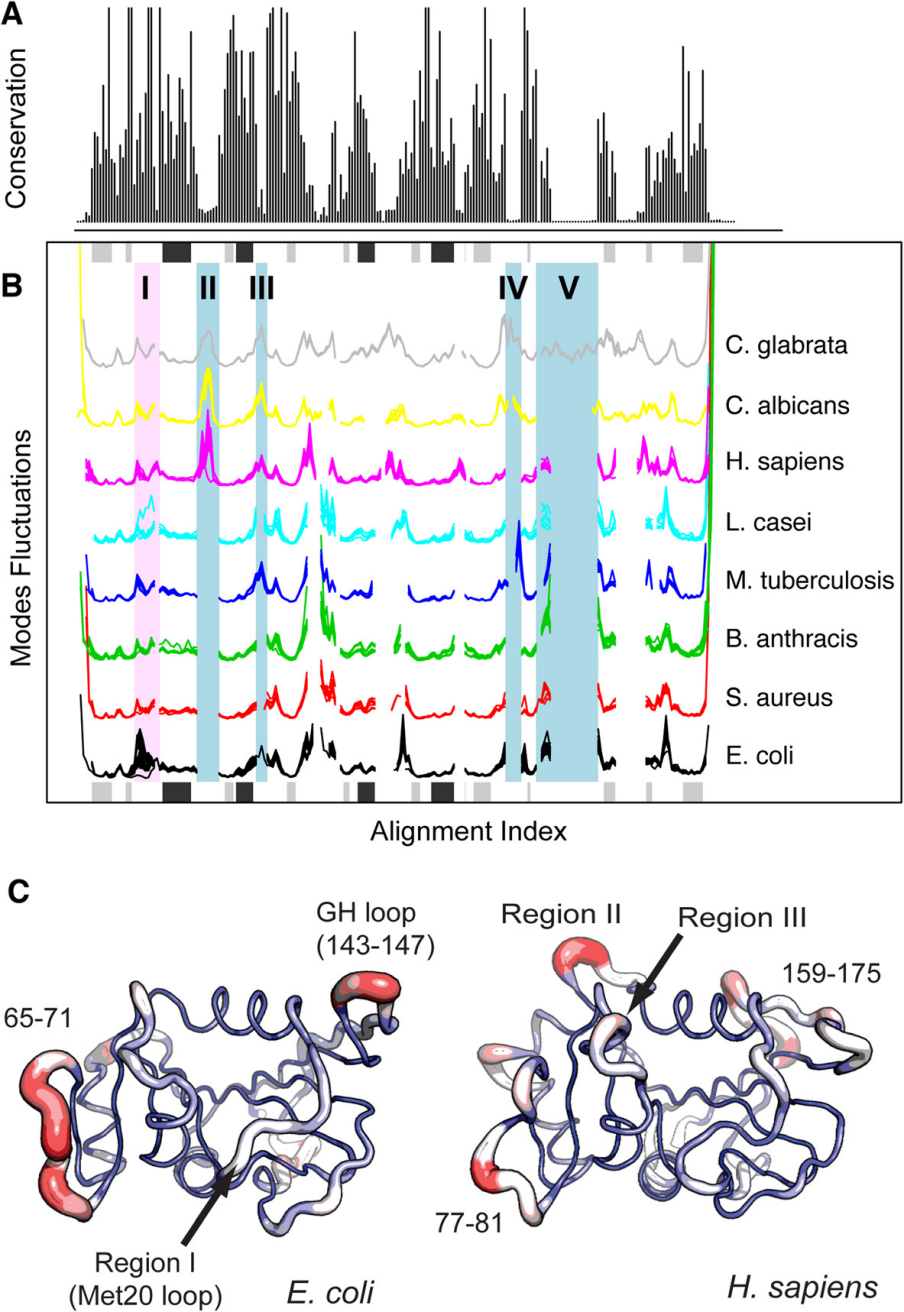
**Cross-species normal modes analysis of DHFR. (A)** Sequence conservation of the collected DHFR species. **(B)** Aligned fluctuation profiles for selected species of DHFR. Shaded blue regions depict areas discussed in the text showing different fluctuation patterns between specific species. The region shaded in light red depict the Met20 loop in *E. coli* DHFR and the corresponding loop in the remaining species. The location of major secondary structure elements in *E. coli* DHFR are also shown in the plot margins with β strands in gray and α helices in black. **(C)** A visual comparison of mode fluctuations between DHFR from *E. coli* and *H. sapiens*. Fluctuation magnitude is represented by thin to thick tube colored blue (low fluctuations), white (moderate fluctuations) to red (large fluctuations). See Additional file 3 for full details and corresponding code for this analysis.

Beginning with the knowledge of only one DHFR PDB code, the complete PCA and NMA of the *E. coli*. DHFR ensemble can be performed with only a few lines of code:
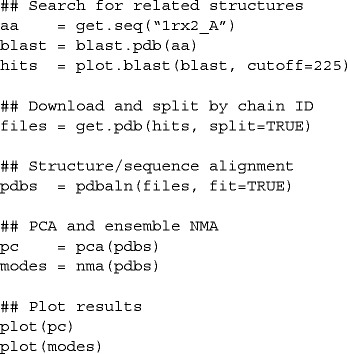


To detect more distantly related DHFR homologues we built a hidden Markov model (HMM) from the PFAM multiple sequence alignment using the Bio3D interface to PFAM and HMMER (see the *Package overview and architecture* section). The resulting HMM was used in a new search of the PDB that identified a total of 33 species from bacteria, archaea, and eukaryotes, showing a pairwise sequence identity down to 21%. NMA was carried out on 197 of these structures. The resulting fluctuation profiles are plotted for each species along with the sequence conservation in Figure 3A-B. The plot reveals an overall similar trend of residue fluctuations between the species despite their low sequence identity. While the functionally important Met20 loop display a conserved flexibility trend for most of the species, the *E. coli* structures have enhanced fluctuations in this region (region I, Figure 3). This has previously been attributed to distinct functional mechanism for ligand flux: while *E. coli* DHFR relies on loop flexibility for the opening of the active site, *H. sapiens* DHFR accomplishes this by subtle subdomain rotational hinge motions [41]. Other important differences include enhanced loop fluctuations in *H. sapiens* DHFR, which are not evident in the bacterial species (residues 43-50 and 126-131 for human DHFR; Figure 3). These fluctuations have been suggested to be important for facilitating the hinge motions in *H. sapiens* DHFR [41]. Interestingly, the flexibility pattern of the human DHFR 43-50 loop is shared with two fungal variants: *C. albicans* and *C. glabrata* (region II, Figure 3). A similar trend is apparent for residues 62-64 in human DHFR. This flexible loop is also shared with the bacterial *M. tubercolosi* species (region III), but is missing in the four other bacterial species. Finally, the two fungal species display an additional and flexible surface loop (residues 139-150 in *C. albicans* DHFR; region IV), while *C. glabrata* contains residues 164-178 specific for this species (region V). This example demonstrates how Bio3D version 2.0 can facilitate the investigation of common and divergent protein structural dynamics in large protein superfamilies.

### Heterotrimeric G-proteins

Applying ensemble NMA to heterotrimeric G-protein α-subunits (Gα) reveals nucleotide dependent structural dynamic features of functional relevance. Gα undergoes cycles of nucleotide-dependent conformational rearrangements to couple cell surface receptors to downstream effectors and signaling cascades that control diverse cellular processes. These process range from movement and division to differentiation and neuronal activity. Interaction with activated receptor promotes the exchange of GDP for GTP on Gα and its separation from its βγ subunit partners (Gβγ). Both isolated Gα and Gβγ can then interact and activate downstream effectors. GTP hydrolysis deactivates Gα, which re-associates with Gβγ effectively completing the cycle.

**Figure 4:**
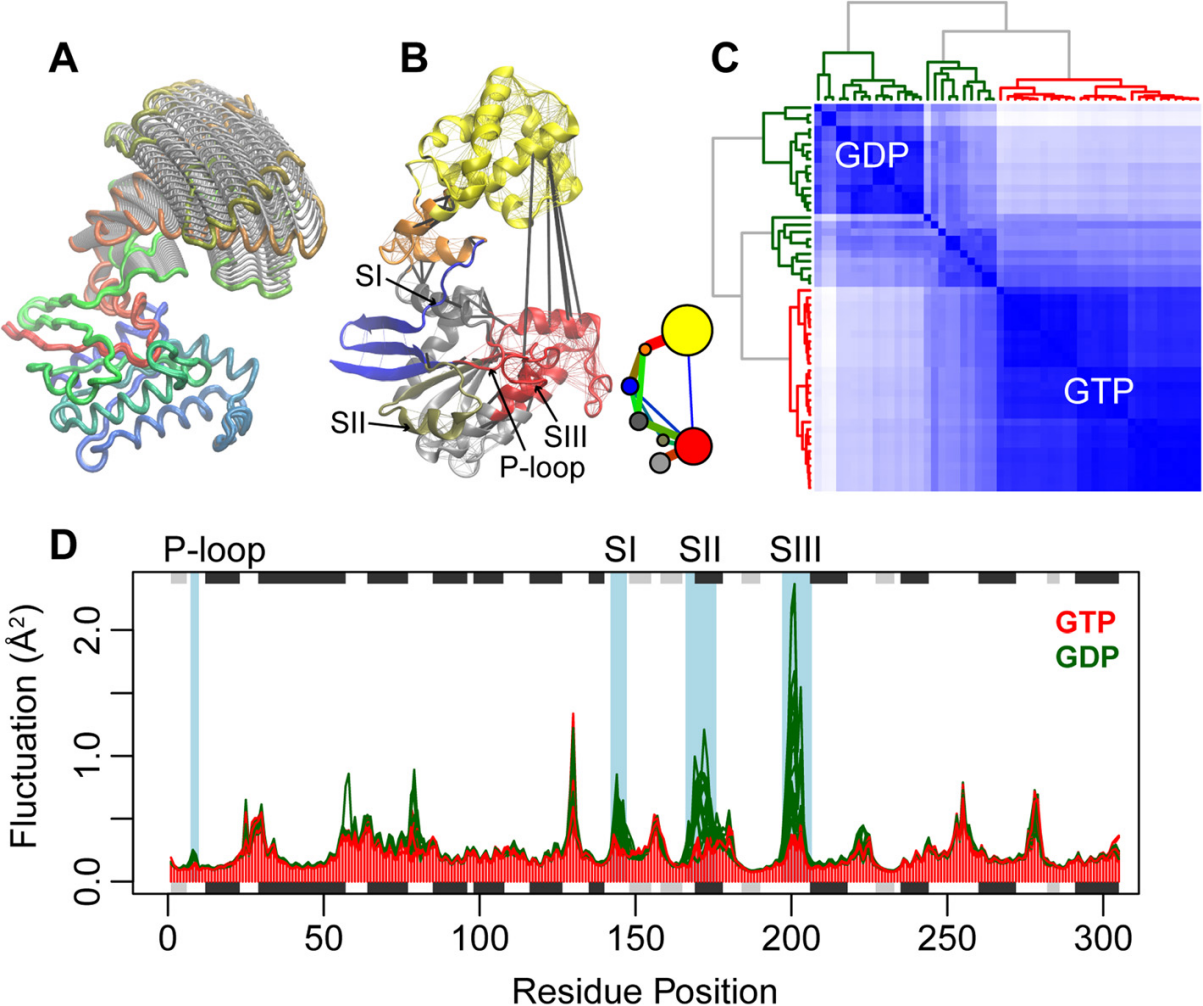
**Investigating functional dynamics in heterotrimeric G-proteins. (A)** Prediction of large-scale opening motions. **(B)** Prediction of dynamically coupled sub-domains (colored regions) from correlation network analysis of NMA results. Inter-subdomain couplings are highlighted with thick black lines. **(C)** Characterization of distinct GTP-active and GDP-inactive states from a clustering of NMA RMSIP results. **(D)** Fluctuation analysis reveals structural regions with significantly distinct flexibilities (highlighted with a blue shaded background are sites with a p-value < 0.005) between the active (red) and inactive (green) states. Full details for the reproduction of this analysis along with PCA that distinguishes GDP and GTP states can be found in the Additional file 1.

In the current application, we collected 53 PDB structures of Gα (from application of the **blast.pdb**() function). These structures were aligned with the function **pdbaln**() and their modes of motion calculated with **nma**() (Figure 1 and Additional file 1). Results from RMSIP, fluctuation, and correlation analysis indicate that the structural dynamics are nucleotide state dependent (Figure 4). The modes of motion clearly distinguish the GTP (active) and GDP (inactive) states (Figure 4C). Predicted residue fluctuations reveal areas of conserved dynamics interspersed with areas of significantly distinct flexibilities in the active and inactive states (Figure 4D). Specifically, the P-loop and switch I, switch II and switch III regions are predicted to be significantly more flexible in the GDP than in GTP state. These results are consistent with our previous structural and MD simulation studies, in which these regions were found to be strongly coupled only in the active GTP state [42]. The stabilized P-loop and switch regions are thus a potential prerequisite for GTP hydrolysis and the binding of effectors.

It has been suggested that the activation mechanism of Gα involves a large domain opening that facilitates GDP/GTP exchange [43,44]. Applying NMA to a predicted open form of Gα [42], highlights the large flexibility of the helical domain and captures this opening closing motion (Figure 4A). Combining NMA results with correlation network analysis methods, as implemented in the **cna**() function, reveals dynamically coupled subdomains that may facilitate the allosteric coupling of receptor and nucleotide binding sites (Figure 4B and Additional file 4). In summary, this example demonstrates the potential of ensemble NMA for characterizing key structural dynamic mechanisms in G proteins and other biomolecular systems.

### Related solutions and future developments

As noted in the introduction, a number of previously implemented software solutions (including multiple web-servers [10-12,45] and standalone software packages [13-15,46]) offer single structure NMA or MD analysis. These however typically lack extensive coupling to different biomolecular databases and methods for evolutionary and comparative analysis of large sequence and structural datasets (see Table 1). This lack of integrated functionality impedes efficient exploratory analysis of sequence, structure, dynamics relationships. Bio3D version 2.0 now integrates functionality for searching and fetching data from major sequence/structure databases, sequence/structure alignment and conservation analysis, high-throughput ensemble NMA and PCA of heterogeneous structures, protein structure network analysis and many commonly used functions for simulation analysis. The package also includes specifically tailored plotting and visualization functionality as well as coupling to the well-developed R environment for statistical computing and graphics. Bio3D thus offers unparalleled capabilities for both exploratory interactive and large-scale batch analysis of structural dynamic mechanisms in biomolecular systems.

**Table 1:** **Related software for analysis of protein structural dynamics**

	**MMTK 2.7**	**ProDy 1.5**	**MAVEN 1.2**	**WebNM@ 2.0**	**Bio3D 2.0**
**Dependencies**	Python, NumPy, ScientificPython	Python, NumPy, MatplotLib	Matlab Component Runtime (MCR)	Web browser	R, Muscle
**Reading and analysis of molecular sequences**	No	Yes	No	No	Yes
**Reading and analysis of multiple molecular structures**	No	Yes	Yes	Yes	Yes
**Reading and analysis of binary MD simulation trajectories**	Yes	Yes	No	No	Yes
**Biomolecular database integration**	No	PDB, PFAM^a^	No^b^	No^b^	PDB, PFAM, UNIPROT, NR^c^
**Energy minimization and MD**	Yes	No	No	No	No
**Standard NMA**	Yes	Yes	Yes	Yes	Yes
**Ensemble NMA across heterogeneous structures**	No	No	No	Yes	Yes
**Forcefields for NMA**	C-alpha, ANM, Amber all-atom	GNM/ANM, Custom	GNM/ANM, pANM, STM, nnANM, mcgANM, Custom^d^	C-alpha	C-alpha, ANM, pfANM sdENM, REACH, Custom
**Ensemble PCA across heterogeneous structures**	No	Yes	Identical structures only	No	Yes
**Correlation network analysis from NMA and MD**	No	No	No	No	Yes
**Dynamic domain analysis**	No	No	No	No	Yes
**Sequence alignment**	No	No	No	No	Yes
**Structure alignment**	Yes	Yes	No	No	Yes
**Advanced statistical analysis**	No	No	No	No	Yes
**Permits both interactive and batch analysis**	Yes	Yes	No	Yes	Yes
**Open source code available**	Yes	Yes	Yes^e^	No	Yes
**Multicore compatibility**	Yes	No	No	No	Yes
**GUI**	No	No^f^	Yes	Webserver	No^g^

^a^Read and search functionality.

^b^Read-only functionality from the PDB.

^c^Read, search, and annotation functionality, including enhanced search capabilities across multiple databases.

^d^STM: Spring Tensor Model; pANM: power ANM; nnANM: nearest neighbor ANM; mcgANM: mixed coarse graining ANM.

^e^Dependences are not open source.

^f^VMD plugin NMWiz available for single molecule NMA.

^g^Web interface for ensemble PCA and NMA in development.

Current and future development of Bio3D (see: https://bitbucket.org/Grantlab/bio3d) includes implementation of additional 3D visualization functionality, enhanced compatibility with the AMBER package [47], and further parallelization and optimization of structural alignment methods using graphical processing units (GPUs). We also plan to develop a web-interface and API for ensemble NMA and PCA to make this functionality more widely accessible. Finally, we envisage the development of new tools for structural dynamic mapping of clinical variants from next generation sequencing data and integration with the Bioconductor project [48] and tools for analysis of various omics data.

## Conclusion

Bio3D version 2.0 provides a versatile integrated environment for protein structural and evolutionary analysis with unique capabilities including high-throughput ensemble NMA for examining the dynamics of evolutionary related protein structures; a convenient interface for accessing multiple ENM force fields; and a direct integration with a large number of functions for sequence, structure and simulation analysis. The package is implemented in the R environment and thus couples to extensive graphical and statistical capabilities along with a powerful user-friendly interactive programming environment that, together with Bio3D, enables both exploratory structural bioinformatics analysis and automated batch analysis of large datasets.

## Availability and requirements

- Project name: Bio3D
- Project home page: http://thegrantlab.org/bio3d
- Operating system(s): Platform independent
- Programming language: R
- Other requirements: R > = 3.0.0
- License: GPL2
- Any restrictions to use by non-academics: none

## Additional files

Additional file 1:
**Comprehensive tutorials for traditional single structure and new ensemble NMA on Heterotrimeric G-proteins and other systems.**
Additional file 2:
***E. coli DHFR*** **ensemble NMA and PCA, including a comparison of implemented similarity measures.**
Additional file 3:
**Species wide NMA of the DHFR superfamily.**
Additional file 4:
**Complete example of the integration of ensemble NMA with correlation network analysis.**

## Abbreviations

CNA: Correlation network analysis
DHFR: Dihydrofolate reductase
ENM: Elastic network model
MD: Molecular dynamics
NMA: Normal mode analysis
PCA: Principal component analysis
RMSIP: Root mean square inner product
