# Supplementary material for: Integrating protein structural dynamics and evolutionary analysis with Bio3D
Source: BMC Bioinformatics. 2014 Dec 10;15(1):399. doi: 10.1186/s12859-014-0399-6 (PMC4279791; doi:10.1186/s12859-014-0399-6)
Supplement: Additional file 1: — Comprehensive tutorials for traditional single structure and new ensemble NMA on Heterotrimeric G-proteins and other systems. [file 12859_2014_399_MOESM1_ESM.pdf]

# Enhanced Normal Modes Analysis with Bio3D

*Lars Skjaerven, Xin-Qiu Yao, Guido Scarabelli & Barry J. Grant*

*October 1, 2014*

## Contents

**Background** **2**

Requirements . . . . . 2

About this document . . . . . 2

**1 Example 1: Basic Normal Mode Analysis** **2**

1.1 Example 1A: Normal mode calculation . . . . . 2

1.2 Example 1B: Specifying a force field . . . . . 4

1.3 Example 1C: Normal mode analysis of the GroEL subunit . . . . . 5

**2 Example 2: Ensemble normal mode analysis** **12**

2.1 Example 2A: Dihydrofolate reductase . . . . . 13

2.2 Example 2B: Transducin . . . . . 15

**3 Example 3: Variance weighted normal mode analysis** **16**

3.1 Example 3A: GroEL . . . . . 16

3.2 Example 3B: Transducin . . . . . 23

**4 Example 4: User-defined pair force constant functions** **25**

4.1 Example 4A: Specifying a simple function . . . . . 25

4.2 Example 4B: Specific force constants for disulfide bridges . . . . . 26

**Document Details** **28**

**Information About the Current Bio3D Session** **28**

**References** **28**

This document provides **Additional File 1** for *Integrating protein structural dynamics and evolutionary analysis with Bio3D.*

# Background

Bio3D[1] is an R package that provides interactive tools for structural bioinformatics . The primary focus of Bio3D is the analysis of biomolecular structure, sequence and simulation data (Grant et al. 2006).

Normal mode analysis (NMA) is one of the major simulation techniques used to probe large-scale motions in biomolecules. Typical application is for the prediction of functional motions in proteins. Version 2.0 of the Bio3D package now includes extensive NMA facilities. These include a unique collection of multiple elastic network model (ENM) force-fields (see **Example 1** below), automated ensemble analysis methods (**Example 2**), variance weighted NMA (**Example 3**), and NMA with user-defined force fields (**Example 4**). Here we demonstrate the use of these new features with working code that comprise complete executable examples[2].

## Requirements

Detailed instructions for obtaining and installing the Bio3D package on various platforms can be found in the Installing Bio3D Vignette available both on-line and from within the Bio3D package. In addition to Bio3D the *MUSCLE* multiple sequence alignment program (available from the muscle home page) must be installed on your system and in the search path for executables. Please see the installation vignette for further details.

## About this document

This vignette was generated using **Bio3D version 2.1.0**.

# 1 Example 1: Basic Normal Mode Analysis

## 1.1 Example 1A: Normal mode calculation

Normal mode analysis (NMA) of a single protein structure can be carried out by providing a PDB object to the function **nma()**. In the code below we first load the Bio3D package and then download an example structure of hen egg white lysozyme (PDB id *1hel*) with the function **read.pdb()**. Finally the function **nma()** is used perform the normal mode calculation:

```
library(bio3d)
pdb <- read.pdb("1hel")
```

[1]The latest version of the package, full documentation and further vignettes (including detailed installation instructions) can be obtained from the main Bio3D website: http://thegrantlab.org/bio3d/.

[2]This vignette contains executable examples, see `help(vignette)` for further details.

```
##   Note: Accessing on-line PDB file
##   HEADER    HYDROLASE(O-GLYCOSYL)                   10-JAN-92   1HEL
```

```r
modes <- nma(pdb)
```

```
##  Building Hessian...     Done in 0.047 seconds.
##  Diagonalizing Hessian...    Done in 0.142 seconds.
```

A short summary of the returned *nma* object contained within the new variable *modes* can be obtained by simply calling the function **print()**:

```r
print(modes)
```

```
##
## Call:
##   nma.pdb(pdb = pdb)
##
## Class:
##   VibrationalModes (nma)
##
## Number of modes:
##   387 (6 trivial)
##
## Frequencies:
##   Mode 7:    0.018
##   Mode 8:    0.019
##   Mode 9:    0.024
##   Mode 10:   0.025
##   Mode 11:   0.028
##   Mode 12:   0.029
##
## + attr: modes, frequencies, force.constants, fluctuations,
##         U, L, xyz, mass, temp, triv.modes, natoms, call
```

This reveals the function call resulting in the *nma* object along with the total number of stored normal modes. For PDB id *1hel* there are 129 amino acid residues, and thus 387 modes ($3*129 = 387$) in this object. The first six modes are so-called trivial modes with zero frequency and correspond to rigid-body rotation and translation. The frequency of the next six lowest-frequency modes is also printed.

Note that the returned *nma* object consists of a number of attributes listed on the *+attr:* line. These attributes contain the detailed results of the calculation and their complete description can be found on the **nma()** functions help page accessible with the command: `help(nma)`. To get a quick overview of the results one can simply call the **plot()** function on the returned *nma* object. This will produce a summary plot of (1) the eigenvalues, (2) the mode frequencies, and (3) the atomic fluctuations (See Figure 1).

```r
plot(modes, sse=pdb)
```

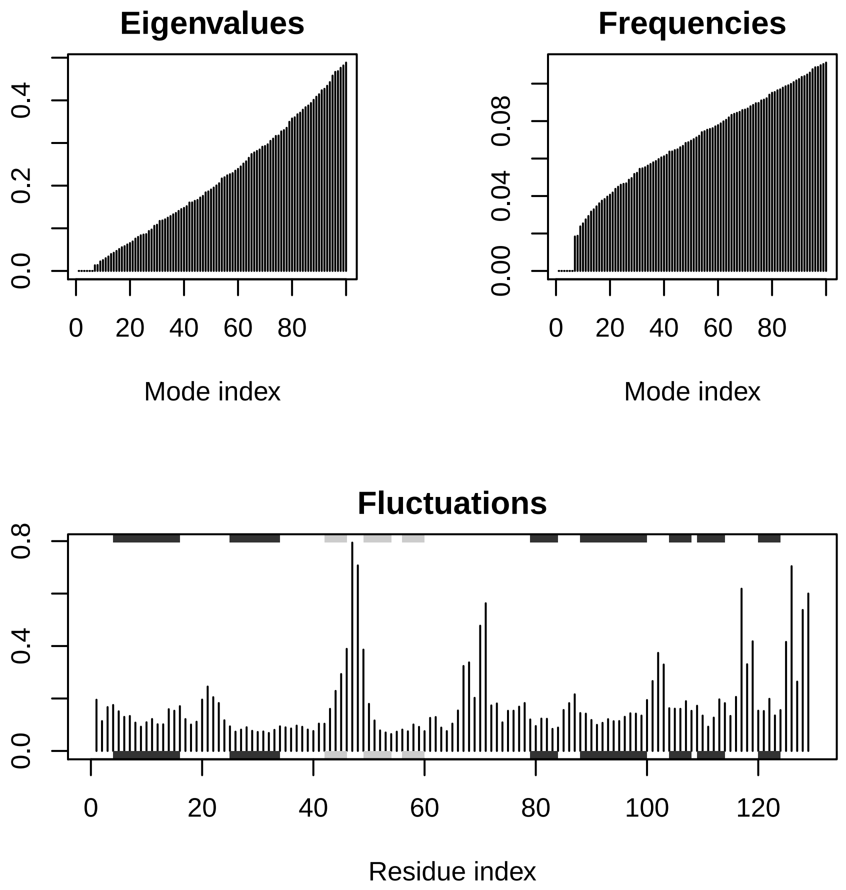


Figure 1: Summary plot of NMA results for hen egg white lysozyme (PDB id *1hel*). The optional `sse=pdb` argument provided to **plot.nma()** results in a secondary structure schematic being added to the top and bottom margins of the fluctuation plot (helices black and strands gray). Note the larger fluctuations predicted for loop regions.

## 1.2 Example 1B: Specifying a force field

The main Bio3D normal mode analysis function, **nma()**, requires a set of coordinates, as obtained from the **read.pdb()** function, and the specification of a force field describing the interactions between constituent atoms. By default the *calpha* force field originally developed by Konrad Hinsen is utilized (Hinsen et al. 2000). This employs a spring force constant differentiating between nearest-neighbor pairs along the backbone and all other pairs. The force constant function was parameterized by fitting to a local minimum of a crambin model using the AMBER94 force field. However, a number of additional force fields are also available, as well as functionality for providing customized force constant functions. Full details of available force fields can be obtained with the command `help(load.enmff)`. With the code below we briefly demonstrate their usage along with a simple comparison of the modes obtained from two of the most commonly used force fields:

```r
help(load.enmff)
```

```r
# Calculate modes with various force fields
modes.a <- nma(pdb, ff="calpha")
modes.b <- nma(pdb, ff="anm")
modes.c <- nma(pdb, ff="pfanm")
modes.d <- nma(pdb, ff="reach")
modes.e <- nma(pdb, ff="sdenm")

# Root mean square inner product (RMSIP)
r <- rmsip(modes.a, modes.b)
```

```r
plot(r, xlab="ANM", ylab="C-alpha FF")
```

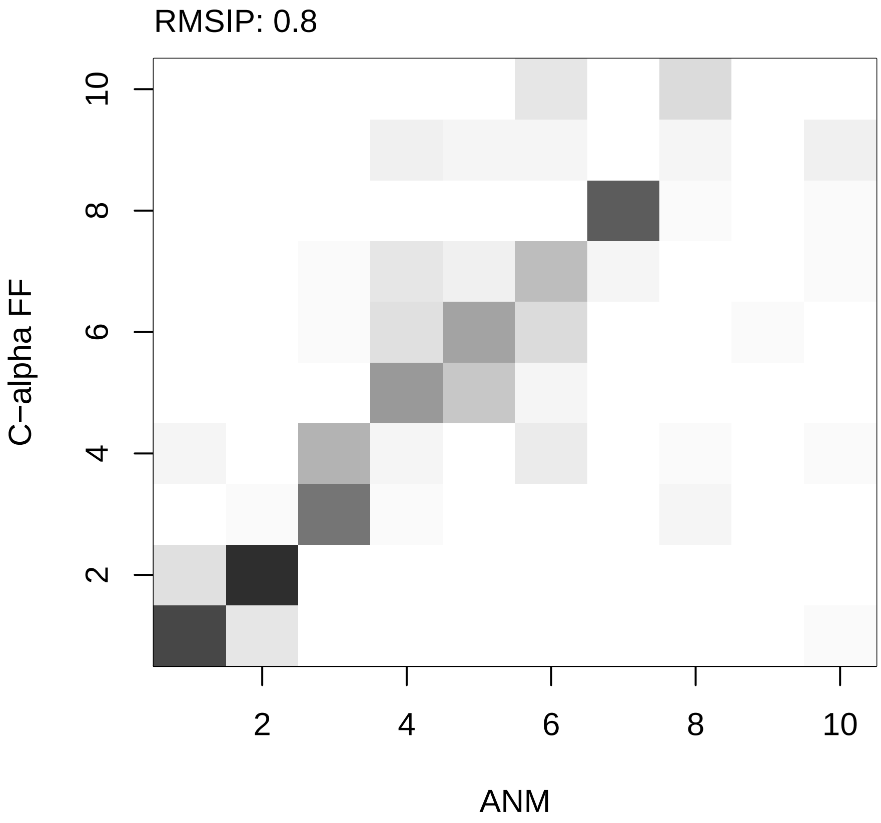


Figure 2: Analysis of mode similarity between modes obtained from the *ANM* and *calpha* force fields by calculating mode overlap and root mean square inner product (RMSIP) with function **rmsip()**. An RMSIP value of *1* depicts identical directionality of the two mode subspaces.

### 1.3 Example 1C: Normal mode analysis of the GroEL subunit

Bio3D includes a number of functions for analyzing and visualizing the normal modes. In the example below we illustrate this functionality on the GroEL subunit. GroEL is a multimeric protein

consisting of 14 identical subunits organized in three distinct domains inter-connected by two hinge regions facilitating large conformational changes.

We will investigate the normal modes through (**1**) mode visualization to illustrate the nature of the motions; (**2**) cross-correlation analysis to determine correlated regions; (**3**) deformation analysis to measure the local flexibility of the structure; (**4**) overlap analysis to determine which modes contribute to a given conformational change; and (**5**) domain analysis to identify regions of the protein moving as rigid parts.

### 1.3.1 Calculate the normal modes

In the code below we download a structure of GroEL (PDB-id *1sx4*) and use **atom.select()** to select one of the 14 subunits prior to the call to **nma()**:

```
# Download PDB, calcualte normal modes of the open subunit
pdb.full   <- read.pdb("1sx4")
pdb.open   <- trim.pdb(pdb.full, atom.select(pdb.full, chain="A"))
modes      <- nma(pdb.open)
```

### 1.3.2 Mode visualization

With Bio3D you can visualize the normal modes either by generating a trajectory file which can be loaded into a molecular viewer program (e.g. VMD or PyMOL), or through a vector field representation in PyMOL. Both functions, **mktrj.nma()** and **view.modes()**, takes an *nma* object as input in addition to the mode index specifying which mode to visualize:

```
# Make a PDB trajectory
mktrj(modes, mode=7)

# Vector field representation (see Figure 3.)
view.modes(modes, mode=7)
```

### 1.3.3 Cross-correlation analysis

Function **dccm.nma()** calculates the cross-correlation matrix of the *nma* object. Function **plot.dccm()** will draw a correlation map, and 3D visualization of correlations is provided through function **view.dccm()**:

```
# Calculate the cross-correlation matrix
cm <- dccm(modes)
```

```
# Plot a correlation map with plot.dccm(cm)
plot(cm, sse=pdb.open, contour=F, col.regions=bwr.colors(20), at=seq(-1,1,0.1) )
```

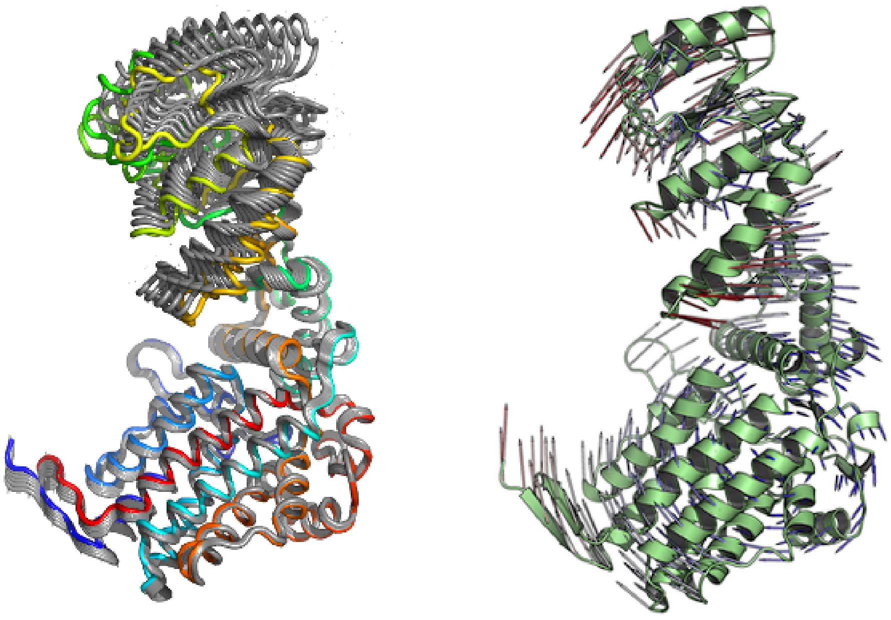

Figure 3: Visualization of the first non-trivial mode of the GroEL subunit. Visualization is provided through a trajectory file (left), or vector field representation (right).

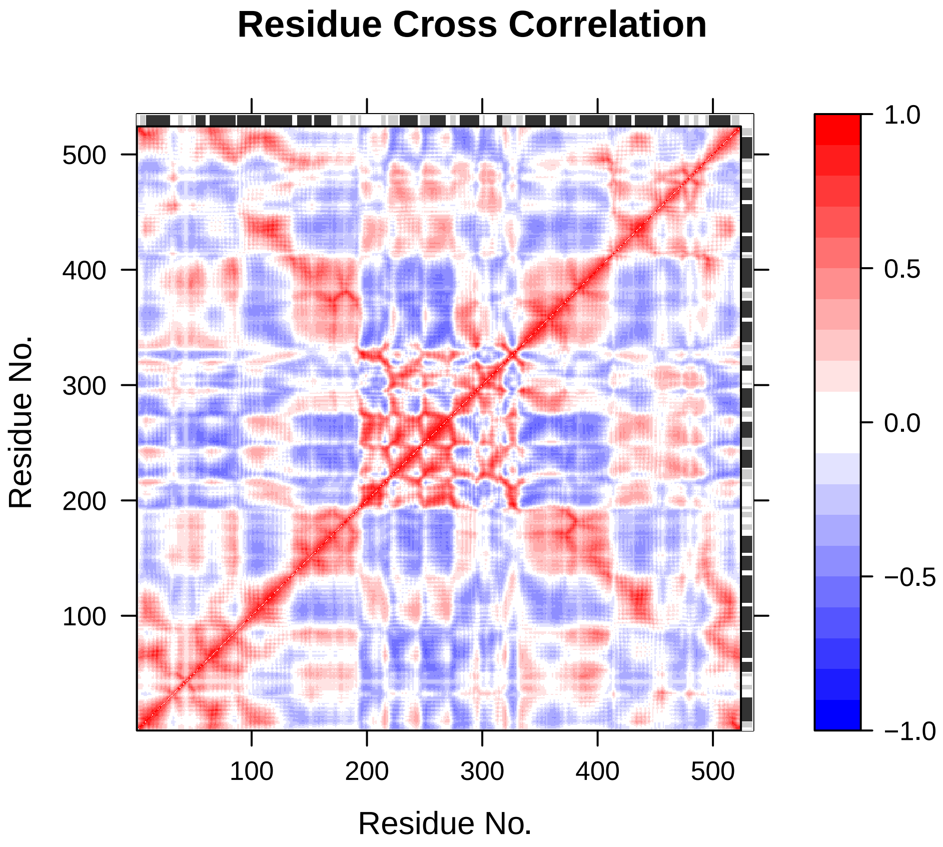


Figure 4: Correlation map revealing correlated and anti-correlated regions in the protein structure.

```r
# View the correlations in the structure (see Figure 5.)
view.dccm(cm, pdb.open, launch=TRUE)
```

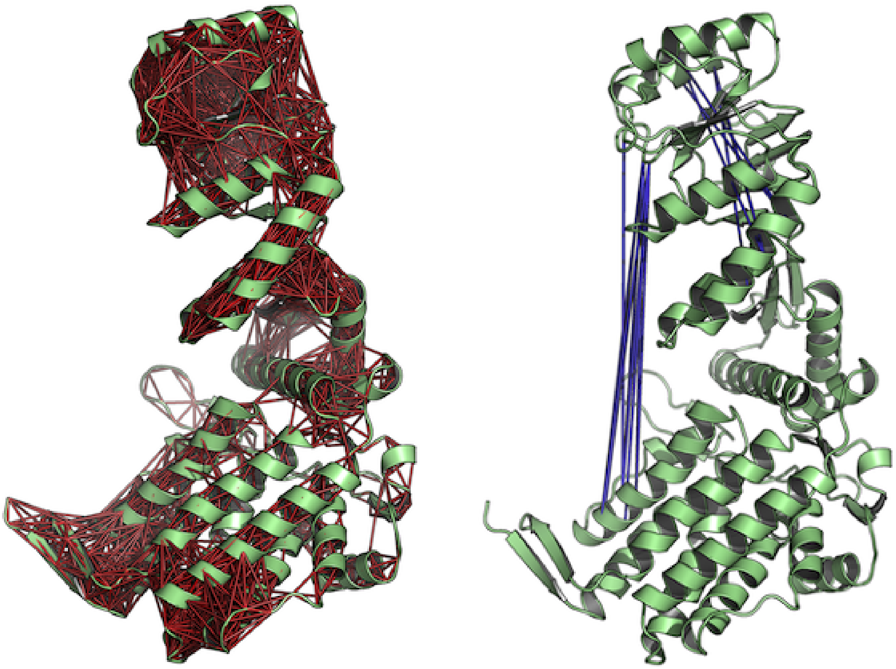

Figure 5: Correlated (left) and anti-correlated (right) residues depicted with red and blue lines, respectively. The figures demonstrate the output of function **view.dccm()**.

### 1.3.4 Fluctuation and Deformation analysis

Deformation analysis provides a measure for the amount of local flexibility in the protein structure - *i.e.* atomic motion relative to neighboring atoms. It differs from *fluctuations* (*e.g.* RMSF values) which provide amplitudes of the absolute atomic motion. Below we calculate deformation energies (with **deformation.nma()**) and atomic fluctuations (with **fluct.nma()**) of the first three modes and visualize the results in PyMOL:

```r
# Deformation energies
defe <- deformation.nma(modes)
defsums <- rowSums(defe$ei[,1:3])

# Fluctuations
flucts <- fluct.nma(modes, mode.inds=seq(7,9))
```

```r
# Write to PDB files (see Figure 6.)
write.pdb(pdb=NULL, xyz=modes$xyz, file="R-defor.pdb", b=defsums)
write.pdb(pdb=NULL, xyz=modes$xyz, file="R-fluct.pdb", b=flucts)
```

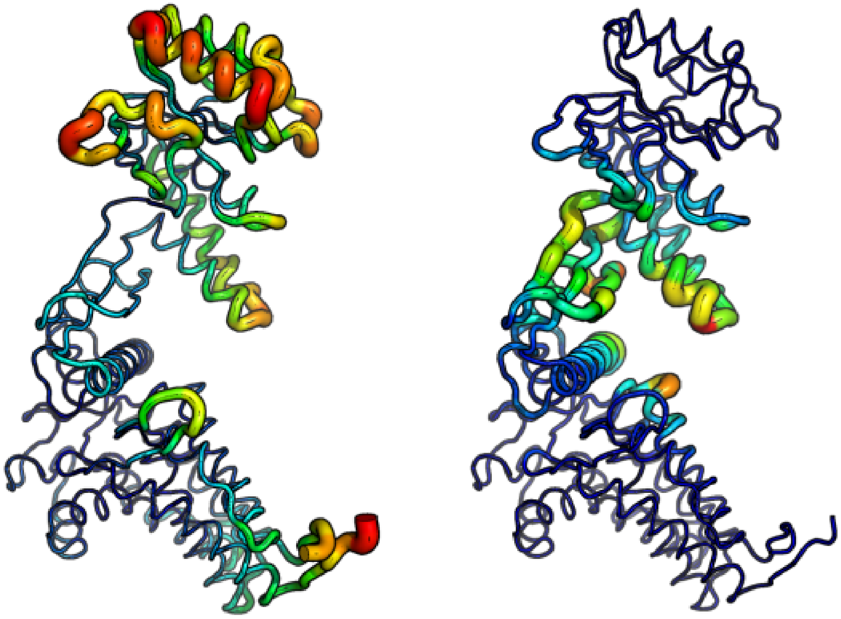

Figure 6: Atomic fluctuations (left) and deformation energies (right) visualized in PyMOL.

### 1.3.5 Overlap analysis

Finally, we illustrate overlap analysis to compare a conformational difference vector with the normal modes to identify which modes contribute to a given conformational change (i.e. the difference between the open and closed state of the GroEL subunit).

```
# Closed state of the subunit
pdb.closed <- trim.pdb(pdb.full, atom.select(pdb.full, chain="H"))

# Align closed and open PDBs
aln <- struct.aln(pdb.open, pdb.closed, max.cycles=0)
pdb.closed$xyz <- aln$xyz

# Caclulate a difference vector
xyz <- rbind(pdb.open$xyz[aln$a.inds$xyz], pdb.closed$xyz[aln$a.inds$xyz])
diff <- difference.vector(xyz)

# Calculate overlap
oa <- overlap(modes, diff)
```

```
plot(oa$overlap, type='h', xlab="Mode index", ylab="Squared overlap", ylim=c(0,1))
points(oa$overlap, col=1)
lines(oa$overlap.cum, type='b', col=2, cex=0.5)
text(c(1,5)+.5, oa$overlap[c(1,5)], c("Mode 1", "Mode 5"), adj=0)
```

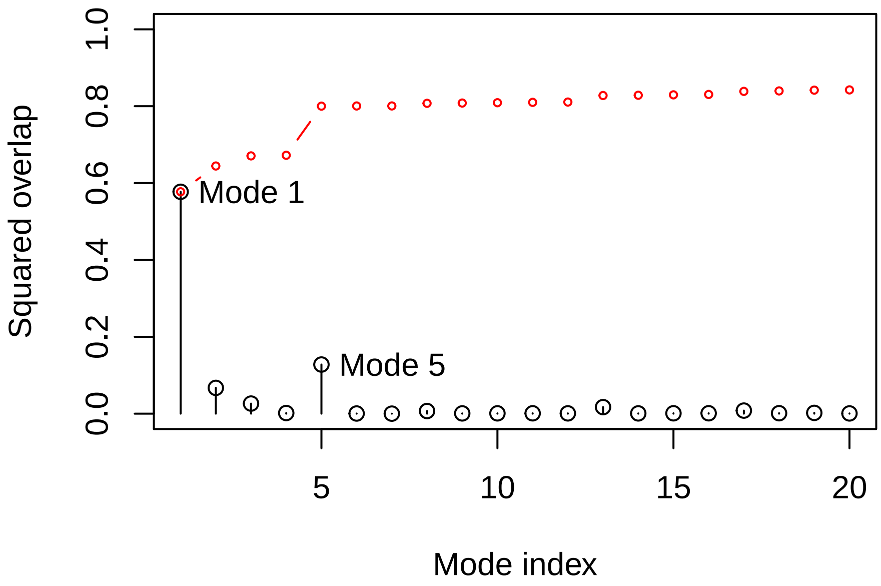


Figure 7: Overlap analysis between the modes of the open subunit and the conformational difference vector between the closed-open state.

#### 1.3.6 Domain analysis with GeoStaS

Identification of regions in the protein that move as rigid bodies is facilitated with the implementation of the GeoStaS algorithm (Romanowska, Nowinski, and Trylska 2012). Below we demonstrate the use of function **geostas()** on an *nma* object, and an ensemble of X-ray structures. See `help(geostas)` for more details and further examples.

**GeoStaS with NMA**: Starting from the calculated normal modes, we generate conformers by interpolating along the eigenvectors of the first 5 normal modes of the GroEL subunit. We then use this ensemble as input to function **geostas()**:

```
# Build conformational ensemble
trj <- rbind(mktrj(modes, mode=7)[10:24,],
             mktrj(modes, mode=8)[10:24,],
             mktrj(modes, mode=9)[10:24,],
             mktrj(modes, mode=10)[10:24,],
             mktrj(modes, mode=11)[10:24,])

# Run geostas to find domains
gs <- geostas(trj, k=4)
```

```
# Write NMA trajectory with domain assignment
mktrj(modes, mode=7, chain=gs$grps)
```

**GeoStaS with X-ray structure ensemble**: Alternatively the same analysis can be performed on an ensemble of X-ray structures obtained from the PDB:

```
# Define the ensemble PDB-ids
ids <- c("1sx4_[A,B,H,I]", "1xck_[A-B]", "1sx3_[A-B]", "4ab3_[A-B]")

# Download and split PDBs by chain ID
raw.files <- get.pdb(ids, "groel_pdbs", gzip=TRUE)
files <- pdbsplit(raw.files, ids, path = "groel_pdbs")

# Align and superimpose coordinates
pdbs <- pdbaln(files, fit=TRUE)

# Run geostast to find domains
gs <- geostas(pdbs, k=4)
```

```
# Plot a atomic movement similarity matrix
plot.dccm(gs$amsm, at=seq(0, 1, 0.1), main="AMSM with Domain Assignment",
          col.regions=rev(heat.colors(200)), margin.segments=gs$grps, contour=FALSE)
```

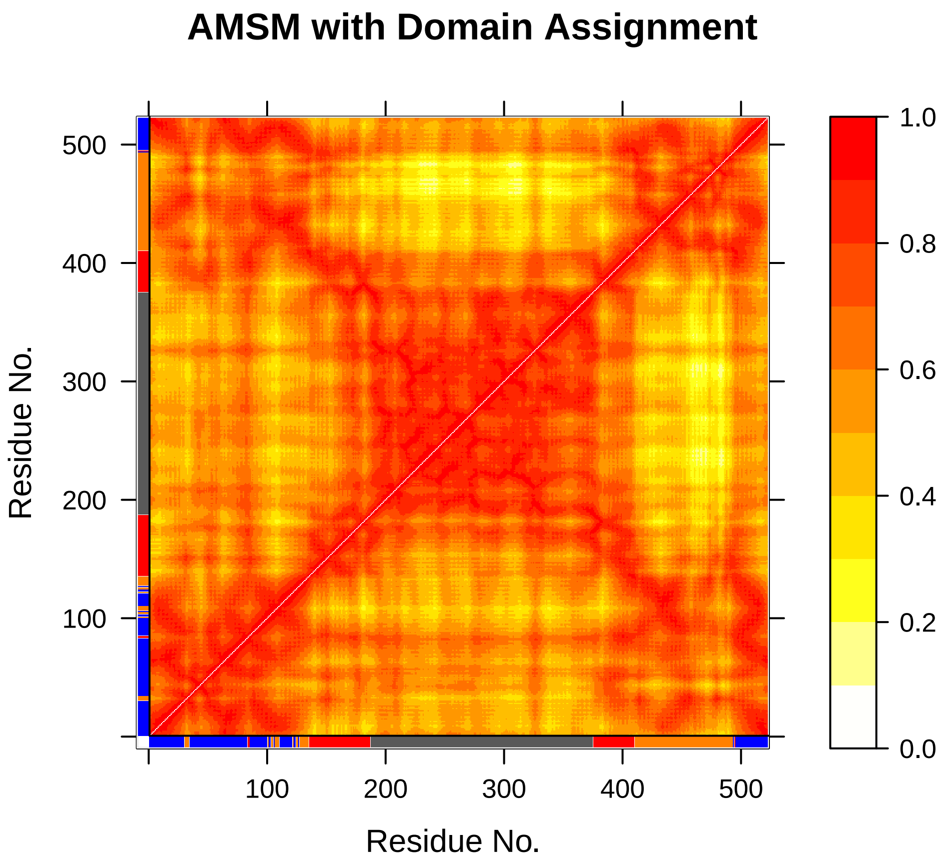


Figure 8: Atomic movement similarity matrix with domain annotation.

```
# Principal component analysis
gaps.pos <- gap.inspect(pdbs$xyz)
xyz <- fit.xyz(pdbs$xyz[1, gaps.pos$f.inds],
```

```r
                pdbs$xyz[, gaps.pos$f.inds],
                fixed.inds=gs$fit.inds,
                mobile.inds=gs$fit.inds)

pc.xray <- pca.xyz(xyz)
```

```r
# Visualize PCs with colored domains (chain ID)
mktrj(pc.xray, pc=1, chain=gs$grps)
```

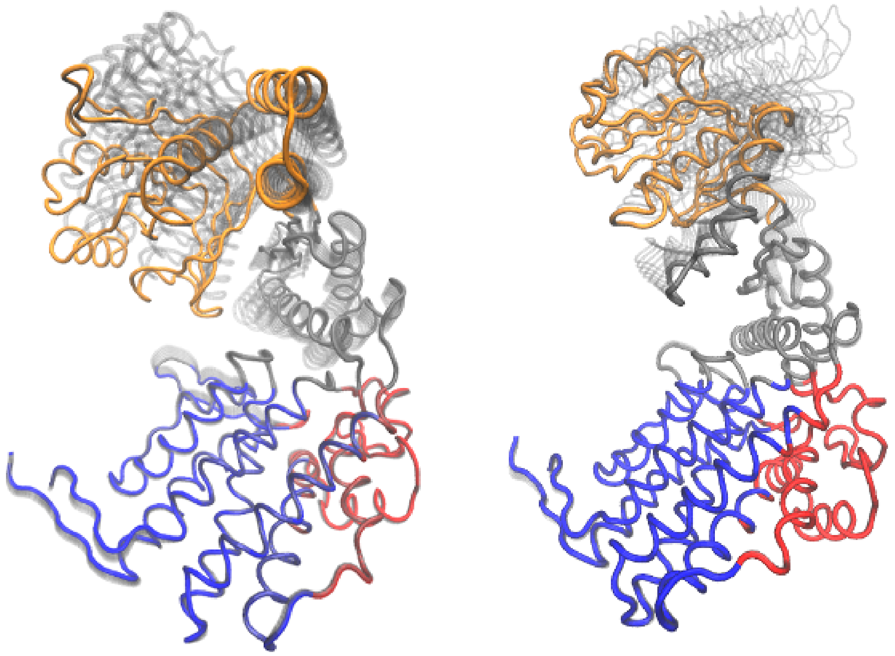

Figure 9: Visualization of domain assignment obtained from function **geostas()** using (left) an ensemble of X-ray structures and (right) NMA.

# 2 Example 2: Ensemble normal mode analysis

The analysis of multiple protein structures (e.g. a protein family) can be accomplished with the **nma.pdbs()** function.[3] This will take aligned input structures, as generated by the **pdbaln()** function for example, and perform NMA on each structure collecting the results in manner that facilitates the interpretation of similarity and dissimilarity trends in the structure set. Here we will analyze a collection of Dihydrofolate reductase (DHFR) structures with low sequence identity (**Example 2A**) and large set of closely related transducin heterotrimeric G protein family members (**Example 2B**).

[3]See also dedicated vignettes for *ensemble NMA* provided with the Bio3D package.

## 2.1 Example 2A: Dihydrofolate reductase

In the following code we collect 9 bacterial DHFR structures of 4 different species from the protein databank (using **get.pdb()**) with sequence identity down to 27% (see the call to function **seqidentity()** below), and align these with **pdbaln()**:

```
# Select bacterial DHFR PDB IDs
ids <- c("1rx2_A", "1rx4_A", "1rg7_A",
         "3jw3_A", "3sai_A",
         "1df7_A", "4kne_A",
         "3fyv_X", "3sgy_B")

# Download and split by chain ID
raw.files <- get.pdb(ids, path="raw_pdbs")
files     <- pdbsplit( raw.files, ids )

# Alignment of structures
pdbs <- pdbaln(files)
```

```
# Sequence identity
summary( c(seqidentity(pdbs)) )
```

```
##    Min. 1st Qu.  Median    Mean 3rd Qu.    Max.
##   0.268   0.346   0.396   0.527   0.994   1.000
```

The *pdbs* object now contains *aligned* C-alpha atom data, including Cartesian coordinates, residue numbers, residue types, and B-factors. The sequence alignment is also stored by default to the FASTA format file 'aln.fa' (to view this you can use an alignment viewer such as SEAVIEW, see *Requirements* section above). Function **nma.pdbs()** will calculate the normal modes of each protein structures stored in the *pdbs* object. The normal modes are calculated on the full structures as provided by object *pdbs*. With the default argument `rm.gaps=TRUE` unaligned atoms are omitted from output in accordance with common practice (Fuglebakk, Echave, and Reuter 2012).

```
# NMA on all structures
modes <- nma(pdbs)
```

The *modes* object of class *enma* contains aligned normal mode data including fluctuations, RMSIP data, and aligned eigenvectors. A short summary of the *modes* object can be obtain by calling the function **print()**, and the aligned fluctuations can be plotted with function **plot()**:

```
print(modes)
```

```
##
## Call:
##   nma.pdbs(pdbs = pdbs)
##
```

```
## Class:
##   enma
##
## Number of structures:
##   9
##
## Attributes stored:
##   - Root mean square inner product (RMSIP)
##   - Aligned atomic fluctuations
##   - Aligned eigenvectors (gaps removed)
##   - Dimensions of x$U.subspace: 456x450x9
##
## Coordinates were aligned prior to NMA calculations
##
## + attr: fluctuations, rmsip, U.subspace, L, full.nma, xyz,
##         call
```

```
# Plot fluctuation data
col <- c(1,1,1, 2,2, 3,3, 4,4)
plot(modes, pdbs=pdbs, col=col)
legend("topleft", col=unique(col), lty=1,
       legend=c("E.Coli", "B.Anthracis", "M.Tubercolosis", "S.Aureus"))
```

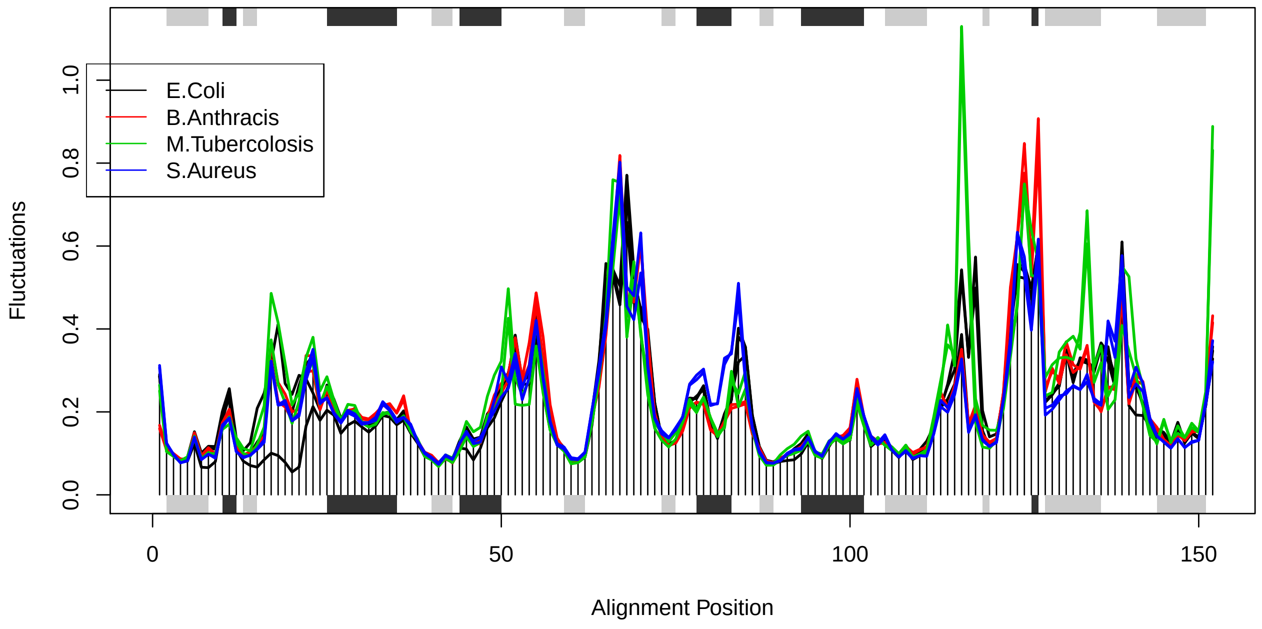


Figure 10: Results of ensemble NMA on four distinct bacterial species of the DHFR enzyme.

```
# Alternatively, one can use 'rm.gaps=FALSE' to keep the gap containing columns
modes <- nma.pdbs(pdbs, rm.gaps=FALSE)
```

Cross-correlation analysis can be easily performed and the results contrasted for each member of the input ensemble. Below we calculate and plot the correlation matrices for each structure and

then output correlations present only in all input structures.

```
# Calculate correlation matrices for each structure
cij <- dccm(modes)
```

```
# Determine correlations present only in all 9 input structures
cij.all <- filter.dccm(cij$all.dccm, cutoff.sims=9, cutoff.cij = 0)
plot.dccm(cij.all, main="Consensus Residue Cross Correlation")
```

## 2.2 Example 2B: Transducin

In this section we will demonstrate the use of **nma.pdbs()** on the example transducin family data that ships with the Bio3D package. This can be loaded with the command *data(transducin)* and contains an object *pdbs* consisting of aligned C-alpha coordinates for 53 transducin structures from the PDB as well their annotation (in the object *annotation*) as obtained from the **pdb.annotate()** function. Note that this data can be generated from scratch by following the *Comparative Structure Analysis with Bio3D Vignette* available both on-line and from within the Bio3D package.

```
# Load data
data(transducin)
pdbs <- transducin$pdbs
annotation <- transducin$annotation

# Find gap positions
gaps.res <- gap.inspect(pdbs$ali)
gaps.pos <- gap.inspect(pdbs$xyz)
```

```
# Calculate normal modes of the 53 structures
modes <- nma.pdbs(pdbs, ncore=4)
```

```
# Make fluctuation plot
plot(modes, col=annotation[, "color"], pdbs=pdbs)
legend("left", lty=c(1, 1), lwd=c(2, 2),
    col=c("red", "green"), legend=c("GTP", "GDP"))
```

The similarity of structural dynamics is calculated by RMSIP based on the 10 lowest frequency normal modes. The *rmsip* values are pre-calculated in the *modes* object and can be accessed through the attribute `modes$rmsip`. As a comparison, we also calculate the root mean square deviation (RMSD) of all pair-wise structures:

```
# Plot a heat map with clustering dendogram
ids <- substr(basename(pdbs$id), 1, 6)
heatmap((1-modes$rmsip), labRow=annotation[, "state"], labCol=ids, symm=TRUE)
```

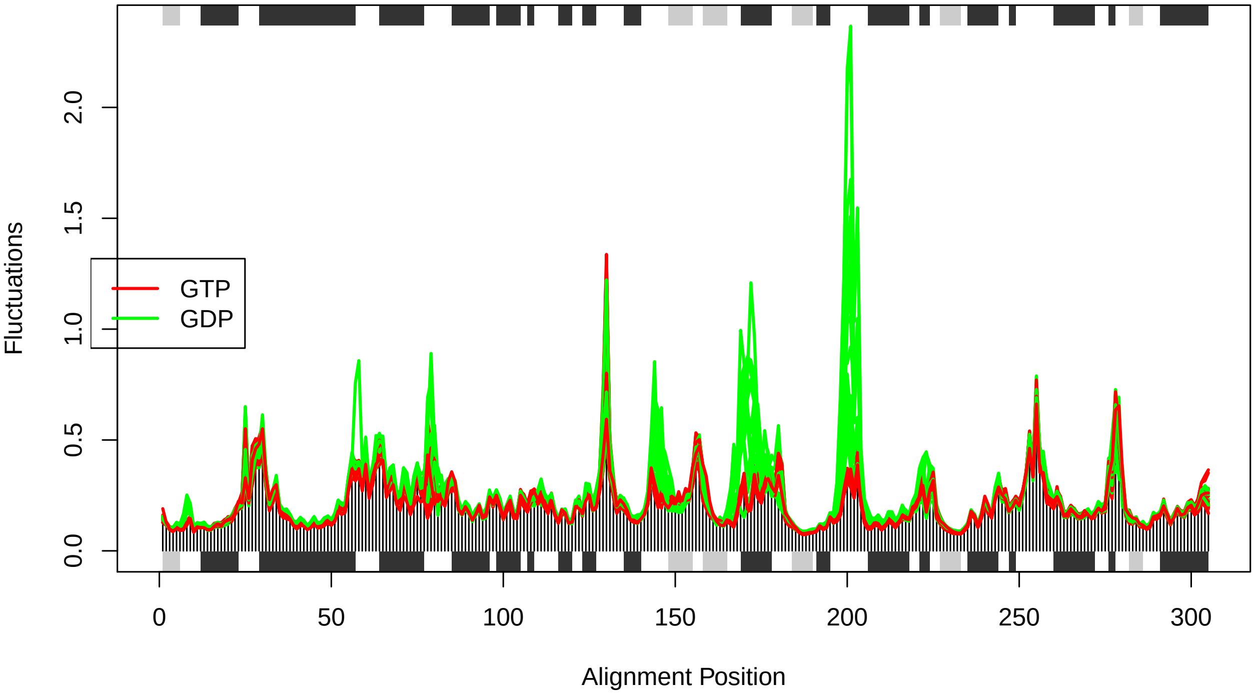


Figure 11: Structural dynamics of transducin. The calculation is based on NMA of 53 structures: 28 GTP-bound (red), and 25 GDP-bound (green).

```
# Calculate pair-wise RMSD values
rmsd.map <- rmsd(pdbs$xyz, a.inds=gaps.pos$f.inds, fit=TRUE)
heatmap(rmsd.map, labRow=annotation[, "state"], labCol=ids, symm=TRUE)
```

# 3 Example 3: Variance weighted normal mode analysis

In this example we illustrate an approach of weighting the pair force constants based on the variance of the inter atomic distances obtained from an ensemble of structures (e.g. available X-ray structures). The motivation for such variance-weighting is to reduce the well known dependence of the force constants on the one structure upon which they are derived (Tama and Sanejouand 2001).

## 3.1 Example 3A: GroEL

We first calculate the normal modes of both the closed and open state of the GroEL subunit, and we illustrate the difference in the agreement towards the observed conformational changes (characterized by X-ray and EM studies). We will then use an ensemble of X-ray/EM structures as weights to the pair-force constants.

```
# Define the ensemble PDB-ids
ids <- c("1sx4_[A,B,H,I]", "1xck_[A-B]", "1sx3_[A-B]", "4ab3_[A-B]")
```

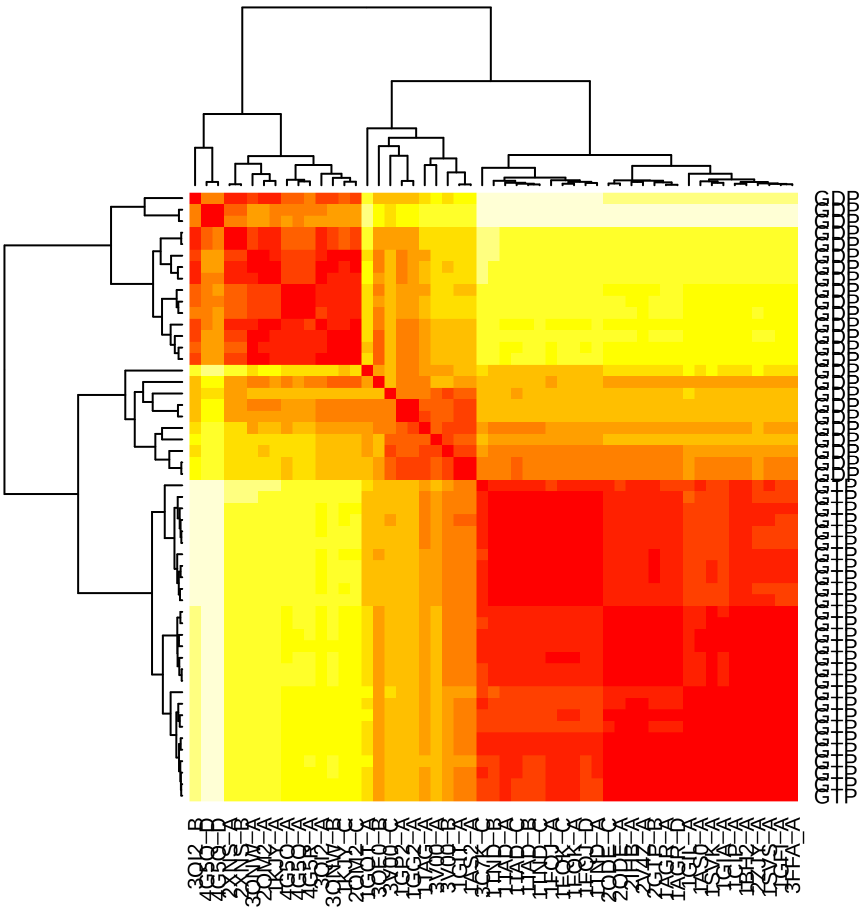

Figure 12: RMSIP matrix of the transducin family.

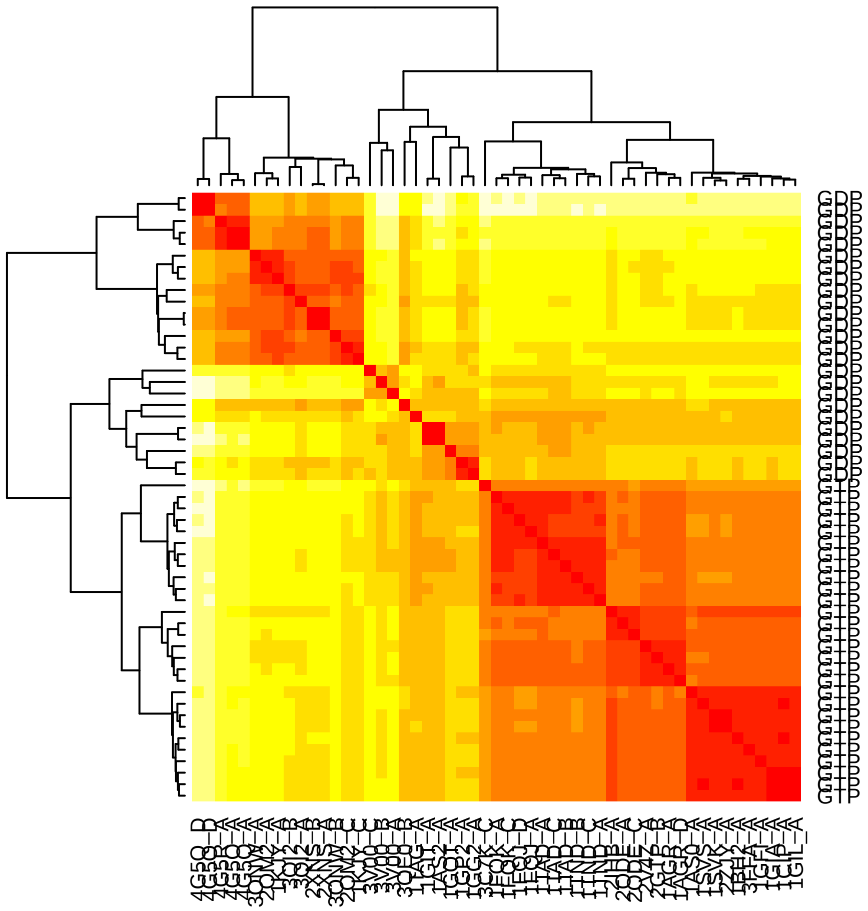

Figure 13: RMSD matrix of the transducin family.

```
# Download and split PDBs by chain ID
raw.files <- get.pdb(ids, "groel_pdbs", gzip=TRUE)
files <- pdbsplit(raw.files, ids, path = "groel_pdbs")

# Align and superimpose coordinates
pdbs <- pdbaln(files, fit=TRUE)
```

### 3.1.1 Calculate normal modes

Next we will calculate the normal modes of the open and closed conformational state. They are stored at indices 1 and 5, respectively, in our *pdbs* object. Use the **pdbs2pdb()** to fetch the pdb objects which is needed for the input to **nma()**.

```
# Inspect gaps
gaps.res <- gap.inspect(pdbs$ali)
gaps.pos <- gap.inspect(pdbs$xyz)

# Access PDB objects
pdb.list   <- pdbs2pdb(pdbs, inds=c(1,5,9), rm.gaps=TRUE)
```

Note that we are here using the argument `rm.gaps=TRUE` to omit residues in gap containing columns of the alignment. Consequently, the resulting three pdb objects we obtain will have the same lengths (523 residues), which is convenient for subsequent analysis.

```
pdb.open   <- pdb.list[["1sx4_A"]]
pdb.closed <- pdb.list[["1xck_A"]]
pdb.rstate <- pdb.list[["4ab3_A"]]

# Calaculate normal modes
modes.open   <- nma(pdb.open)
modes.closed <- nma(pdb.closed)
modes.rstate <- nma(pdb.rstate)
```

### 3.1.2 Overlap analysis

Use overlap analysis to determine the agreement between the normal mode vectors and the conformational difference vector:

```
# Difference vector 1: closed - open
diff.vec.1 <- difference.vector(pdbs$xyz[c(1,5), gaps.pos$f.inds])
# Difference vector 2: closed - rstate
diff.vec.2 <- difference.vector(pdbs$xyz[c(5,9), gaps.pos$f.inds])

# Calculate overlap
oa <- overlap(modes.open,   diff.vec.1)
```

```
ob <- overlap(modes.closed, diff.vec.1)
oc <- overlap(modes.closed, diff.vec.2)
```

```
plot(oa$overlap.cum[1:10], type='b', ylim=c(0,1),
     ylab="Squared overlap", xlab="Mode index", lwd=2)
lines(ob$overlap.cum[1:10], type='b', lty=2, col=2, lwd=2)
lines(oc$overlap.cum[1:10], type='b', lty=3, col=4, lwd=1)

legend("bottomright",
       c("Open to closed", "Closed to open", "Closed to r-state"),
       col=c(1,2,4), lty=c(1,2,3))
```

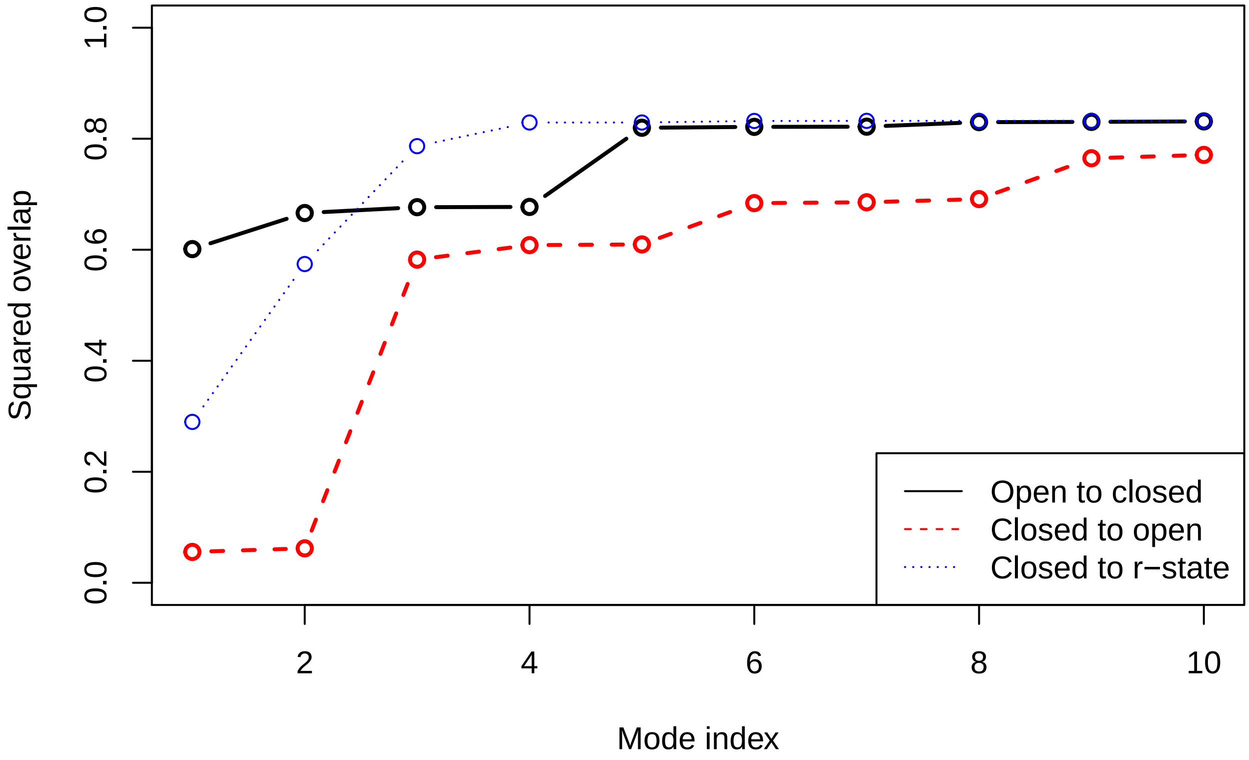


Figure 14: Overlap anlaysis with function **overlap()**. The modes calculated on the open state of the GroEL subunit shows a high similarity to the conformational difference vector (black), while the agreement is lower when the normal modes are calculated on the closed state (red). Blue line correspond to the overlap between the closed state and the r-state (a semi-open state characterized by a rotation of the apical domain in the opposite direction as compared to the open state.

#### 3.1.3 Variance weighting

From the overlap analysis above we see the good agreement (high overlap value) between the conformational difference vector and the normal modes calculated on the open structures. Contrary, the lowest frequency modes of the closed structures does not show the same behavior. We will thus

proceed with the weighting of the force constants. First we'll define a quick function for calculating the weights which takes a matrix of Cartesian coordinates as input:

```
"make.weights" <- function(xyz) {
  # Calculate pairwise distances
  natoms <- ncol(xyz) / 3
  all <- array(0, dim=c(natoms,natoms,nrow(xyz)))
  for( i in 1:nrow(xyz) ) {
    dists <- dist.xyz(xyz[i,])
    all[,,i] <- dists
  }

  # Calculate variance of pairwise distances
  all.vars <- apply(all, 1:2, var)

  # Make the final weights
  weights <- 1 - (all.vars / max(all.vars))
  return(weights)
}

# Calcualte the weights
wts <- make.weights(pdbs$xyz[, gaps.pos$f.inds])
```

Weights to the force constants can be included by the argument 'fc.weights' to function **nma()**. This needs be a matrix with dimensions NxN (where N is the number of C-alpha atoms). Here we will run a small for-loop with increasing the strength of the weighting at each step and store the new overlap values in the variable 'ob.wtd':

```
ob.wtd <- NULL
for ( i in 1:10 ) {
  modes.wtd <- nma(pdb.closed, fc.weights=wts**i)
  ob.tmp    <- overlap(modes.wtd,    diff.vec.1)
  ob.wtd    <- rbind(ob.wtd, ob.tmp$overlap.cum)
}
```

```
plot(oa$overlap.cum[1:10], type='b', ylim=c(0,1),
     ylab="Squared overlap", xlab="Mode index", axes=T, lwd=2)
lines(ob$overlap.cum[1:10], type='b', lty=2, col=1, lwd=2)

cols <- rainbow(10)
for ( i in 1:nrow(ob.wtd) ) {
  lines(ob.wtd[i,1:10], type='b', lty=1, col=cols[i])
}

legend("bottomright",
       c("Open state", "Closed state", "Closed state (weighted)"),
       col=c("black", "black", "green"), lty=c(1,2,1))
```

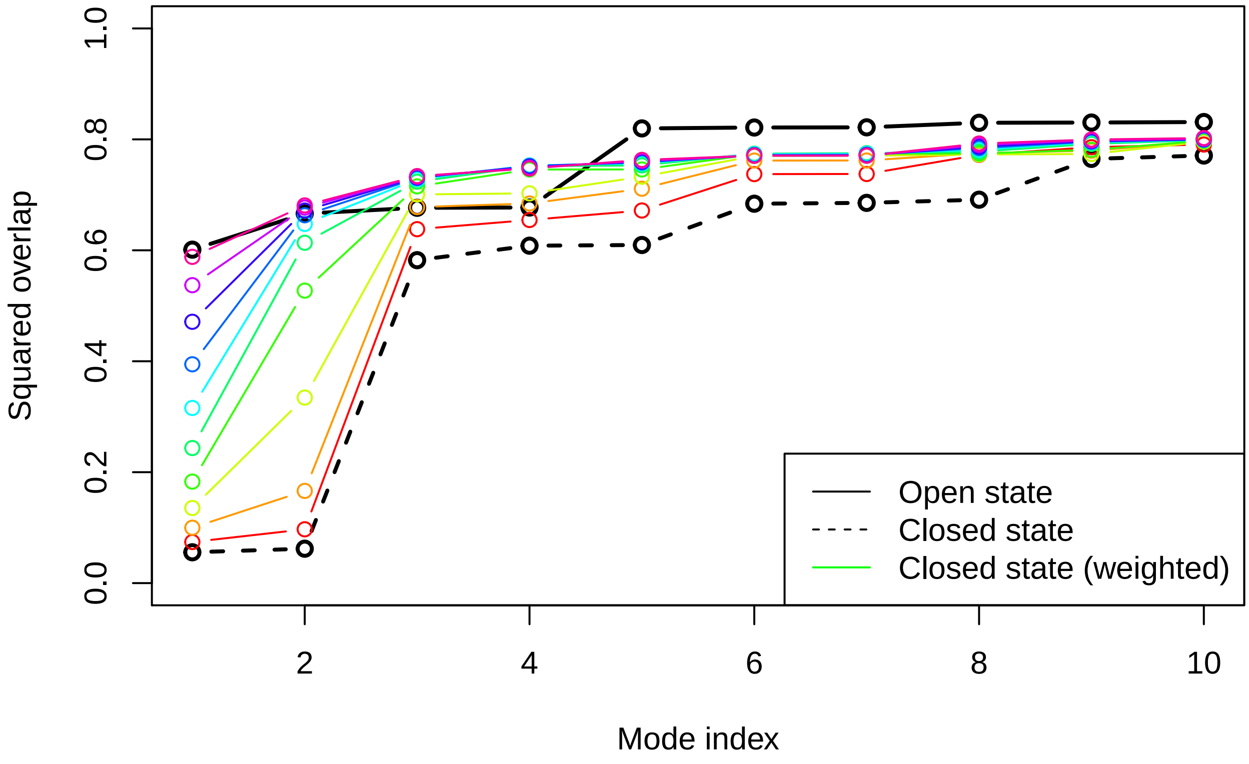


Figure 15: Overlap plot with increasing strength on the weighting. The final weighted normal modes of the closed subunit shows as high overlap values as the modes for the open state.

#### 3.1.4 RMSIP calculation

RMSIP can be used to compare the mode subspaces:

```
ra <- rmsip(modes.open, modes.wtd)
rb <- rmsip(modes.open, modes.closed)
```

```
par(mfrow=c(1,2))
plot(ra, ylab="NMA(open)", xlab="NMA(weighted)")
plot(rb, ylab="NMA(open)", xlab="NMA(closed)")
```

#### 3.1.5 Match with PCA

Finally, we compare the calculated normal modes with principal components obtained from the ensemble of X-ray structures using function **pca.xyz()**:

```
# Calculate the PCs
pc.xray <- pca.xyz(pdbs$xyz[,gaps.pos$f.inds])
```

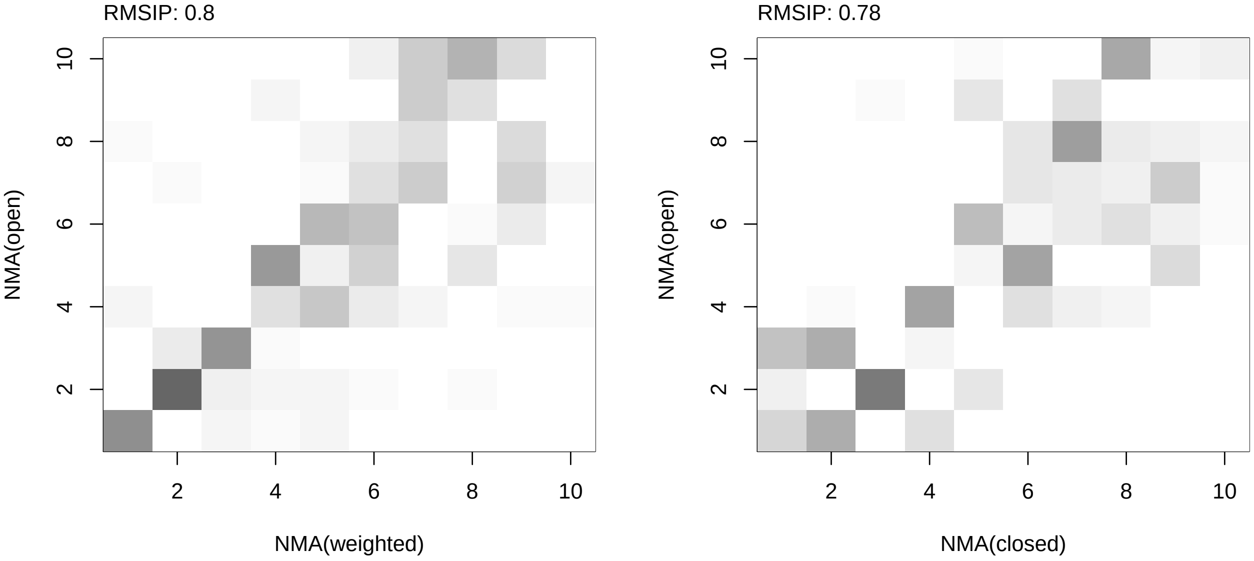


Figure 16: RMSIP maps between (un)weighted normal modes obtained from the open and closed subunits.

```
# or alternatively...
pc.xray <- pca(pdbs)

# Calculate RMSIP values
rmsip(pc.xray, modes.open)$rmsip
```

```
## [1] 0.6226
```

```
rmsip(pc.xray, modes.closed)$rmsip
```

```
## [1] 0.632
```

```
rmsip(pc.xray, modes.rstate)$rmsip
```

```
## [1] 0.589
```

```
rmsip(pc.xray, modes.wtd)$rmsip
```

```
## [1] 0.6617
```

### 3.2 Example 3B: Transducin

This example will run **nma()** on transducin with variance weighted force constants. The modes predicted by NMA will be compared with principal component analysis (PCA) results over the

transducin family. We load the transducin data via the command *data(transducin)* and calculate the normal modes for two structures corresponding to two nucleotide states, respectively: GDP (PDB id 1TAG) and GTP (PDB id 1TND). Again we use function **pdbs2pdb()** to build the *pdb* objects from the *pdbs* object (containing aligned structure/sequence information). The coordinates of the data set were fitted to all non-gap containing C-alpha positions.

```
data(transducin)
pdbs <- transducin$pdbs

gaps.res <- gap.inspect(pdbs$ali)
gaps.pos <- gap.inspect(pdbs$xyz)
```

```
# Fit coordinates based on all non-gap positions
# and do PCA
xyz <- pdbfit(pdbs)
pc.xray <- pca.xyz(xyz[, gaps.pos$f.inds])
```

```
# Fetch PDB objects
npdbs <- pdbs
npdbs$xyz <- xyz
pdb.list <- pdbs2pdb(npdbs, inds=c(2, 7), rm.gaps=TRUE)
pdb.gdp <- pdb.list[[ grep("1TAG_A", names(pdb.list)) ]]
pdb.gtp <- pdb.list[[ grep("1TND_B", names(pdb.list)) ]]

# Calculate normal modes
modes.gdp <- nma(pdb.gdp)
modes.gtp <- nma(pdb.gtp)
```

Now, we calculate the pairwise distance variance based on the structural ensemble with the function **make.weights()** defined above. This will be used to weight the force constants in the elastic network model.

```
# Calculate weights
weights <- make.weights(xyz[, gaps.pos$f.inds])

# Calculate normal modes with weighted pair force constants
modes.gdp.b <- nma(pdb.gdp, fc.weights=weights**100)
modes.gtp.b <- nma(pdb.gtp, fc.weights=weights**100)
```

To evaluate the results, we calculate the overlap (square dot product) between modes predicted by variance weighted or non-weighted NMA and the first principal component from PCA.

```
oa <- overlap(modes.gdp, pc.xray$U[,1])
ob <- overlap(modes.gtp, pc.xray$U[,1])
oc <- overlap(modes.gdp.b, pc.xray$U[,1])
od <- overlap(modes.gtp.b, pc.xray$U[,1])
```

```
plot(oa$overlap.cum, type='o', ylim=c(0,1), col="darkgreen", lwd=2, xlab="Mode",
     ylab="Cummulative overlap")
lines(ob$overlap.cum, type='o', ylim=c(0,1), col="red", lwd=2)
lines(oc$overlap.cum, type='b', ylim=c(0,1), col="darkgreen", lwd=2, lty=2)
lines(od$overlap.cum, type='b', ylim=c(0,1), col="red", lwd=2, lty=2)
text(20, oa$overlap.cum[20], label=round(oa$overlap.cum[20], 2), pos=3)
text(20, ob$overlap.cum[20], label=round(ob$overlap.cum[20], 2), pos=3)
text(20, oc$overlap.cum[20], label=round(oc$overlap.cum[20], 2), pos=3)
text(20, od$overlap.cum[20], label=round(od$overlap.cum[20], 2), pos=3)
legend("topleft", pch=1, lty=c(1, 1, 2, 2), col=c("darkgreen", "red",
       "darkgreen", "red"), legend=c("GDP", "GTP", "Weighted GDP", "Weighted GTP"))
```

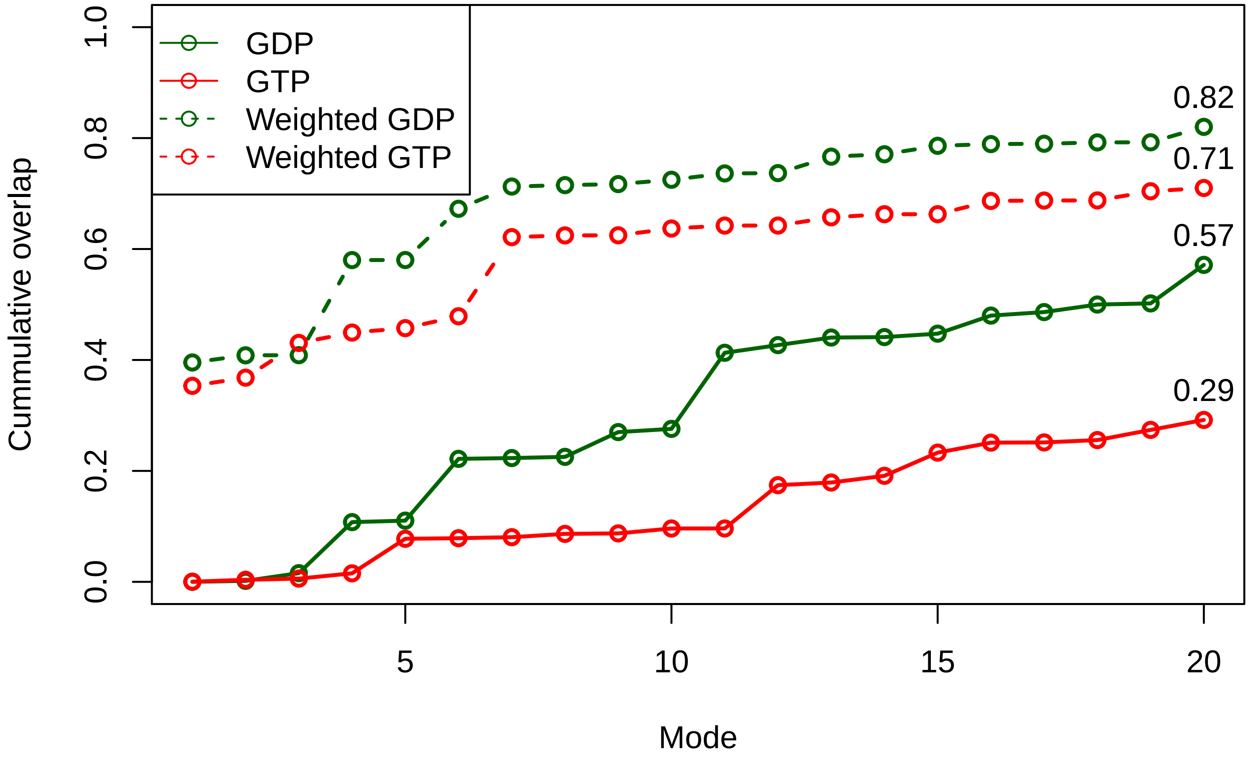


Figure 17: Variance weighted force constants improve NMA prediction

# 4 Example 4: User-defined pair force constant functions

In this example we demonstrate the interface for defining custom functions for the pair spring force constants. A custom function can be obtained through simple scripting as shown below.

## 4.1 Example 4A: Specifying a simple function

We first show how to define a simple force constant function by building a revised version of the parameter-free ANM force field. The function **my.ff()** below takes as input $r$ which is a vector of

inter-atomic (calpha) distances (i.e. distances from atom $i$, to all other atoms in the system; this function will thus be called N times, where N is the number of calpha atoms). It will in this case return 0 for the pairs with a distance larger than 10 Å, and $r^{-2}$ for all other pairs. Our simple function will thus look like:

```
# Define function for spring force constants
"my.ff" <- function(r, ...) {
  ifelse( r>10, 0, r^(-2) )
}
```

Once the function is in place we can feed it to function **nma()** to calculate the normal modes based on the particular force constants built with our new function. Below we apply it to the lysozyme structure (PDB id *1hel*) from Example 1:

```
# Download PDB and calculate normal modes
pdb <- read.pdb("1hel")
modes <- nma(pdb, pfc.fun=my.ff)
```

Alternatively we can take a more manual approach by calling **build.hessian()** if we want to investigate the Hessian matrix further (note that **build.hessian** is called from within function **nma()** which will diagonalize the hessian to obtain the normal modes and thus not return it to the user). In the code below we first build the hessian and illustrate how to obtain the normal modes through calls to either **eigen()** or **nma()** (which can also take a Hessian matrix as input):

```
# Indices for CA atoms
ca.inds <- atom.select(pdb, 'calpha')

# Build hessian matrix
h <- build.hessian(pdb$xyz[ ca.inds$xyz ], pfc.fun=my.ff)

# Diagonalize and obtain eigenvectors and eigenvalues
modes <- eigen(h, symmetric=TRUE)

# ... or feed the Hessian to function 'nma()'
modes <- nma(pdb, hessian=h, mass=FALSE)
```

Note that function **nma()** assumes the Hessian to be mass-weighted and we therefore have to specify *mass=FALSE* in this particular case. To obtain a mass-weighted Hessian pass the amino acid masses through argument *aa.mass* to function **build.hessian()**.

## 4.2 Example 4B: Specific force constants for disulfide bridges

In the following code we illustrate a more advanced force constant function making use of arguments *atom.id* and *ssdat* which is passed from function **build.hessian()** by default. This allows users to access the protein sequence (`ssdat$seq`), secondary structure data (`ssdat$sse`), beta bridges (`ssdat$beta.bridges`), helix 1-4 (`ssdat$helix14`), and disulfide bridges (ss bonds; `ssdat$ss.bonds`) when building the force constants.

First we define our new function (**ff.custom()**) and specify the force constants which should be applied to bonded and non-bonded interactions (`k.bonded` and `k.nonbonded`, respectively). Next we define the the force constant for the disulfide bridges (`k.ssbond`):

```
"ff.custom" <- function(r, atom.id, ssdat=NULL, ...) {
  # Default force constants (Hinsen et al 2000)
  k.bonded    <- (r * 8.6 * 10^2) - (2.39 * 10^3)
  k.nonbonded <- (128 * 10^4) * r^(-6)

  # Special force constant for SS-bonds
  k.ssbond    <- 143;

  # Calculate default values (equivalent to the calpha ff)
  ks <- ifelse(r<4.0,
               k.bonded,
               k.nonbonded)

  if(!is.null(ssdat$ss.bonds)) {
    # If atom.id is part off a ssbond..
    inds <- ssdat$ss.bonds[,1]==atom.id

    if(any(inds)) {
      # Find ss-bond pair
      inds.paired <- ssdat$ss.bonds[which(inds), 2]

      # and change the spring force constant
      ks[inds.paired] <- k.ssbond
    }
  }
  return(ks)
}
```

The disulfide bridges can be supplied as input to **nma()** function via a simple two-column matrix:

```
# Define SS-bonds in a two-column matrix
ss.bonds <- matrix(c(76,94, 64,80, 30,115, 6,127),
                   ncol=2, byrow=TRUE)

# Calculate modes with custom force field
modes <- nma(pdb, pfc.fun=ff.custom, ss.bonds=ss.bonds)
```

Note that we can also use force field *calphax* to account for stronger interactions for beta bridges and helix 1-4 interactions:

```
# Use ff='calphax' to account for stronger beta-bridges and helix 1-4 interactions
sse <- dssp(pdb, resno=FALSE, full=TRUE)
modes <- nma(pdb, ff='calphax', ss.bonds=ss.bonds, sse=sse)
```

## Document Details

This document is shipped with the Bio3D package in both R and PDF formats. All code can be extracted and automatically executed to generate Figures and/or the PDF with the following commands:

```
library(rmarkdown)
render("Bio3D_nma.Rmd", "all")
```

## Information About the Current Bio3D Session

```
sessionInfo()
```

```
## R version 3.1.1 (2014-07-10)
## Platform: x86_64-redhat-linux-gnu (64-bit)
##
## locale:
##  [1] LC_CTYPE=en_US.UTF-8       LC_NUMERIC=C
##  [3] LC_TIME=en_US.UTF-8        LC_COLLATE=en_US.UTF-8
##  [5] LC_MONETARY=en_US.UTF-8    LC_MESSAGES=en_US.UTF-8
##  [7] LC_PAPER=en_US.UTF-8       LC_NAME=C
##  [9] LC_ADDRESS=C               LC_TELEPHONE=C
## [11] LC_MEASUREMENT=en_US.UTF-8 LC_IDENTIFICATION=C
##
## attached base packages:
## [1] parallel  grid      stats     graphics  grDevices utils     datasets
## [8] methods   base
##
## other attached packages:
## [1] bigmemory_4.4.6     bigmemory.sri_0.1.2 BH_1.54.0-2
## [4] lattice_0.20-29     rmarkdown_0.3.3     bio3d_2.1-0
##
## loaded via a namespace (and not attached):
## [1] codetools_0.2-8 digest_0.6.4    evaluate_0.5.5  formatR_0.10
## [5] htmltools_0.2.6 knitr_1.6       stringr_0.6.2   tools_3.1.1
## [9] yaml_2.1.13
```

## References

Fuglebakk, Edvin, Julián Echave, and Nathalie Reuter. 2012. "Measuring and comparing structural fluctuation patterns in large protein datasets." *Bioinformatics (Oxford, England)* 28 (19): 2431–40. doi:10.1093/bioinformatics/bts445.

Grant, B.J., A.P.D.C Rodrigues, K.M. Elsawy, A.J. Mccammon, and L.S.D. Caves. 2006. "Bio3d: An R Package for the Comparative Analysis of Protein Structures." *Bioinformatics* 22: 2695–96. doi:10.1093/bioinformatics/btl461.

Hinsen, K, A J Petrescu, S Dellerue, M C Bellissent-Funel, and G R Kneller. 2000. "Harmonicity in slow protein dynamics." *Chemical Physics* 261 (1-2): 25–37. doi:10.1016/S0301-0104(00)00222-6.

Romanowska, Julia, Krzysztof S. Nowinski, and Joanna Trylska. 2012. "Determining geometrically stable domains in molecular conformation sets." *Journal of Chemical Theory and Computation* 8 (8): 2588–99. doi:10.1021/ct300206j.

Tama, F, and Y H Sanejouand. 2001. "Conformational change of proteins arising from normal mode calculations." *Protein Eng* 14 (1): 1–6. doi:10.1093/protein/14.1.1.
