# Supplementary material for: Integrating protein structural dynamics and evolutionary analysis with Bio3D
Source: BMC Bioinformatics. 2014 Dec 10;15(1):399. doi: 10.1186/s12859-014-0399-6 (PMC4279791; doi:10.1186/s12859-014-0399-6)
Supplement: Additional file 2: — E. coli DHFR ensemble NMA and PCA, including a comparison of implemented similarity measures. [file 12859_2014_399_MOESM2_ESM.pdf]

# Ensemble NMA of *E.coli* DHFR structures

*Lars Skjaerven, Xin-Qiu Yao, Guido Scarabelli & Barry J. Grant*

*October 1, 2014*

This document provides **Additional File 2** for *Integrating protein structural dynamics and evolutionary analysis with Bio3D.*

## Background

Bio3D[1] is an R package that provides interactive tools for structural bioinformatics. The primary focus of Bio3D is the analysis of bimolecular structure, sequence and simulation data (Grant et al. 2006).

Normal mode analysis (NMA) is one of the major simulation techniques used to probe large-scale motions in biomolecules. Typical application is for the prediction of functional motions in proteins. Version 2.0 of the Bio3D package now includes extensive NMA facilities (see also the **NMA Vignette**). These include a unique collection of multiple elastic network model force-fields, automated ensemble analysis methods, and variance weighted NMA. Here we provide an in-depth demonstration of ensemble NMA with working code that comprise complete executable examples[2].

### Requirements

Detailed instructions for obtaining and installing the Bio3D package on various platforms can be found in the **Installing Bio3D Vignette** available both on-line and from within the Bio3D package. In addition to Bio3D the *MUSCLE* multiple sequence alignment program (available from the muscle home page must be installed on your system and in the search path for executables. Please see the installation vignette for further details.

### About this document

This vignette was generated using **Bio3D version 2.1.0**.

## 1 Part I: Ensemble NMA of *E.coli* DHFR structures

In this vignette we perform *ensemble NMA* on the complete collection of *E.coli* Dihydrofolate reductase (DHFR) structures in the protein data-bank (PDB). Starting from only one PDB identifier (PDB ID 1rx2) we show how to search the PDB for related structures using BLAST, fetch and align the structures, and finally calculate the normal modes of each individual structure in order to probe for potential differences in structural flexibility.

[1] The latest version of the package, full documentation and further vignettes (including detailed installation instructions) can be obtained from the main Bio3D website: http://thegrantlab.org/bio3d/

[2] This vignette contains executable examples, see `help(vignette)` for further details.

## 1.1 Search and retrieve DHFR structures

Below we perform a blast search of the PDB database to identify related structures to our query *E.coli* DHFR sequence. In this particular example we use function **get.seq()** to fetch the query sequence for chain A of the PDB ID 1RX2 and use this as input to **blast.pdb()**. Note that **get.seq()** would also allow the corresponding UniProt identifier.

```
library(bio3d)
```

```
aa <- get.seq("1rx2_A")
blast <- blast.pdb(aa)
```

```
##  Searching ... please wait (updates every 5 seconds) RID = 2N0CZ05H01R
##  ..........................................................................................
##  Reporting 548 hits
```

Function **plot.blast()** facilitates the visualization and filtering of the Blast results. It will attempt to set a seed position to the point of largest drop-off in normalized scores (i.e. the biggest jump in E-values). In this particular case we specify a cutoff (after initial plotting) of 225 to include only the relevant *E.coli* structures:

```
hits <- plot(blast, cutoff=225)
```

```
##   * Possible cutoff values:    250 22
##             Yielding Nhits:    101 548
##
##   * Chosen cutoff value of:    225
##             Yielding Nhits:    101
```

The Blast search and subsequent filtering identified a total of 101 related PDB structures to our query sequence. The PDB identifiers of this collection are accessible through the `pdb.id` attribute to the `hits` object (`hits$pdb.id`). Note that adjusting the cutoff argument (to **plot.blast()**) will result in a decrease or increase of hits.

We can now use function **get.pdb()** and **pdbslit()** to fetch and parse the identified structures. Finally, we use **pdbaln()** to align the PDB structures.

```
# fetch PDBs
raw.files <- get.pdb(hits$pdb.id, path = "raw_pdbs", gzip=TRUE)
```

```
# split by chain ID
files <- pdbsplit(raw.files, ids = hits$pdb.id, path = "raw_pdbs/split_chain", ncore=4)
```

```
# align structures
pdbs.all <- pdbaln(files, fit=TRUE)
```

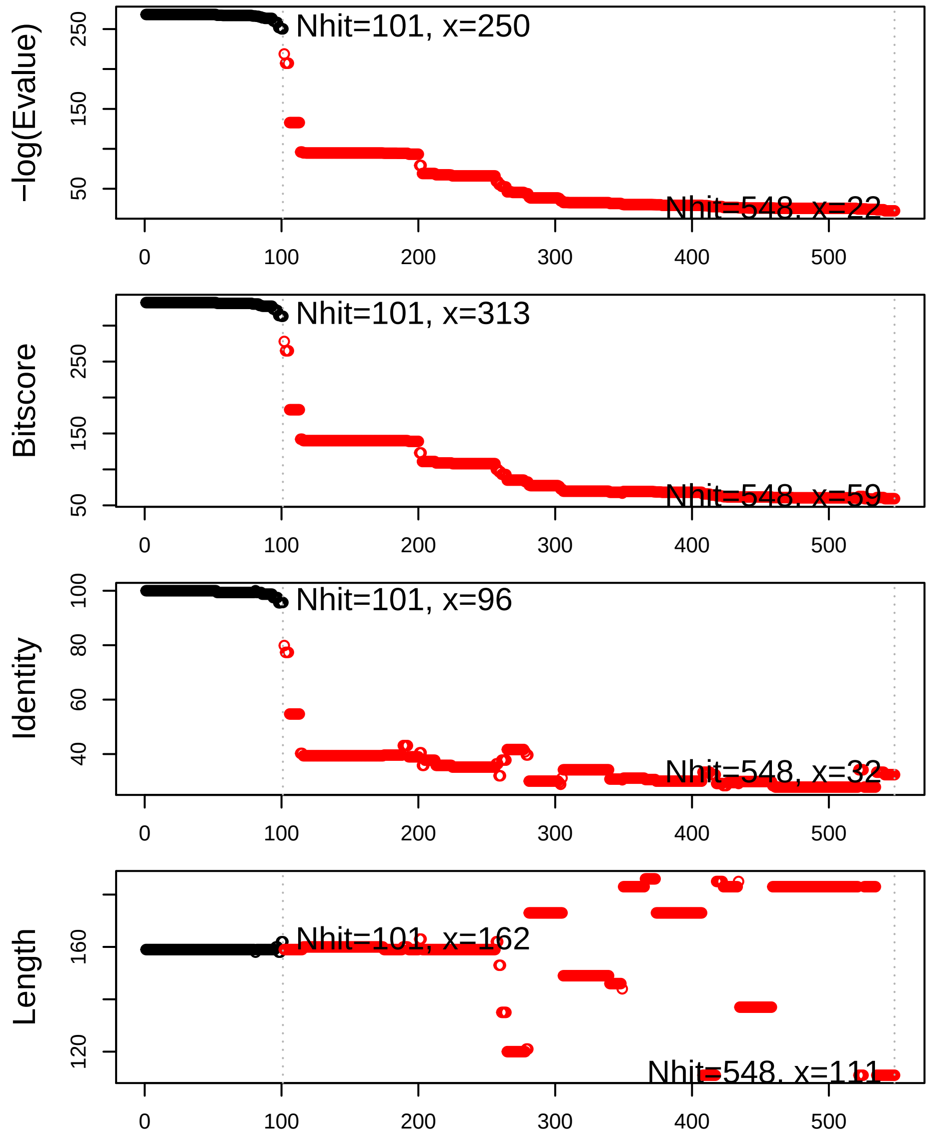


Figure 1: Blast results. Visualize and filter blast results through function **plot.blast()**. Here we proceed with only the top scoring hits (black).

The *pdbs.all* object now contains *aligned* C-alpha atom data, including Cartesian coordinates, residue numbers, residue types, and B-factors. The sequence alignment is also stored by default to the FASTA format file 'aln.fa' (to view this you can use an alignment viewer such as SEAVIEW, see *Requirements* section above). In cases where manual edits of the alignment are necessary you can re-read the sequence alignment and coordinate data with:

```
aln <- read.fasta('aln.fa')
pdbs.all <- read.fasta.pdb(aln)
```

At this point the *pdbs.all* object contains all identified 101 structures. This might include structures with missing residues, and/or multiple structurally redundant conformers. For our subsequent NMA missing in-structure residues might bias the calculation, and redundant structures can be useful to omit to reduce the computational load. Below we inspect the connectivity of the PDB structures with a function call to **inspect.connectivity()**, and **pdbs.filter()** to filter out those structures from our *pdbs.all* object. Similarly, we omit structures that are conformationally redundant to reduce the computational load with function **rmsd.filter()**:

```
# remove structures with missing residues
conn <- inspect.connectivity(pdbs.all, cut=4.05)
pdbs <- pdbs.filter(pdbs.all, row.inds=which(conn))

# which structures are omitted
which(!conn)

# remove conformational redundant structures
rd <- rmsd.filter(pdbs$xyz, cutoff=0.1, fit=TRUE)
pdbs <- pdbs.filter(pdbs, row.inds=rd$ind)

# remove "humanized" e-coli dhfr
excl <- unlist(lapply(c("3QL0", "4GH8"), grep, pdbs$id))
pdbs <- pdbs.filter(pdbs, row.inds=which(!(1:length(pdbs$id) %in% excl)))

# a list of PDB codes of our final selection
ids <- unlist(strsplit(basename(pdbs$id), split=".pdb"))
```

Use **print()** to see a short summary of the pdbs object:

```
print(pdbs, alignment=FALSE)
```

```
##
## Call:
##   pdbs.filter(pdbs = pdbs, row.inds = which(!(1:length(pdbs$id) %in%
##     excl)))
##
## Class:
##   pdbs, fasta
```

```
##
## Alignment dimensions:
##   82 sequence rows; 159 position columns (159 non-gap, 0 gap)
##
## + attr: id, xyz, resno, b, chain, ali, resid, call
```

### 1.2 Annotate collected PDB structures

Function **pdb.annotate()** provides a convenient way of annotating the PDB files we have collected. Below we use the function to annotate each structure to its source species. This will come in handy when annotating plots later on:

```
anno <- pdb.annotate(ids)
print(unique(anno$source))
```

```
## [1] "Escherichia coli"      "Escherichia coli K12"  "Escherichia coli K-12"
```

### 1.3 Principal component analysis

A principal component analysis (PCA) can be performed on the structural ensemble (stored in the *pdbs* object) with function **pca.xyz()**. To obtain meaningful results we first superimpose all structures on the *invariant core* (function **core.find()**).

```
# find invariant core
core <- core.find(pdbs)

# superimpose all structures to core
pdbs$xyz = pdbfit(pdbs, core$c0.5A.xyz)

# identify gaps, and perform PCA
gaps.pos <- gap.inspect(pdbs$xyz)
gaps.res <- gap.inspect(pdbs$ali)
pc.xray <- pca.xyz(pdbs$xyz[,gaps.pos$f.inds])
```

```
# plot PCA
plot(pc.xray)
```

Note that a call to the wrapper function **pca()** would provide identical results as the above code:

```
pc.xray <- pca(pdbs, core.find=TRUE)
```

Function **rmsd()** will calculate all pairwise RMSD values of the structural ensemble. This facilitates clustering analysis based on the pairwise structural deviation:

```
rd <- rmsd(pdbs)
hc.rd <- hclust(dist(rd))
grps.rd <- cutree(hc.rd, k=4)
```

Projection of the X-ray conformers on to their two largest eigenvectors shows that the E.coli DHFR structures can be divided into three major groups: closed, open, and occluded conformations:

```
plot(pc.xray$z[,1:2], col="grey50", pch=16, cex=1.3,
     ylab="Prinipcal Component 2", xlab="Principal Component 1")
points(pc.xray$z[,1:2], col=grps.rd, pch=16, cex=0.9)
```

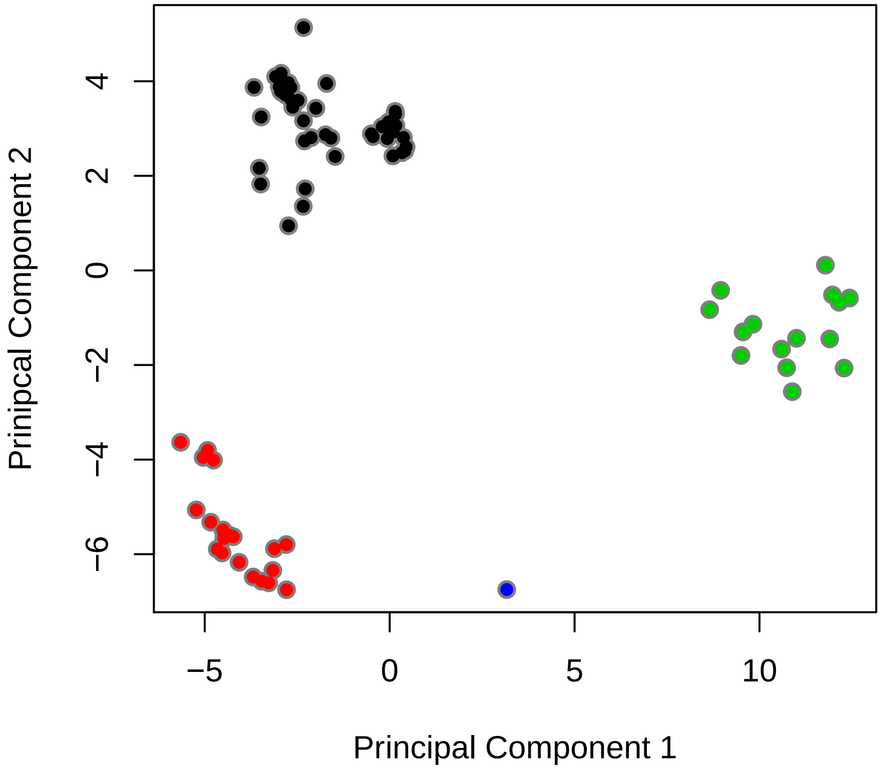


Figure 2: Projection of MD conformers onto the X-ray PC space reveals that the E.coli DHFR structures can be divided into three major groups along their two first eigenvectors. Each dot is colored according to their cluster membership: occluded conformations (green), open conformations (black), and closed conformations (red).

To visualize the major structural variations in the ensemble function **mktrj()** can be used to generate a trajectory PDB file by interpolating along the eigenvector:

```
mktrj(pc.xray, pc=1,
      resno=pdbs$resno[1, gaps.res$f.inds],
      resid=pdbs$resid[1, gaps.res$f.inds])
```

Function **pdbfit()** can be used to write the PDB files to separate directories according to their cluster membership:

```r
pdbfit(pdbs.filter(pdbs, row.inds=which(grps.rd==1)), outpath="grps1") ## closed
pdbfit(pdbs.filter(pdbs, row.inds=which(grps.rd==2)), outpath="grps2") ## open
pdbfit(pdbs.filter(pdbs, row.inds=which(grps.rd==3)), outpath="grps3") ## occluded
```

### 1.4 Normal modes analysis

Function **nma.pdbs()** will calculate the normal modes of each protein structure stored in the *pdbs* object. The normal modes are calculated on the full structures as provided by object *pdbs*. With the default argument `rm.gaps=TRUE` unaligned atoms are omitted from output:

```r
modes <- nma.pdbs(pdbs, rm.gaps=TRUE, ncore=4)
```

The *modes* object of class *enma* contains aligned normal mode data including fluctuations, RMSIP data, and aligned eigenvectors. A short summary of the *modes* object can be obtain by calling the function **print()**, and the aligned fluctuations can be plotted with function **plot.enma()**. This function also facilitates the calculation and visualization of sequence conservation (use argument `conservation=TRUE`), and fluctuation variance (use argument `variance=TRUE`).

```r
print(modes)
```

```
##
## Call:
##   nma.pdbs(pdbs = pdbs, rm.gaps = TRUE, ncore = 4)
##
## Class:
##   enma
##
## Number of structures:
##   82
##
## Attributes stored:
##   - Root mean square inner product (RMSIP)
##   - Aligned atomic fluctuations
##   - Aligned eigenvectors (gaps removed)
##   - Dimensions of x$U.subspace: 477x471x82
##
## Coordinates were aligned prior to NMA calculations
##
## + attr: fluctuations, rmsip, U.subspace, L, full.nma, xyz,
##         call
```

```r
# plot modes fluctuations
plot(modes, pdbs=pdbs, col=grps.rd, variance=TRUE)
```

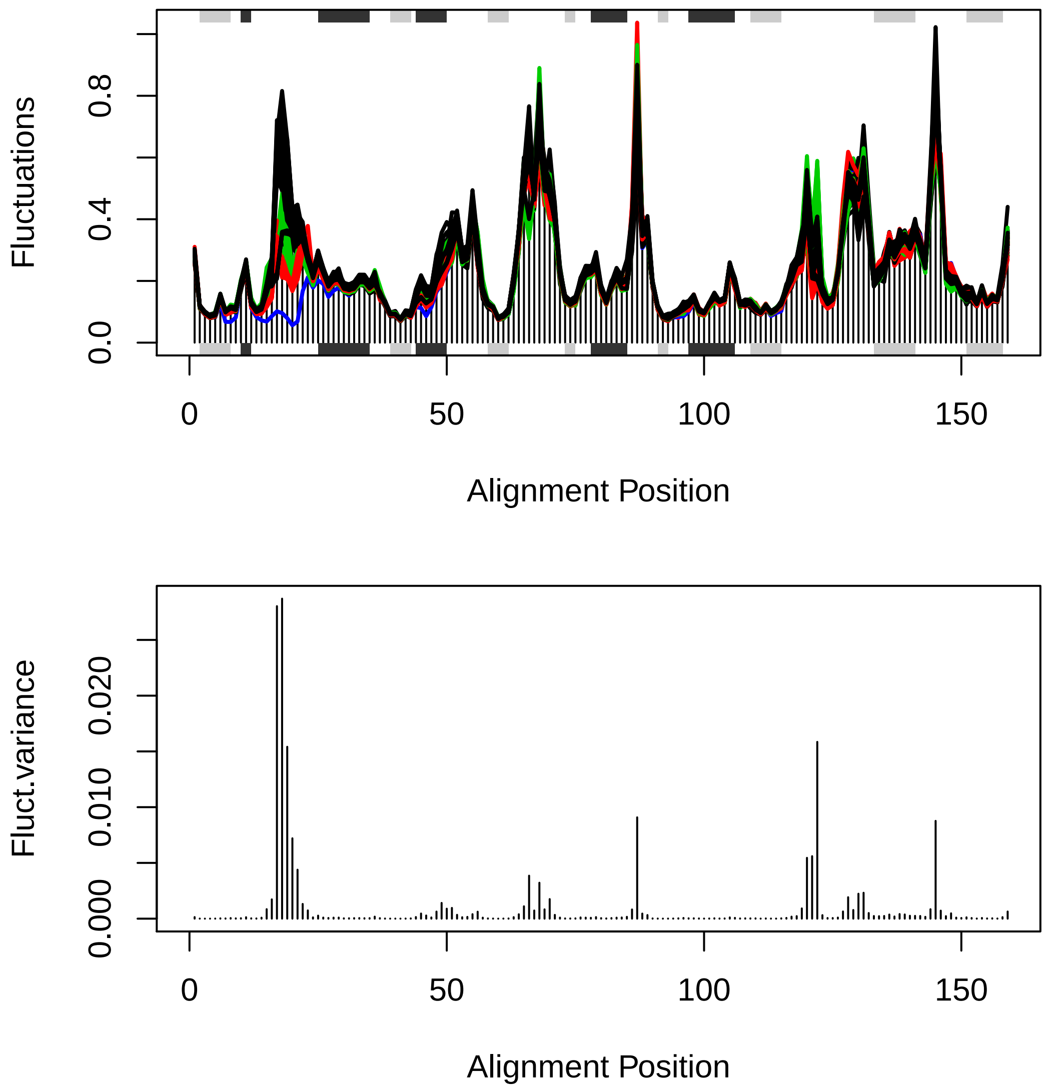


Figure 3: Normal mode fluctuations of the E.coli DHFR ensemble. (**upper panel**) Each line represent the mode fluctuations for the individual structures. Color coding according to structural clustering: green (occluded), black (open), and red (closed). (**lower panel**) Variance of mode fluctuations. The Met20 loop seems to display the largest deviations / differences in predicted fluctuations.

```
hc.nma <- hclust(as.dist(1-modes$rmsip))
grps.nma <- cutree(hc.nma, k=4)
```

```
heatmap(1-modes$rmsip, distfun = as.dist, labRow = ids, labCol = ids,
        ColSideColors=as.character(grps.nma), RowSideColors=as.character(grps.nma))
```

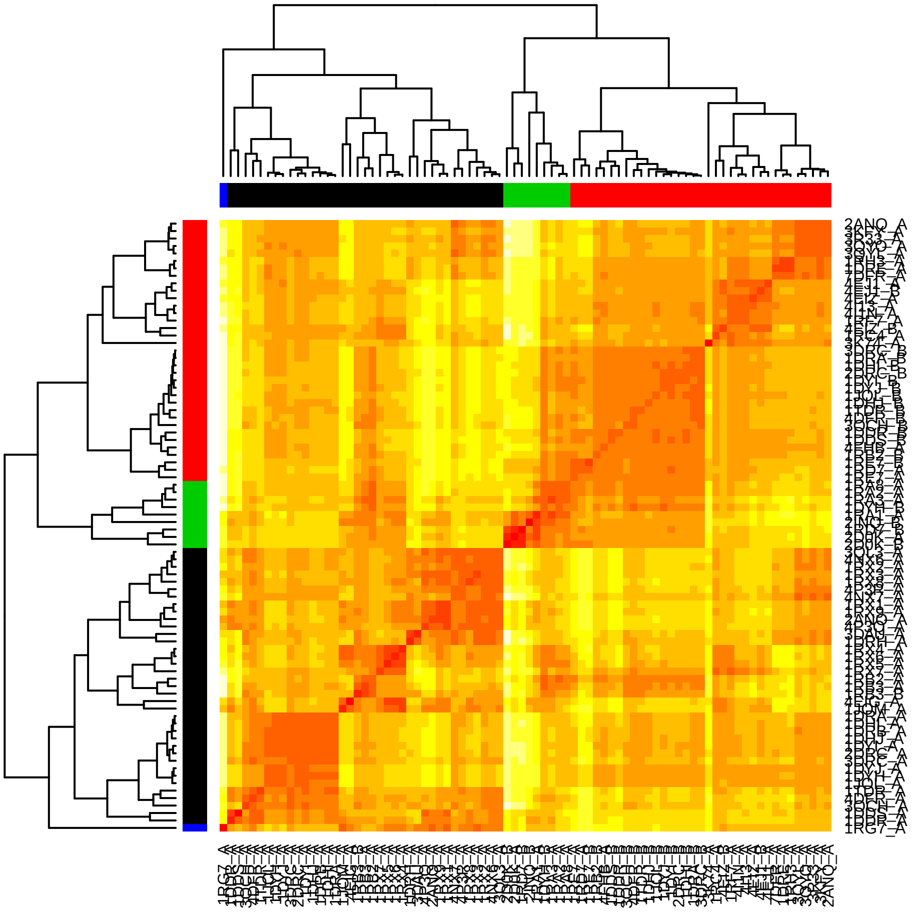

Figure 4: Clustering of structures based on their normal modes similarity (in terms of pair-wise RMSIP values).

## 1.5 Fluctuation analysis

Comparing the mode fluctuations of two groups of structures can reveal specific regions of distinct flexibility patterns. Below we focus on the differences between the open (black), closed (red) and occluded (green) conformations of the *E.coli* structures:

```
cols <- grps.rd
cols[which(cols!=1 & cols!=2)]=NA
plot(modes, pdbs=pdbs, col=cols, signif=TRUE)
```

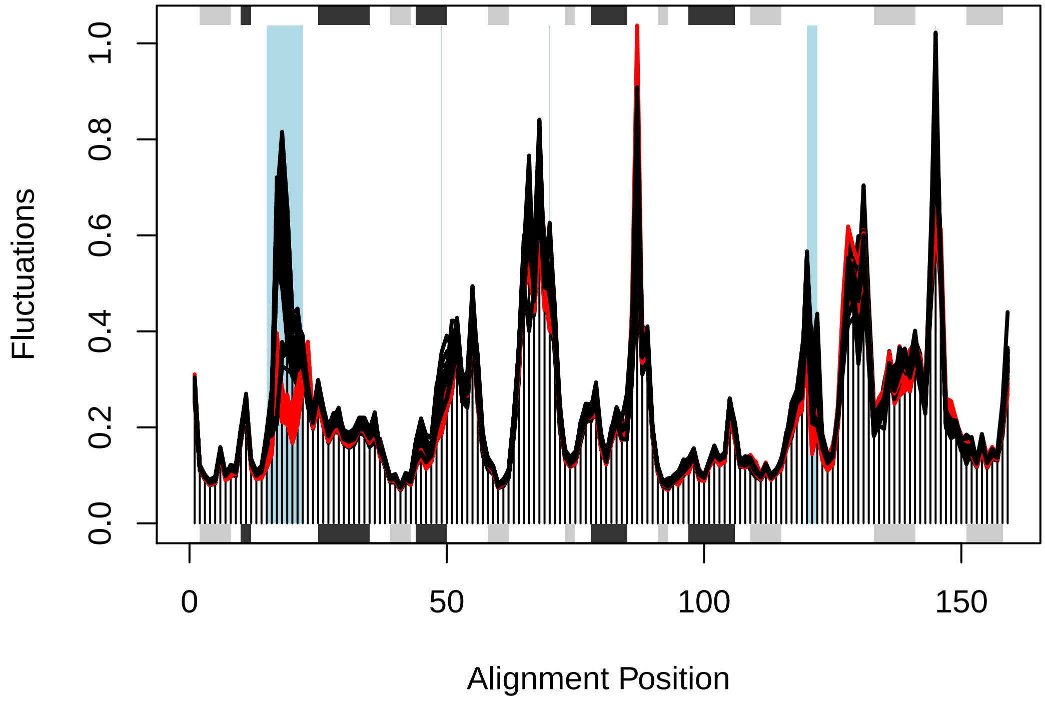


Figure 5: Comparison of mode fluctuations between open (black) and closed (red) conformers. Significant differences among the mode fluctuations between the two groups are marked with shaded blue regions.

```
cols <- grps.rd
cols[which(cols!=1 & cols!=3)]=NA
plot(modes, pdbs=pdbs, col=cols, signif=TRUE)
```

```
cols <- grps.rd
cols[which(grps.rd!=2 & grps.rd!=3)]=NA
plot(modes, pdbs=pdbs, col=cols, signif=TRUE)
```

```
##    PDB has ALT records, taking A only, rm.alt=TRUE
```

### 1.6 Compare with MD simulation

The above analysis can also nicely be integrated with molecular dynamics (MD) simulations. Below we read in a 5 ns long MD trajectory (of PDB ID 1RX2). We visualize the conformational sampling by projecting the MD conformers onto the principal components (PCs) of the X-ray ensemble, and finally compare the PCs of the MD simulation to the normal modes:

```
pdb <- read.pdb("md-traj/1rx2-CA.pdb")
trj <- read.ncdf("md-traj/1rx2_5ns.nc")
```

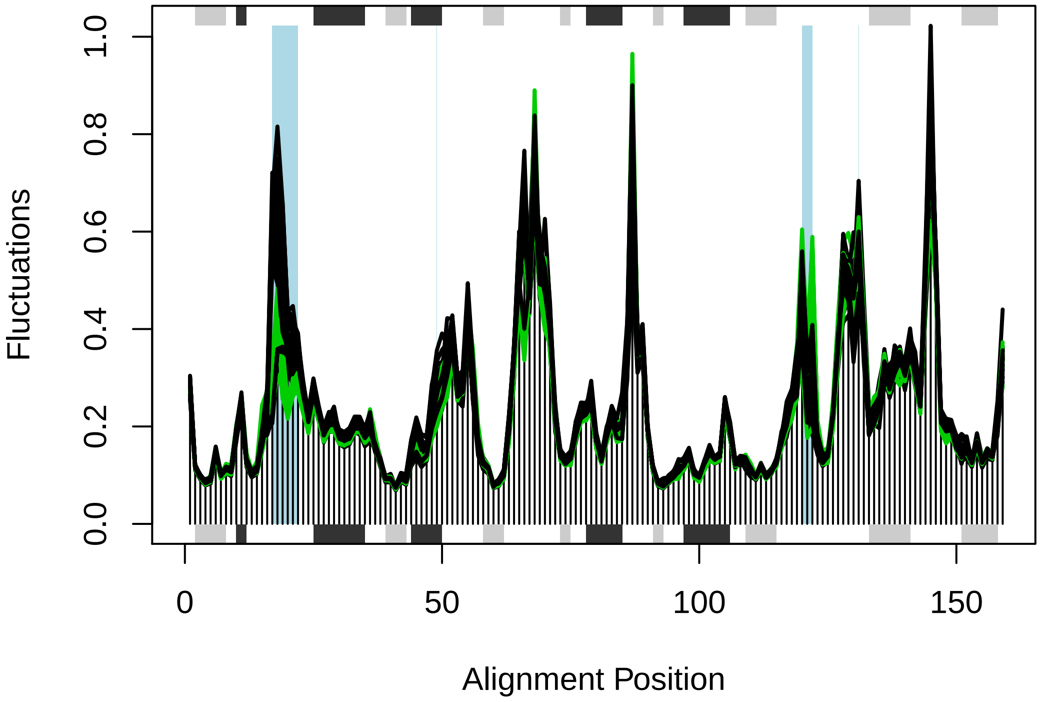


Figure 6: Comparison of mode fluctuations between open (black) and occluded (green) conformers. Significant differences among the mode fluctuations between the two groups are marked with shaded blue regions.

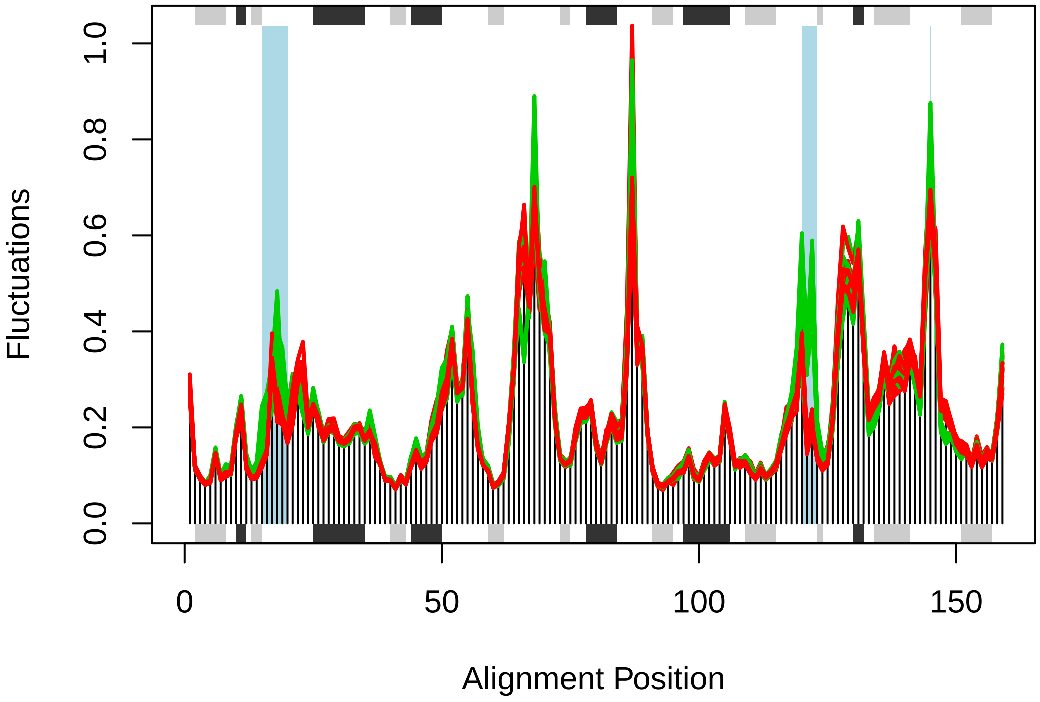


Figure 7: Comparison of mode fluctuations between closed (red) and occluded (green) conformers. Significant differences among the mode fluctuations between the two groups are marked with shaded blue regions.

```r
md.inds <- pdb2aln.ind(aln=pdbs, pdb=pdb, id="md", inds=gaps.res$f.inds)
trj <- trj[, atom2xyz(md.inds)]
trj <- fit.xyz(fixed=pdbs$xyz[1, ],  mobile=trj,
               fixed.inds=core$c0.5A.xyz, mobile.inds=core$c0.5A.xyz)

proj <- project.pca(trj, pc.xray)
cols <- densCols(proj[,1:2])
```

```r
plot(proj[,1:2], col=cols, pch=16,
     ylab="Prinipcal Component 2", xlab="Principal Component 1",
     xlim=range(pc.xray$z[,1]), ylim=range(pc.xray$z[,2]))
points(pc.xray$z[,1:2], col=1, pch=1, cex=1.1)
points(pc.xray$z[,1:2], col=grps.rd, pch=16)
```

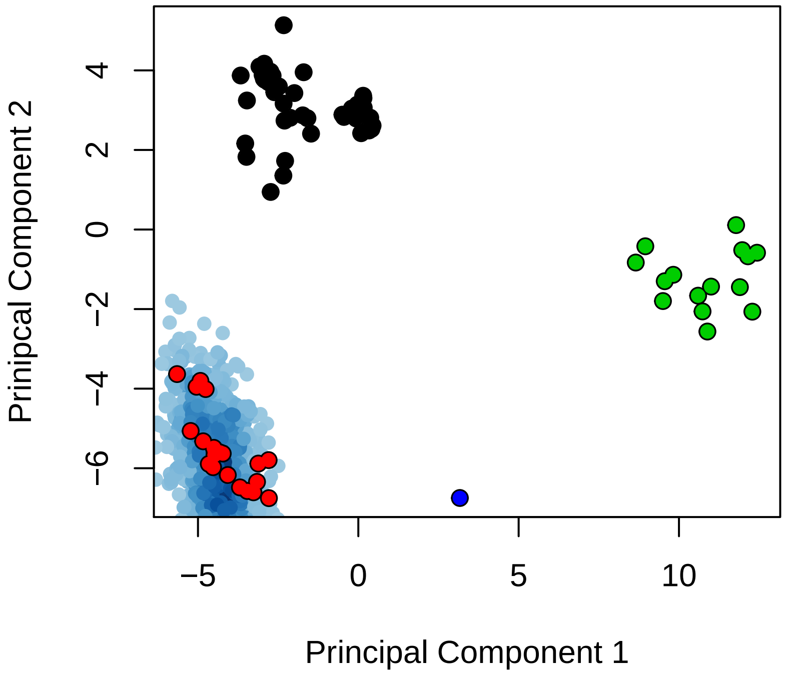


Figure 8: Projection of MD conformers onto the X-ray PC space provides a two dimensional representation of the conformational sampling along the MD simulation (blue dots).

```r
# PCA of the MD trajectory
pc.md <- pca.xyz(trj)

# compare MD-PCA and NMA
r <- rmsip(pc.md$U, modes$U.subspace[,,1])

print(r)
```

```
## $overlap
```

```
##         b1    b2    b3    b4    b5    b6    b7    b8    b9   b10
## a1  0.363 0.022 0.002 0.036 0.016 0.001 0.003 0.009 0.000 0.024
## a2  0.176 0.236 0.004 0.000 0.089 0.011 0.004 0.000 0.001 0.005
## a3  0.105 0.002 0.021 0.001 0.000 0.016 0.003 0.001 0.016 0.084
## a4  0.003 0.094 0.157 0.116 0.067 0.016 0.045 0.045 0.029 0.000
## a5  0.025 0.038 0.000 0.016 0.003 0.022 0.018 0.048 0.014 0.000
## a6  0.053 0.015 0.044 0.053 0.056 0.077 0.034 0.112 0.005 0.007
## a7  0.000 0.080 0.142 0.009 0.002 0.071 0.008 0.003 0.066 0.001
## a8  0.030 0.228 0.066 0.331 0.013 0.024 0.000 0.001 0.010 0.001
## a9  0.015 0.001 0.032 0.008 0.190 0.003 0.004 0.038 0.011 0.002
## a10 0.002 0.034 0.137 0.000 0.027 0.001 0.067 0.018 0.005 0.004
##
## $rmsip
## [1] 0.6441
##
## attr(,"class")
## [1] "rmsip"
```

```
plot(r, xlab="MD PCA", ylab="NMA")
```

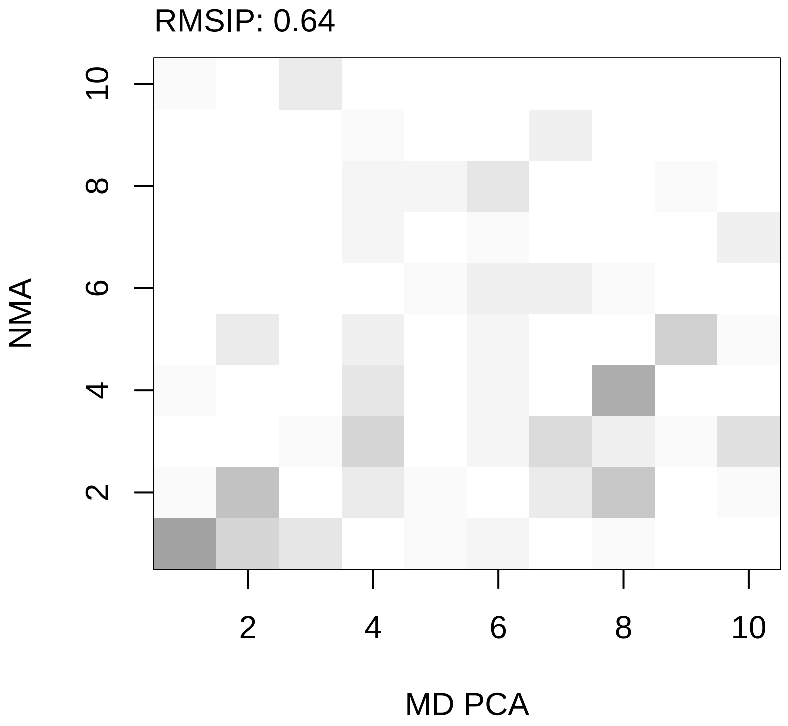


Figure 9: Overlap map between normal modes and principal components of a 5 ns long MD simulation. The two subsets yields an RMSIP value of 0.64, where a value of 1 would idicate identical directionality.

```r
# compare MD-PCA and X-rayPCA
r <- rmsip(pc.md, pc.xray)
```

### 1.7 Domain analysis with GeoStaS

Identification of regions in the protein that move as rigid bodies is facilitated with the implementation of the GeoStaS algorithm (Romanowska, Nowinski, and Trylska 2012). Below we demonstrate the use of function **geostas()** on data obtained from ensemble NMA, an ensemble of PDB structures, and a 5 ns long MD simulation. See `help(geostas)` for more details and further examples.

**GeoStaS on a PDB ensemble**: Below we input the `pdbs` object to function **geostas()** to identify dynamic domains. Here, we attempt to divide the structure into 2 sub-domains using argument `k=2`. Function **geostas()** will return a `grps` attribute which corresponds to the domain assignment for each C-alpha atom in the structure. Note that we use argument `fit=FALSE` to avoid re-fitting the coordinates since. Recall that the coordinates of the `pdbs` object has already been superimposed to the identified invariant core (see above). To visualize the domain assignment we write a PDB trajectory of the first principal component (of the Cartesian coordinates of the `pdbs` object), and add argument `chain=gs.xray$grps` to replace the chain identifiers with the domain assignment:

```r
# Identify dynamic domains
gs.xray <- geostas(pdbs, k=2, fit=FALSE)

# Visualize PCs with colored domains (chain ID)
mktrj(pc.xray, pc=1, chain=gs.xray$grps)
```

**GoeStaS on ensemble NMA**: We can also identify dynamic domains from the normal modes of the ensemble of 82 protein structures stored in the `modes` object. By using function **mktrj.enma()** we generate a trajectory from the first five modes for all 82 structures. We then input this trajectory to function **geostas()**.

```r
# Build conformational ensemble
trj.nma <- mktrj.enma(modes, pdbs, m.inds=1:5, s.inds=NULL, mag=10, step=2, rock=FALSE)

trj.nma
```

```
##
##    Total Frames#: 4510
##    Total XYZs#:   477,  (Atoms#:  159)
##
##     [1]  25.125  59.135  5.302  <...>  33.181  46.72  7.348  [2151270]
##
## + attr: Matrix DIM = 4510 x 477
```

```r
# Fit to invariant core
trj.nma <- fit.xyz(trj.nma[1,], trj.nma,
                   fixed.inds=core$c0.5A.xyz,
```

```
                    mobile.inds=core$c0.5A.xyz)

# Run geostas to find domains
gs.nma <- geostas(trj.nma, k=2, fit=FALSE)
```

```
# Write NMA generated trajectory with domain assignment
write.pdb(xyz=trj.nma, chain=gs.nma$grps)
```

**GeoStaS on a MD trajectory**: The domain analysis can also be performed on the trajectory data obtained from the MD simulation (see above). Recall that the MD trajectory has already been superimposed to the invariant core. We therefore use argument `fit=FALSE` below. We then perform a new PCA of the MD trajectory, and visualize the domain assingments with function **mktrj()**:

```
gs.md <- geostas(trj, k=2, fit=FALSE)
pc.md <- pca(trj, fit=FALSE)
mktrj(pc.md, pc=1, chain=gs.md$grps)
```

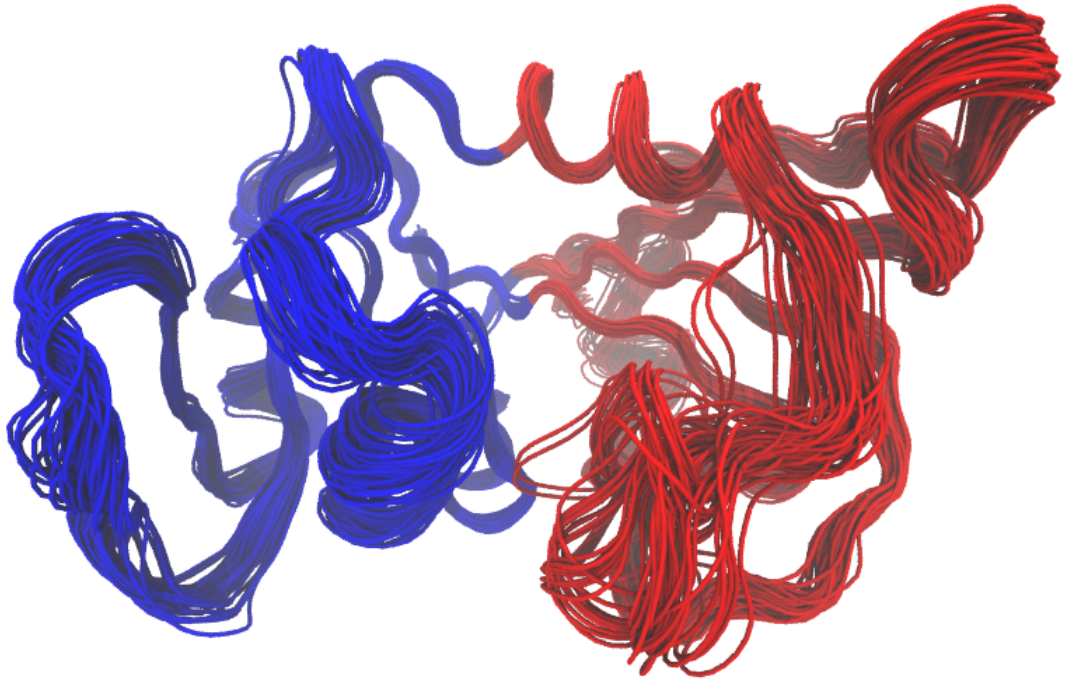

Figure 10: Visualization of domain assignment using function **geostas()** on the first five normal modes of the entire ensemble of 82 DHFR structures.

## 1.8 Measures for modes comparison

Bio3D now includes multiple measures for the assessment of similarity between two normal mode objects. This enables clustering of related proteins based on the predicted modes of motion. Below

we demonstrate the use of root mean squared inner product (RMSIP), squared inner product (SIP), covariance overlap, bhattacharyya coefficient, and PCA of the corresponding covariance matrices.

```
# Similarity of atomic fluctuations
sip <- sip(modes)
hc.sip <- hclust(as.dist(1-sip), method="ward.D2")
grps.sip <- cutree(hc.sip, k=3)
```

```
hclustplot(hc.sip, k=3, colors=grps.rd, labels=ids, cex=0.7, main="SIP", fillbox=FALSE)

par(fig=c(.65, 1, .45, 1), new = TRUE)
plot(pc.xray$z[,1:2], col="grey50", pch=16, cex=1.1,
     ylab="", xlab="", axes=FALSE, bg="red")
points(pc.xray$z[,1:2], col=grps.sip, pch=16, cex=0.7)
box()
```

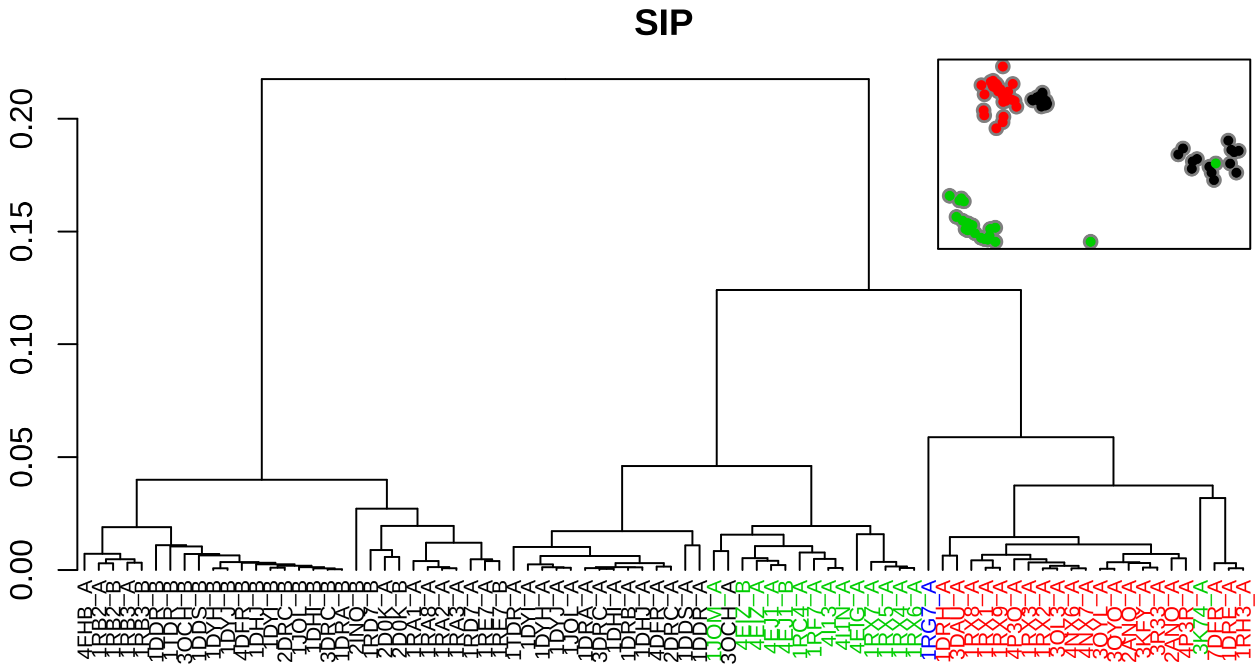


Figure 11: Dendrogram shows the results of hierarchical clustering of structures based on the similarity of atomic fluctuations calculated from NMA. Colors of the labels depict associated conformatial state: green (occluded), black (open), and red (closed). The inset shows the conformerplot (see Figure 2), with colors according to clustering based on pairwise SIP values.

```
# RMSIP
rmsip <- rmsip(modes)
hc.rmsip <- hclust(dist(1-rmsip), method="ward.D2")
grps.rmsip <- cutree(hc.rmsip, k=3)
```

```
hclustplot(hc.rmsip, k=3, colors=grps.rd, labels=ids, cex=0.7, main="RMSIP", fillbox=FALSE)

par(fig=c(.65, 1, .45, 1), new = TRUE)
plot(pc.xray$z[,1:2], col="grey50", pch=16, cex=1.1,
     ylab="", xlab="", axes=FALSE)
points(pc.xray$z[,1:2], col=grps.rmsip, pch=16, cex=0.7)
box()
```

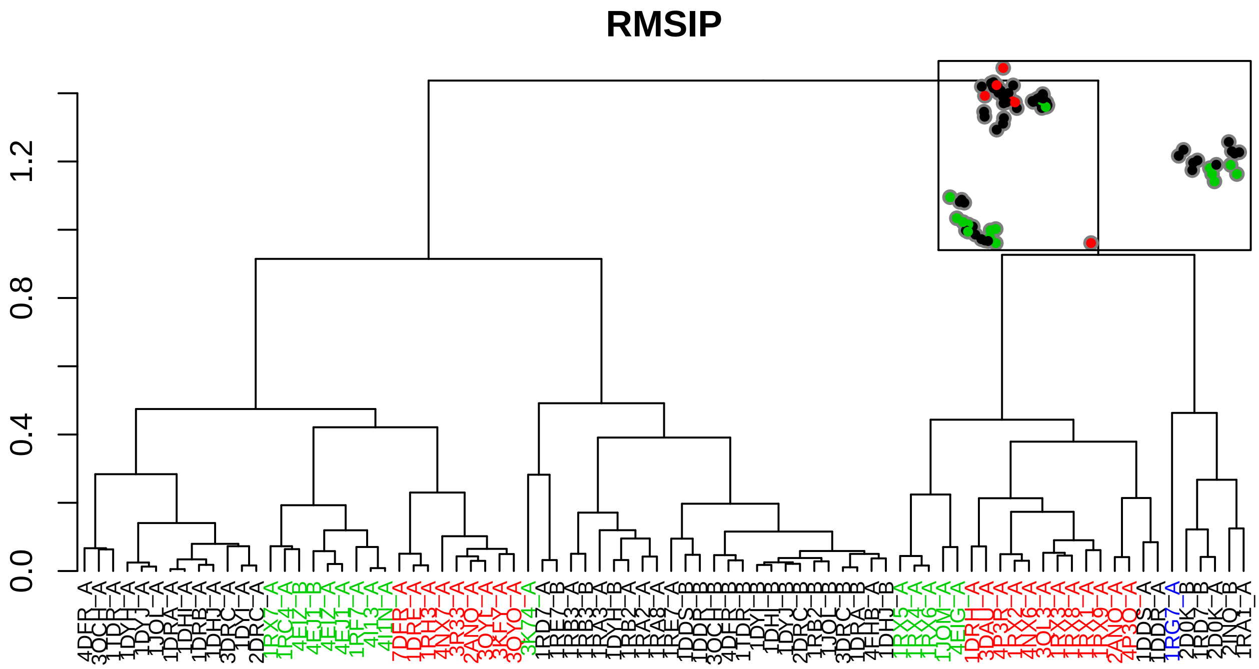


Figure 12: Dendrogram shows the results of hierarchical clustering of structures based on their pairwise RMSIP values (calculated from NMA). Colors of the labels depict associated conformatial state: green (occluded), black (open), and red (closed). The inset shows theconformerplot (see Figure 2), with colors according to clustering based on the pairwise RMSIP values.

```
# Covariance overlap
co <- covsoverlap(modes, subset=200)
hc.co <- hclust(as.dist(1-co), method="ward.D2")
grps.co <- cutree(hc.co, k=3)
```

```
hclustplot(hc.co, k=3, colors=grps.rd, labels=ids, cex=0.7, main="Covariance overlap", fillbox=

par(fig=c(.65, 1, .45, 1), new = TRUE)
plot(pc.xray$z[,1:2], col="grey50", pch=16, cex=1.1,
     ylab="", xlab="", axes=FALSE)
points(pc.xray$z[,1:2], col=grps.co, pch=16, cex=0.7)
box()
```

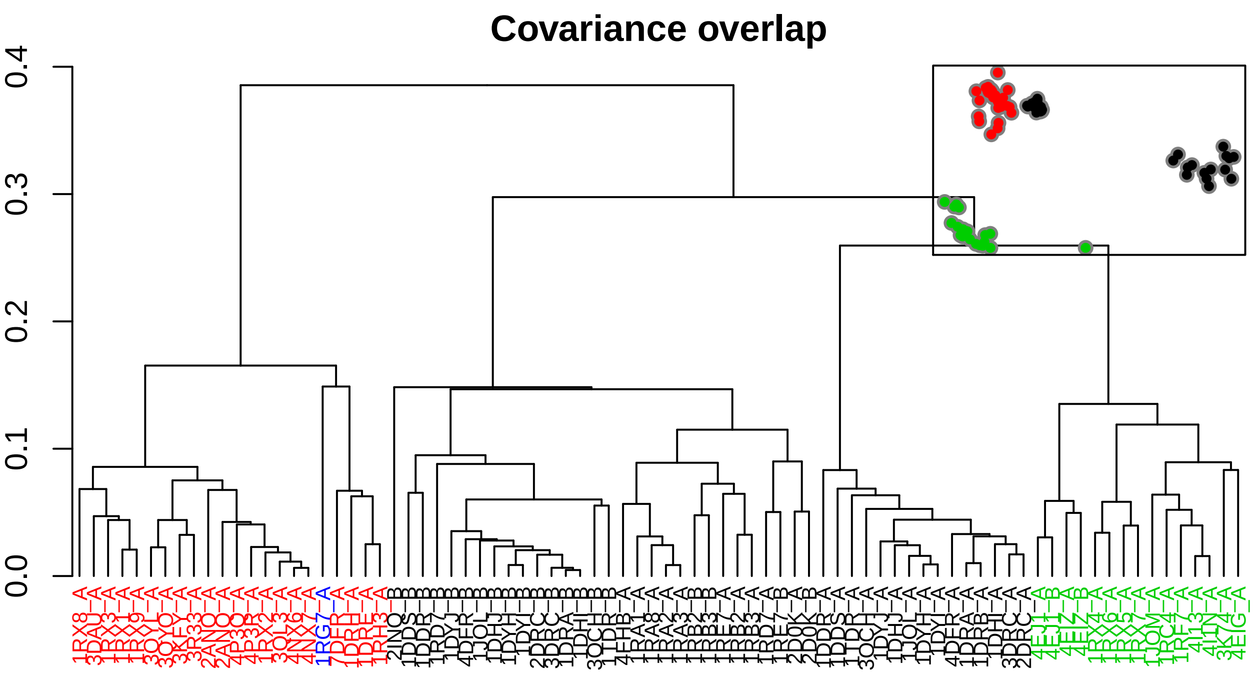


Figure 13: Dendrogram shows the results of hierarchical clustering of structures based on their pairwise covariance overlap (calculated from NMA). Colors of the labels depict associated conformatial state: green (occluded), black (open), and red (closed). The inset shows the conformerplot (see Figure 2), with colors according to clustering of the Covariance overlap measure.

```
# Bhattacharyya coefficient
covs <- cov.enma(modes)
bc <- bhattacharyya(modes, covs=covs)
hc.bc <- hclust(dist(1-bc), method="ward.D2")
grps.bc <- cutree(hc.bc, k=3)
```

```
hclustplot(hc.bc, k=3, colors=grps.rd, labels=ids, cex=0.7, main="Bhattacharyya coefficient", 

par(fig=c(.65, 1, .45, 1), new = TRUE)
plot(pc.xray$z[,1:2], col="grey50", pch=16, cex=1.1,
     ylab="", xlab="", axes=FALSE)
points(pc.xray$z[,1:2], col=grps.bc, pch=16, cex=0.7)
box()
```

```
# PCA of covariance matrices
pc.covs <- pca.array(covs)
hc.covs <- hclust(dist(pc.covs$z[,1:2]), method="ward.D2")
grps.covs <- cutree(hc.covs, k=3)
```

```
hclustplot(hc.covs, k=3, colors=grps.rd, labels=ids, cex=0.7, main="PCA of covariance matrices
```

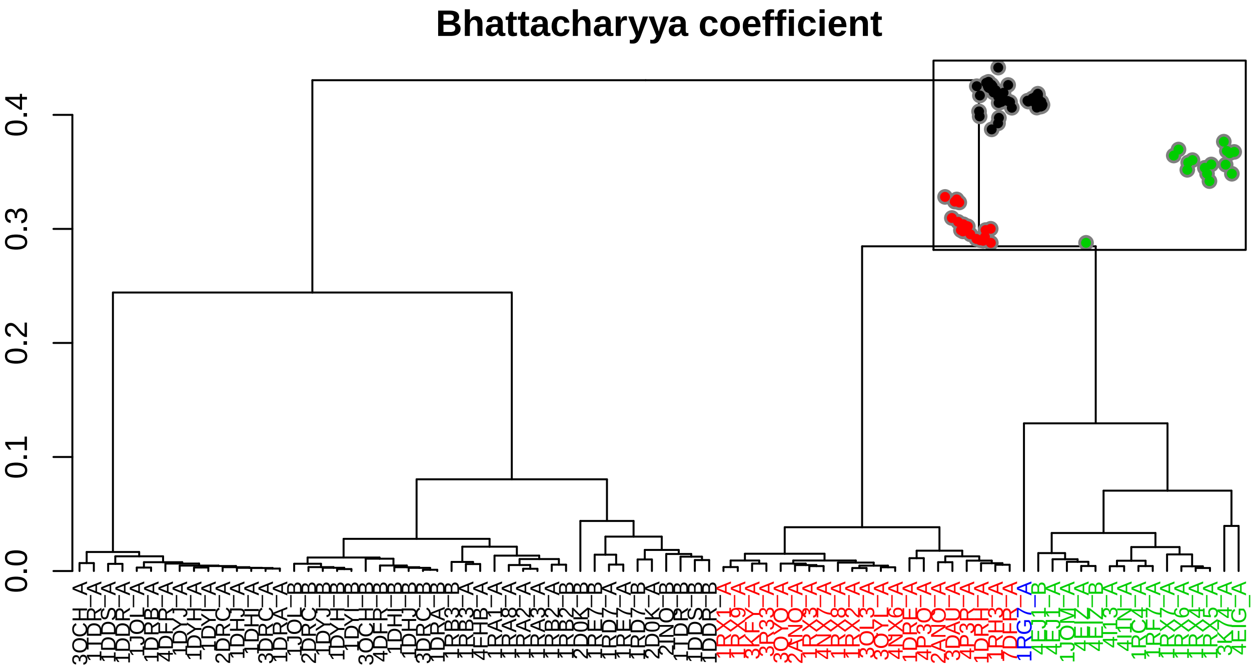


Figure 14: Dendrogram shows the results of hierarchical clustering of structures based on their pairwise Bhattacharyya coefficient (calculated from NMA). Colors of the labels depict associated conformatial state: green (occluded), black (open), and red (closed). The inset shows the conformerplot (see Figure 2), with colors according to clustering of the pairwise Bhattacharyya coefficients.

```
par(fig=c(.65, 1, .45, 1), new = TRUE)
plot(pc.xray$z[,1:2], col="grey50", pch=16, cex=1.1,
     ylab="", xlab="", axes=FALSE)
points(pc.xray$z[,1:2], col=grps.covs, pch=16, cex=0.7)
box()
```

## Document Details

This document is shipped with the Bio3D package in both R and PDF formats. All code can be extracted and automatically executed to generate Figures and/or the PDF with the following commands:

```
library(rmarkdown)
render("Bio3D_nma-dhfr-partI.Rmd", "all")
```

## Information About the Current Bio3D Session

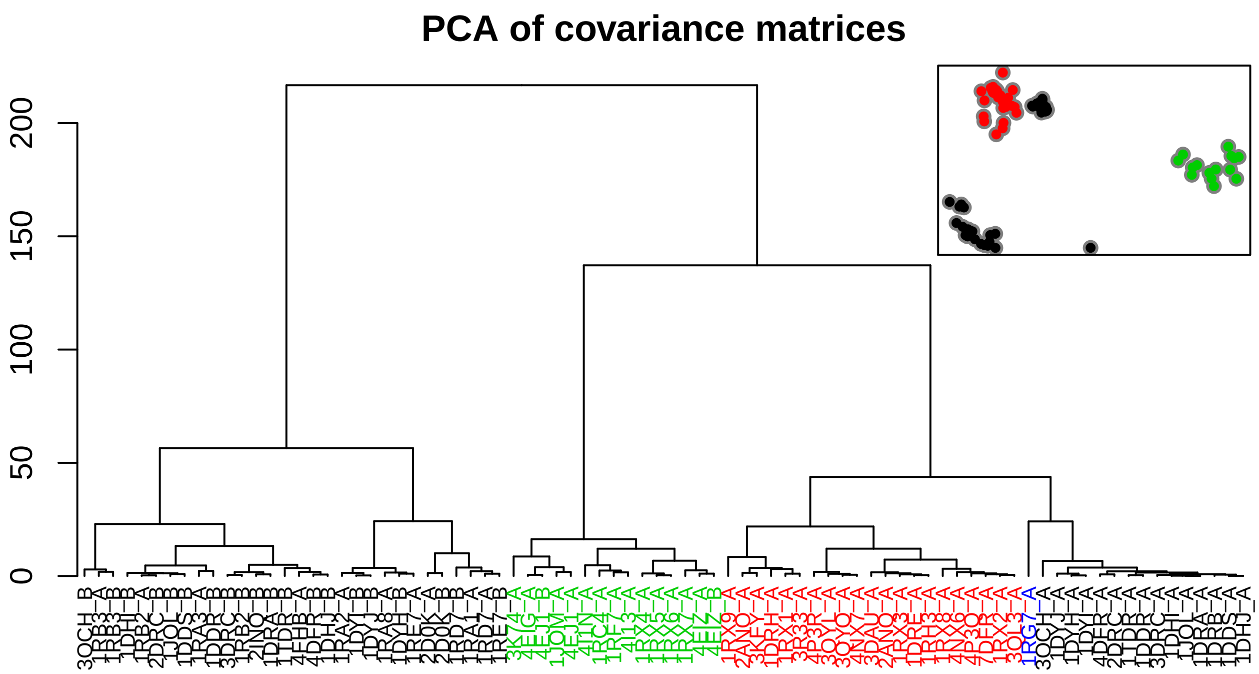


Figure 15: Dendrogram shows the results of hierarchical clustering of structures based on the PCA of covariance matrices (calculated from NMA). Colors of the labels depict associated conformatial state: green (occluded), black (open), and red (closed). The inset shows the conformerplot (see Figure 2), with colors according to clustering based on PCA of covariance matrices.

```r
print(sessionInfo(), FALSE)
```

```
## R version 3.1.1 (2014-07-10)
## Platform: x86_64-redhat-linux-gnu (64-bit)
##
## attached base packages:
## [1] stats     graphics  grDevices utils     datasets  methods   base
##
## other attached packages:
## [1] ncdf_1.6.6      bio3d_2.1-0     rmarkdown_0.3.3
##
## loaded via a namespace (and not attached):
## [1] digest_0.6.4       evaluate_0.5.5     formatR_0.10
## [4] htmltools_0.2.6    KernSmooth_2.23-12 knitr_1.6
## [7] stringr_0.6.2      tools_3.1.1        yaml_2.1.13
```

## References

Grant, B.J., A.P.D.C Rodrigues, K.M. Elsawy, A.J. Mccammon, and L.S.D. Caves. 2006. "Bio3d: An R Package for the Comparative Analysis of Protein Structures." *Bioinformatics* 22: 2695–96. doi:10.1093/bioinformatics/btl461.

Romanowska, Julia, Krzysztof S. Nowinski, and Joanna Trylska. 2012. "Determining geometrically stable domains in molecular conformation sets." *Journal of Chemical Theory and Computation* 8 (8): 2588–99. doi:10.1021/ct300206j.
