# Supplementary material for: Integrating protein structural dynamics and evolutionary analysis with Bio3D
Source: BMC Bioinformatics. 2014 Dec 10;15(1):399. doi: 10.1186/s12859-014-0399-6 (PMC4279791; doi:10.1186/s12859-014-0399-6)
Supplement: Additional file 3: — Species wide NMA of the DHFR superfamily. [file 12859_2014_399_MOESM3_ESM.pdf]

Ensemble NMA across multiple species of DHFR

Lars Skjaerven, Xin-Qiu Yao, Guido Scarabelli & Barry J. Grant

October 1, 2014

This document provides **Additional File 3** for *Integrating protein structural dynamics and evolutionary analysis with Bio3D.*

Background

Bio3D[1] is an R package that provides interactive tools for structural bioinformatics. The primary focus of Bio3D is the analysis of bimolecular structure, sequence and simulation data (Grant et al. 2006).

Normal mode analysis (NMA) is one of the major simulation techniques used to probe large-scale motions in biomolecules. Typical application is for the prediction of functional motions in proteins. Version 2.0 of the Bio3D package now includes extensive NMA facilities (see also the **NMA Vignette**). These include a unique collection of multiple elastic network model force-fields, automated ensemble analysis methods, and variance weighted NMA. Here we provide an in-depth demonstration of ensemble NMA with working code that comprise complete executable examples[2].

Requirements

Detailed instructions for obtaining and installing the Bio3D package on various platforms can be found in the **Installing Bio3D Vignette** available both on-line and from within the Bio3D package. In addition to Bio3D the *MUSCLE* and *CLUSTALO* multiple sequence alignment programs (available from the muscle home page and clustalo home page) must be installed on your system and in the search path for executables. Please see the installation vignette for further details.

About this document

This vignette was generated using **Bio3D version 2.1.0**.

1 Part II: Ensemble NMA across multiple species of DHFR

In this vignette we extend the analysis from Part I by including a more extensive search of distant homologues within the DHFR family. Based on a HMMER search we identify and collect protein species down to a pairwise sequence identity of 21%. Normal modes analysis (NMA) across these species reveals a remarkable similarity of the fluctuation profiles, but also features which are characteristic to specific species.

[1]The latest version of the package, full documentation and further vignettes (including detailed installation instructions) can be obtained from the main Bio3D website: http://thegrantlab.org/bio3d/

[2]This vignette contains executable examples, see `help(vignette)` for further details.

1.1 HMMER search for distantly related DHFR species

Below we use the sequence of *E.coli* DHFR to perform an initial search against the Pfam HMM database with function **hmmer()**. The arguments `type` and `db` specifies the type of hmmer search and the database to search, respectively. In this particular example, our query sequence is searched against the Pfam profile HMM library (arguments `type=hmmscan` and `db=pfam`) to identify its respecitve protein family. The to **hmmer()** will return a data frame object containing the Pfam accession ID (`$acc`), description of the identified family (`$desc`), family name (`$name`), etc.

```
# load the bio3d package
library(bio3d)

# get sequence of Ecoli DHFR
seq <- get.seq("1rx2_A")

# scan the Pfam database for our sequence
pfam <- hmmer(seq, type="hmmscan", db="pfam")
```

```
## Loading required package: XML
## Loading required package: RCurl
## Loading required package: bitops
```

```
pfam$name
pfam$desc
```

Sidenote: The **hmmer()** function facilitates four different types of searches at a multitude of databases. Use function `help(hmmer)` for a complete overview of the different options.

Having identified the Pfam entry of our query protein we can use function **pfam()** to fetch the curated sequence alignment of the DHFR family. Use function **print.fasta()** to print a short summary of the downloaded sequence alignment to the screen. Note that if argument `alignment=TRUE` the sequence alignmenment itself will be written to screen.

```
# download pfam alignment for the DHFR family
pfam.aln <- pfam(pfam$acc[1])
print(pfam.aln, alignment=FALSE)
```

```
##
## Call:
##   pfam(id = pfam$acc[1])
##
## Class:
##   fasta
##
## Alignment dimensions:
##   89 sequence rows; 214 position columns (110 non-gap, 104 gap)
##
## + attr: id, ali, call
```

The next hmmer search builds a profile HMM from the Pfam multiple sequence alignment and uses this HMM to search against a target sequence database (use `type=hmmsearch`). In this case our target sequence database is the PDB (`db=pdb`), but there are also other options such as *Swissprot* and *UniProt*.

```
# use Pfam alignment in search
hmm <- hmmer(pfam.aln, type="hmmsearch", db="pdb")
```

Function **plot.hmmer()** (the equivalent to **plot.blast()**) provides a quick overview of the rearch results, and can aid in the identification of a sensible hit similarity threshold. The normalized scores (-log(E-Value)) are shown in the upper panel, and the lower panel provides an overview of the kingdom and specie each hit are associated with. Here we specify a cutoff of 90 yielding 517 hits:

```
hits <- plot.hmmer(hmm, cutoff=90)
```

```
##   * Possible cutoff values:    97 13
##             Yielding Nhits:    517 560
##
##   * Chosen cutoff value of:    90
##             Yielding Nhits:    517
```

```
ids <- hits$acc
species <- hmm$species[hits$inds]
```

```
# collected species
print(unique(species))
```

```
##  [1] "Bacillus anthracis"
##  [2] "Bacillus anthracis str. Sterne"
##  [3] "Geobacillus stearothermophilus"
##  [4] "Coxiella burnetii"
##  [5] "Moritella profunda"
##  [6] "Yersinia pestis CO92"
##  [7] "Escherichia coli B"
##  [8] "Escherichia coli O6:H1 (strain CFT073 / ATCC 700928 / UPEC)"
##  [9] "Escherichia coli K-12"
## [10] "Escherichia coli UTI89"
## [11] "Enterococcus faecalis V583"
## [12] "Staphylococcus aureus"
## [13] "Staphylococcus aureus RF122"
## [14] "Streptococcus pneumoniae"
## [15] "Lactobacillus casei"
## [16] "Mycobacterium tuberculosis (strain ATCC 25618 / H37Rv)"
## [17] "Mycobacterium avium"
## [18] "Haloferax volcanii"
```

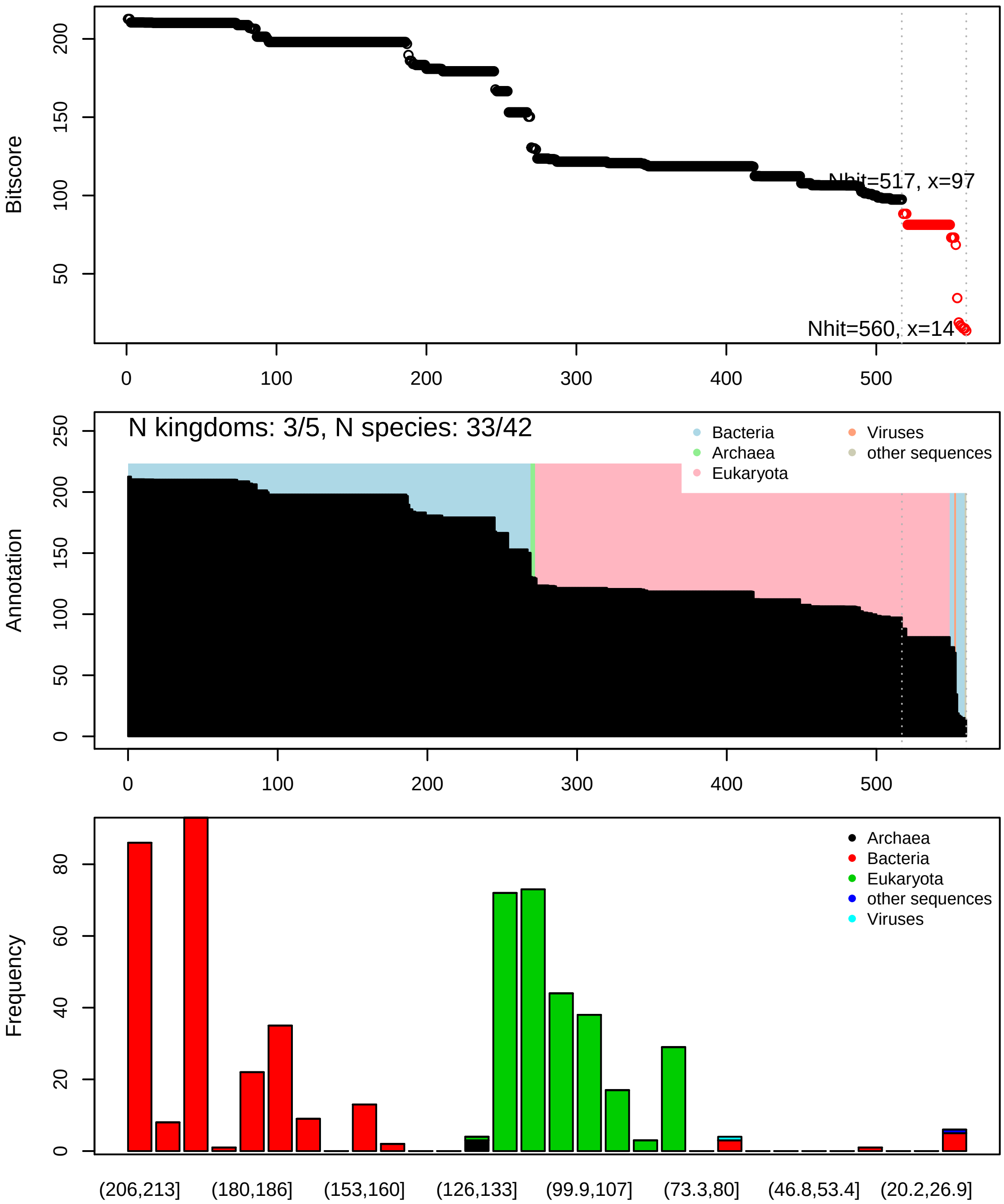

Figure 1: Overview of hits obtained from the HMMER search. Upper panel shows the normalized scores. Lower panel the scores and hits are colored according to their respective kingdom (background colors) and specie (foreground barplot).

```
## [19] "Haloferax volcanii (strain ATCC 29605 / DSM 3757 / JCM 8879 / NBRC 14742 / NCIMB 2012
## [20] "Schistosoma mansoni"
## [21] "Gallus gallus"
## [22] "Mus musculus"
## [23] "Cryptosporidium hominis"
## [24] "Pneumocystis carinii"
## [25] "Homo sapiens"
## [26] "Candida albicans"
## [27] "Babesia bovis"
## [28] "Candida glabrata CBS 138"
## [29] "Candida glabrata"
## [30] "Toxoplasma gondii"
## [31] "Plasmodium vivax"
## [32] "Plasmodium falciparum"
## [33] "Plasmodium falciparum VS/1"
```

1.2 Retrieve and process structures from the PDB

Having identified relevant PDB structures through the hmmer search we proceed by fetching and pre-processing the PDB files with functions **get.pdb()** and **pdbsplit()**.

As in the previous vignette, we are interested in protein structures without missing in-structure residues, and we also want to limit the number of identifical conformers:

```
# fetch and split PDBs
raw.files <- get.pdb(ids, path = "raw_pdbs", gzip=TRUE)
files <- pdbsplit(raw.files, ids = ids,
                  path = "raw_pdbs/split_chain", ncore=4)
```

```
## Loading required package: parallel
```

```
pdbs.all <- pdbaln(files)

# exclude DHFR-TS fusion protein
excl.inds <- unlist(lapply(c("1qzf", "1sej"), grep, pdbs.all$id))
pdbs <- pdbs.filter(pdbs.all, row.inds=-excl.inds)

# exclude structures with missing residues
conn <- inspect.connectivity(pdbs, cut=4.05)
pdbs <- pdbs.filter(pdbs, row.inds=which(conn))

# exclude conformational redundant structures
rd <- rmsd.filter(pdbs$xyz, cutoff=0.25, fit=TRUE, ncore=4)
pdbs <- pdbs.filter(pdbs, row.inds=rd$ind)
```

In this particular case a standard sequence alignment (e.g. through function **pdbaln()** or **seqaln()**) is not sufficient for a correct alignment. We will therefore make use of the Pfam profile alignment,

and align our selected PDBs to this using argument `profile` to function **seqaln()**. Subsequently, we re-read the fasta file, and use function **read.fasta.pdb()** to obtain aligned C-alpha atom data (including coordinates etc.) for the PDB ensemble:

```
# align pdbs to Pfam-profile alignment
aln <- seqaln(pdbs, profile=pfam.aln, exefile="clustalo", extra.args="--dealign")

# store only PDBs in alignment
aln$ali=aln$ali[1:length(pdbs$id),]
aln$id=aln$id[1:length(pdbs$id)]

# re-read PDBs to match the new alignment
pdbs <- read.fasta.pdb(aln)

# exclude gap-only columns
pdbs = pdbs.filter(pdbs)

# refit coordinates
pdbs$xyz = pdbfit(pdbs)
```

```
# refit coordinates, and write PDBs to disk
pdbs$xyz <- pdbfit(pdbs, outpath="flsq/")
```

```
# fetch IDs again
ids = unlist(strsplit(basename(pdbs$id), split="\\.pdb"))
species = hmm$species[hmm$acc %in% ids]
```

```
# labels for annotating plots
labs <- paste(substr(species, 1,1), ". ",
                 lapply(strsplit(species, " "), function(x) x[2]), sep="")
print(unique(labs))
```

```
##  [1] "B. anthracis"          "G. stearothermophilus"
##  [3] "M. profunda"           "Y. pestis"
##  [5] "E. coli"               "E. faecalis"
##  [7] "S. aureus"             "S. pneumoniae"
##  [9] "L. casei"              "M. tuberculosis"
## [11] "M. avium"              "H. volcanii"
## [13] "S. mansoni"            "G. gallus"
## [15] "M. musculus"           "P. carinii"
## [17] "H. sapiens"            "C. albicans"
## [19] "C. glabrata"
```

The *pdbs* object now contains *aligned* C-alpha atom data, including Cartesian coordinates, residue numbers, residue types, and B-factors. The sequence alignment is also stored by default to the FASTA format file 'aln.fa' (to view this you can use an alignment viewer such as SEAVIEW, see *Requirements* section above).

1.3 Sequence conservation analysis

Function **seqidentity()** can be used to calculate the sequence identity for the PDBs ensemble. Below we also print a summary of the calculated sequence identities, and perform a clustering of the structures based on sequence identity:

```
seqide <- seqidentity(pdbs)
summary(c(seqide))
```

```
##    Min. 1st Qu.  Median    Mean 3rd Qu.    Max.
##   0.216   0.309   0.333   0.434   0.391   1.000
```

```
hc <- hclust(as.dist(1-seqide))
grps.seq <- cutree(hc, h=0.6)
```

```
hclustplot(hc, k=3, labels=labs, cex=0.25, fillbox=FALSE)
```

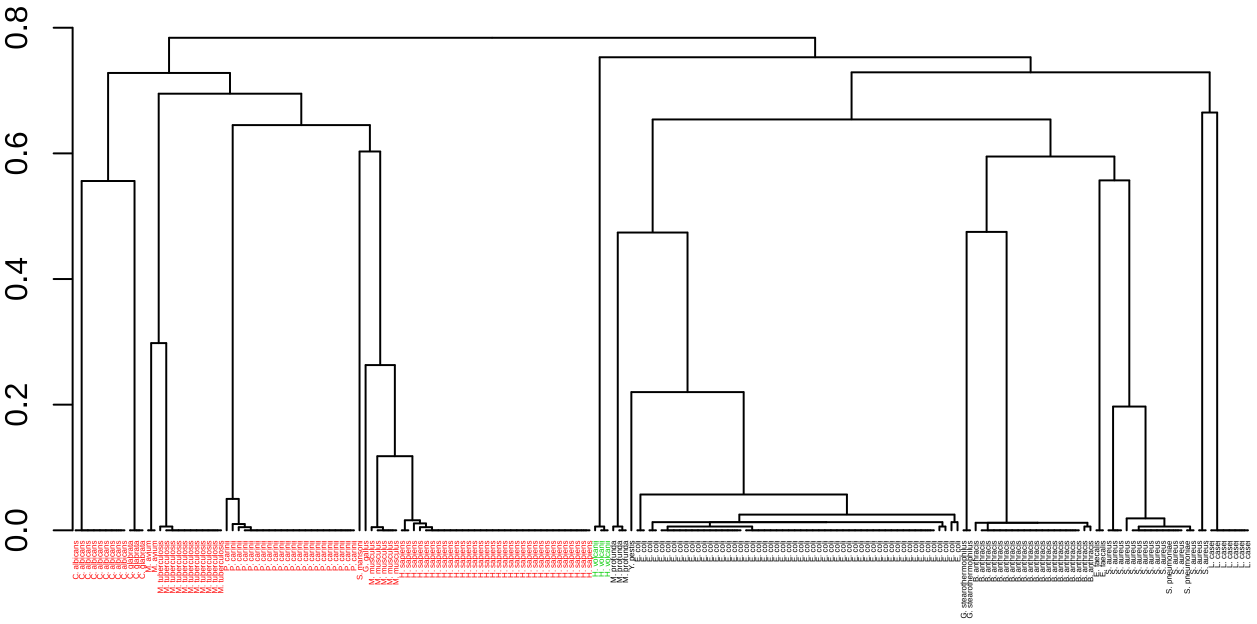

Figure 2: Clustering of collected structures based on sequence identity.

1.4 Normal modes analysis

Function **nma.pdbs()** will calculate the normal modes of each protein structures stored in the *pdbs* object. The normal modes are calculated on the full structures as provided by object *pdbs*. Use argument `rm.gaps=FALSE` to visualize fluctuations also of un-aligned residues:

```
modes <- nma.pdbs(pdbs, rm.gaps=FALSE, ncore=4)
```

```
## Loading required package: bigmemory
## Loading required package: bigmemory.sri
## Loading required package: BH
##
## bigmemory >= 4.0 is a major revision since 3.1.2; please see packages
## biganalytics and and bigtabulate and http://www.bigmemory.org for more information.
```

The *modes* object of class *enma* contains aligned normal mode data including fluctuations, RMSIP data (only when`rm.gaps=FALSE`), and aligned eigenvectors. A short summary of the *modes* object can be obtain by calling the function **print()**, and the aligned fluctuations can be plotted with function **plot.enma()**:

```
print(modes)
```

```
##
## Call:
##   nma.pdbs(pdbs = pdbs, rm.gaps = FALSE, ncore = 4)
##
## Class:
##   enma
##
## Number of structures:
##   195
##
## Attributes stored:
##   - Aligned atomic fluctuations
##   - Aligned eigenvectors (gaps not removed)
##   - Dimensions of x$U.subspace: 723x378x195
##
## Coordinates were aligned prior to NMA calculations
##
## + attr: fluctuations, rmsip, U.subspace, L, full.nma, xyz,
##         call
```

```
plot(modes, pdbs=pdbs, ylim=c(0,2), col=grps.seq, conservation=TRUE)
```

In some cases it can be difficult to interpret the fluctuation plot when all lines are plotted on top of each other. Argument `spread=TRUE` adds a small gap between grouped fluctuation profiles. Use this argument in combination with a new groups (`grps`) variable to function **plot.enma()**:

```
grps <- rep(NA, length(grps.seq))
grps[grep("coli", labs)]=1
grps[grep("aureus", labs)]=2
```

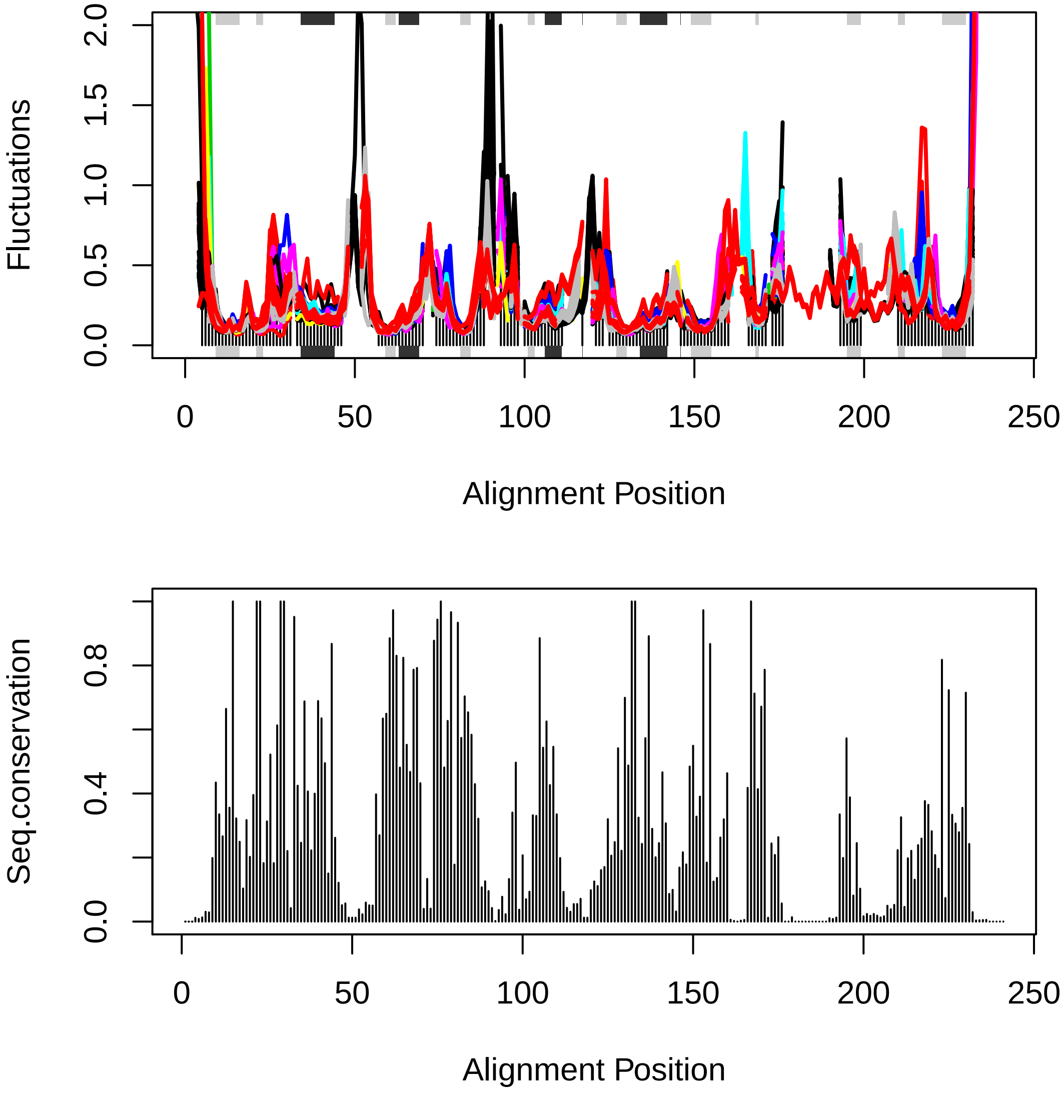

Figure 3: Flexibility profiles and sequence conservation. The figure shows the modes fluctuations colored according their sequence identity. The lower panel shows the sequence conservation for the PDBs. The plot is generated with function **plot.enma()** with argument `conservation=TRUE`.

```
grps[grep("anthracis", labs)]=3
grps[grep("tubercu", labs)]=4
grps[grep("casei", labs)]=5
grps[grep("sapiens", labs)]=6
grps[grep("albicans", labs)]=7
grps[grep("glabrata", labs)]=8

plot(modes, pdbs=pdbs, col=grps, spread=TRUE)
```

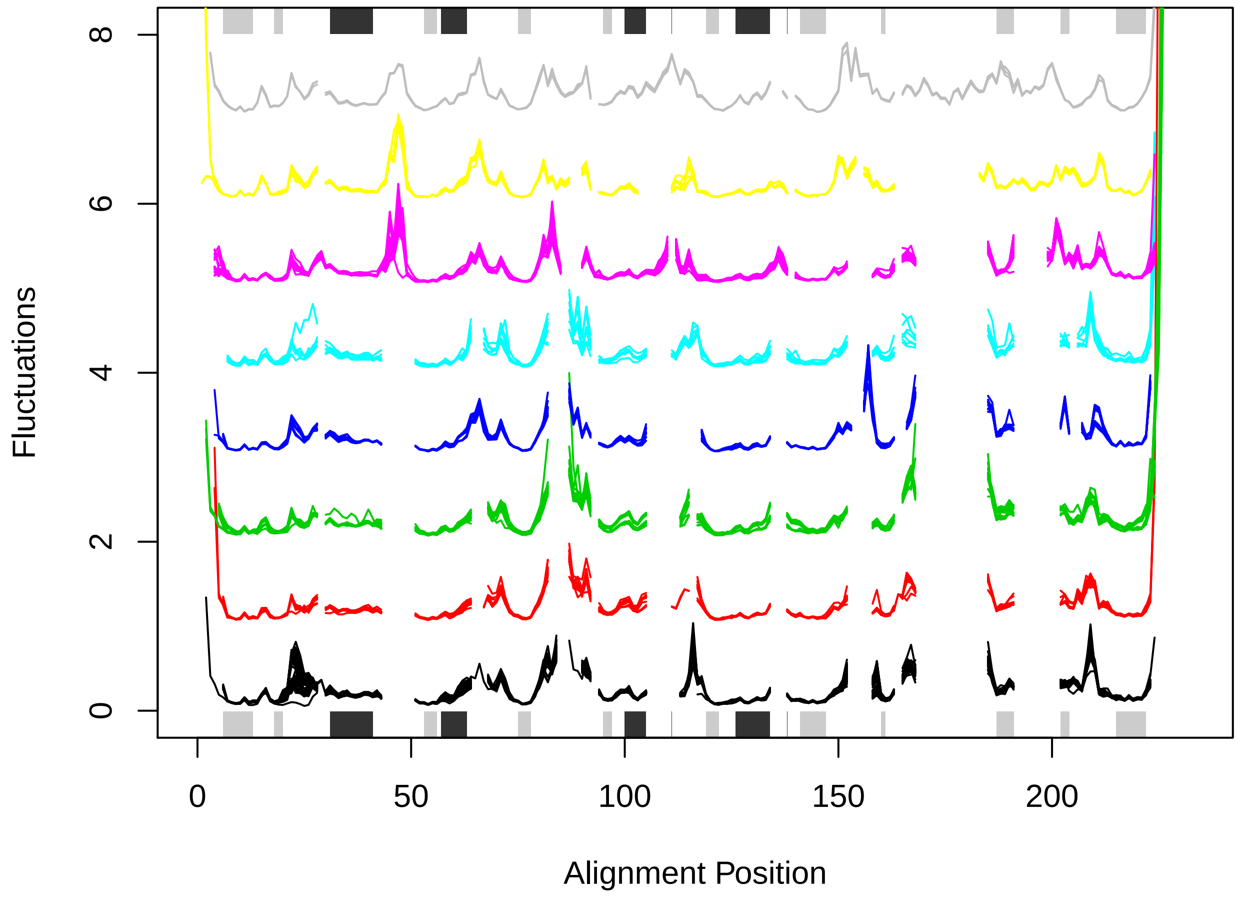

Figure 4: Flexibility profiles for three selected species (*E.coli* (black), *H.sapiens* (red), and *C.albicans* (green)). The plot is generated with function **plot.enma()** with argument `spread=TRUE`.

1.5 Visualize modes

A function call to **mktrj.enma()** will generate a trajectory PDB file for the visualization of a specific normal mode for one of the structures in the *pdbs* object. This allows for a visual comparison of the calculated normal modes. Below we make a PDB trajectory of the first mode (argument `m.inds=1`) of 3 relevant species (e.g. argument `s.inds=1`). Note that we use **grep()** to fetch the indices (in the *modes* and *pdbs* objects) of the relevant species:

```r
inds <- c(grep("coli", species)[1],
          grep("sapiens", species)[1],
          grep("albicans", species)[1])

# E.coli
mktrj.enma(modes, pdbs, m.inds=1, s.inds=inds[1], file="ecoli-mode1.pdb")

# H. sapiens
mktrj.enma(modes, pdbs, m.inds=1, s.inds=inds[2], file="hsapiens-mode1.pdb")

# C. albicans
mktrj.enma(modes, pdbs, m.inds=1, s.inds=inds[3], file="calbicans-mode1.pdb")
```

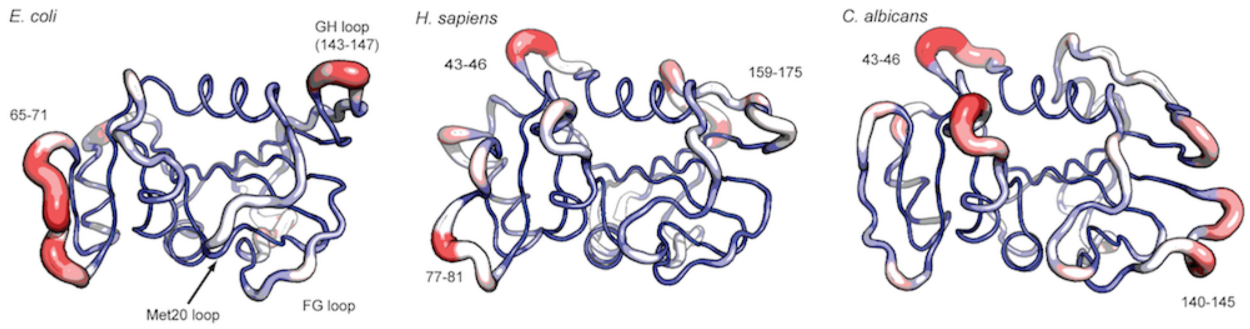

Figure 5: Mode comparison of *E.coli*, *H.sapiens*, and *C.albicans*. The trajectories are made with function **mktrj.enma()** and visualized in PyMol.

Document Details

This document is shipped with the Bio3D package in both R and PDF formats. All code can be extracted and automatically executed to generate Figures and/or the PDF with the following commands:

```r
library(rmarkdown)
render("Bio3D_nma-dhfr-partII.Rmd", "all")
```

Information About the Current Bio3D Session

```r
print(sessionInfo(), FALSE)
```

```
## R version 3.1.1 (2014-07-10)
## Platform: x86_64-redhat-linux-gnu (64-bit)
##
## attached base packages:
## [1] stats     graphics  grDevices utils     datasets  methods   base
##
## other attached packages:
## [1] bio3d_2.1-0     rmarkdown_0.3.3
##
## loaded via a namespace (and not attached):
## [1] digest_0.6.4    evaluate_0.5.5  formatR_0.10    htmltools_0.2.6
## [5] knitr_1.6       stringr_0.6.2   tools_3.1.1     yaml_2.1.13
```

References

Grant, B.J., A.P.D.C Rodrigues, K.M. Elsawy, A.J. Mccammon, and L.S.D. Caves. 2006. "Bio3d: An R Package for the Comparative Analysis of Protein Structures." *Bioinformatics* 22: 2695–96. doi:10.1093/bioinformatics/btl461.
