# Supplementary material for: Integrating protein structural dynamics and evolutionary analysis with Bio3D
Source: BMC Bioinformatics. 2014 Dec 10;15(1):399. doi: 10.1186/s12859-014-0399-6 (PMC4279791; doi:10.1186/s12859-014-0399-6)
Supplement: Additional file 4: — Complete example of the integration of ensemble NMA with correlation network analysis. [file 12859_2014_399_MOESM4_ESM.pdf]

Integrating normal modes and correlation network analysis in Bio3D

Lars Skjaerven, Xin-Qiu Yao, Guido Scarabelli & Barry J. Grant

October 1, 2014

This document provides **Additional File 4** for *Integrating protein structural dynamics and evolutionary analysis with Bio3D*.

Background

Bio3D[1] is an R package that provides interactive tools for structural bioinformatics. The primary focus of Bio3D is the analysis of bimolecular structure, sequence and simulation data (Grant et al. 2006).

Correlation network analysis can be employed to identify protein segments with correlated motions. In this approach, a weighted graph is constructed where each residue represents a node and the weight of the connection between nodes, i and j, represents their respective cross-correlation value, cij, expressed for example by the Pearson-like form or the linear mutual information. In this example, Normal mode analysis (NMA) is employed for the calculation of cross-correlations[2].

Requirements

Detailed instructions for obtaining and installing the Bio3D package on various platforms can be found in the **Installing Bio3D Vignette** available both on-line and from within the Bio3D package. Note that this vignette also makes use of the IGRAPH R package, which can be installed with the command

```
install.packages("igraph")
```

About this document

This vignette was generated using **Bio3D version 2.1.0**.

1 Correlation network analysis based on single-structure NMA

In this first example we perform *NMA* on two crystallographic structures of transducin G protein alpha subunits, an active GTP-analog-bound structure (PDB id *1tnd*) and an inactive GDP- and GDI (GDP dissociation inhibitor)-bound structure (PDB id *1kjy*), by calling the function **nma()**.

[1]The latest version of the package, full documentation and further vignettes (including detailed installation instructions) can be obtained from the main Bio3D website: http://thegrantlab.org/bio3d/.

[2]This vignette contains executable examples, see `help(vignette)` for further details.

Cross-correlation matrices are calculated with the function **dccm()**. Correlation networks are then constructed with the function **cna()**. Network visualization is finally performed with the function **plot.cna()**. See also `help(cna)` for more details and example analysis.

```
library(bio3d)
```

First we read our selected GTP and GDI bound structures, select chain A, and perform NMA on each individually.

```
pdb.gtp = read.pdb("1TND")
pdb.gdi = read.pdb("1KJY")

pdb.gtp = trim.pdb(pdb.gtp, inds=atom.select(pdb.gtp, chain="A"))
pdb.gdi = trim.pdb(pdb.gdi, inds=atom.select(pdb.gdi, chain="A"))
```

```
modes.gtp = nma(pdb.gtp)
```

```
##  Building Hessian...     Done in 0.189 seconds.
##  Diagonalizing Hessian...    Done in 1.99 seconds.
```

```
modes.gdi = nma(pdb.gdi)
```

```
##  Building Hessian...     Done in 0.194 seconds.
##  Diagonalizing Hessian...    Done in 1.883 seconds.
```

Residue cross-correlations can then be calculated based on these normal mode results.

```
cij.gtp = dccm(modes.gtp)
cij.gdi = dccm(modes.gdi)
```

Correlation networks for both conformational states are constructed by applying the function **cna()** to the corresponding correlation matrices. Here, the C-alpha atoms represents nodes which are interconnected by edges with weights corresponding to the pairwise correlation coefficient. Edges are only constructed for pairs of nodes which obtain a coupling strength larger than a specified cutoff value (0.35 in this example). The weight of each edge is calculated as -log(|cij|), where cij is the correlation value between two nodes *i* and *j*. For each correlation network, betweenness clustering is performed to generate aggregate nodal clusters, or communities, that are highly intra-connected, but loosely inter-connected. By default, **cna()** returns communities associated with the maximal modularity value.

```
net.gtp = cna(cij.gtp, cutoff.cij=0.35)
net.gdi = cna(cij.gdi, cutoff.cij=0.35)
```

```r
net.gtp
```

```
##
## Call:
##   cna.dccm(cij = cij.gtp, cutoff.cij = 0.35)
##
## Structure:
##  - NETWORK NODES#:   323     EDGES#: 1427
##  - COMMUNITY NODES#: 12  EDGES#: 22
##
##  + attr: network, communities, community.network,
##         community.cij, cij, call
```

```r
net.gdi
```

```
##
## Call:
##   cna.dccm(cij = cij.gdi, cutoff.cij = 0.35)
##
## Structure:
##  - NETWORK NODES#:   320     EDGES#: 1587
##  - COMMUNITY NODES#: 8   EDGES#: 11
##
##  + attr: network, communities, community.network,
##         community.cij, cij, call
```

A 3-D visualization of networks can also be performed with the Bio3D function **view.dccm()** (See `help(view.dccm)` and the vignette "Enhanced Methods for Normal Mode Analysis with Bio3D" available on-line):

```r
view.dccm(net.gtp$cij, launch=TRUE)
```

Maximization of modularity sometimes creates unexpected community partitions splitting natural structure motifs, such as secondary structure elements, into many small community 'islands'. To avoid this situation, we look into partitions with modularity close to the maximal value but with an overall smaller number of communities.

```r
mod.select <- function(x, thres=0.005) {
   remodel <- community.tree(x, rescale = TRUE)
   n.max = length(unique(x$communities$membership))
   ind.max = which(remodel$num.of.comms == n.max)
   v = remodel$modularity[length(remodel$modularity):ind.max]
   v = rev(diff(v))
   fa = which(v>=thres)[1] - 1
   ncomm = ifelse(is.na(fa), min(remodel$num.of.comms), n.max - fa)
```

```
  ind <- which(remodel$num.of.comms == ncomm)
  network.amendment(x, remodel$tree[ind, ])
}
```

```
nnet.gtp = mod.select(net.gtp)
nnet.gtp
```

```
##
## Call:
##   cna.dccm(cij = cij.gtp, cutoff.cij = 0.35)
##
## Structure:
##  - NETWORK NODES#:   323     EDGES#: 1427
##  - COMMUNITY NODES#: 10  EDGES#: 18
##
##  + attr: network, communities, community.network,
##         community.cij, cij, call
```

```
nnet.gdi = mod.select(net.gdi)
nnet.gdi
```

```
##
## Call:
##   cna.dccm(cij = cij.gdi, cutoff.cij = 0.35)
##
## Structure:
##  - NETWORK NODES#:   320     EDGES#: 1587
##  - COMMUNITY NODES#: 7   EDGES#: 9
##
##  + attr: network, communities, community.network,
##         community.cij, cij, call
```

The resulting networks can be visualized with the Bio3D function **plot.cna()**, which can generate 2D representations for both full residue-level and coarse-grained community-level networks.

```
cent.gtp.full = layout.cna(nnet.gtp, pdb=pdb.gtp, full=TRUE, k=3)[,1:2]
cent.gtp = layout.cna(nnet.gtp, pdb=pdb.gtp, k=3)[,1:2]
cent.gdi.full = layout.cna(nnet.gdi, pdb=pdb.gtp, full=TRUE, k=3)[,1:2]
cent.gdi = layout.cna(nnet.gdi, pdb=pdb.gtp, k=3)[,1:2]
```

The following code plots the four networks.

```
plot.cna(nnet.gtp, layout=cent.gtp.full, full=TRUE, vertex.label=NA, vertex.size=5)
plot.cna(nnet.gtp, layout=cent.gtp)
plot.cna(nnet.gdi, layout=cent.gdi.full, full=TRUE, vertex.label=NA, vertex.size=5)
plot.cna(nnet.gdi, layout=cent.gdi)
```

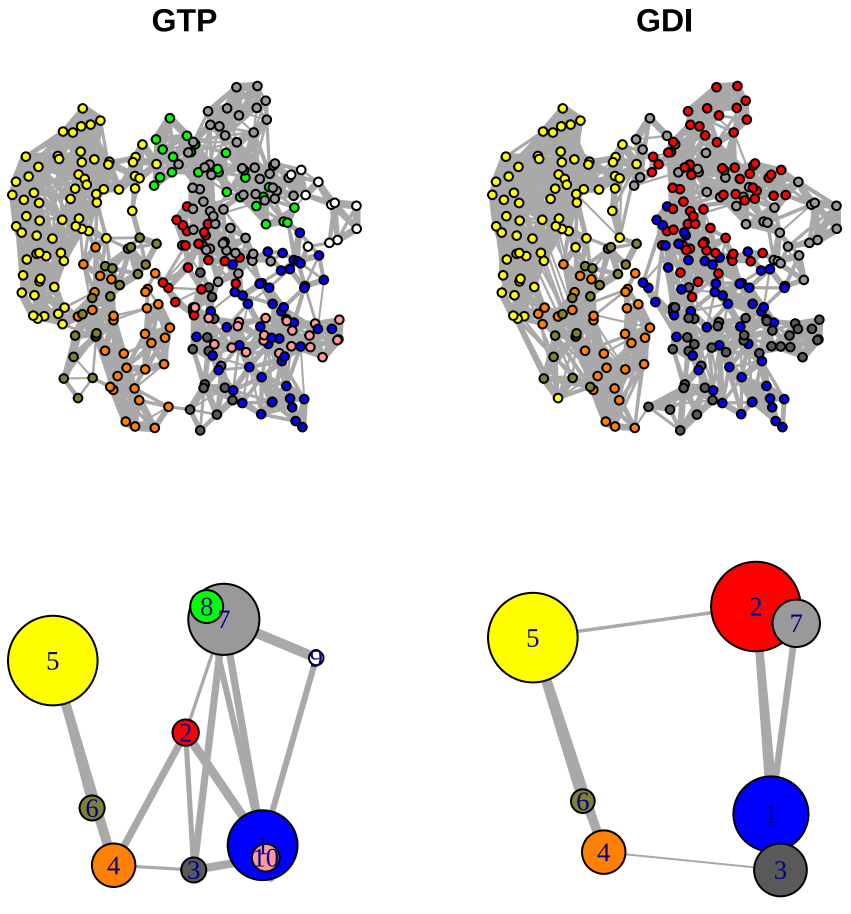

Figure 1: Correlation networks for GTP "active" and GDI "inhibitory" conformational states of transducin. Networks are derived from NMA applied to single GTP and GDI crystallographic structures.

2 Correlation network analysis based on ensemble NMA

In this example we perform ensemble NMA on 53 crystallographic transducin G-alpha structures, which can be categorized into active GTP-analog-bound, inactive GDP-bound, and inhibitory GDI-bound states. For this analysis we utilize the example transducin structure dataset (further details of which can be obtained via `help(transducin)` along with the "Comparative Protein Structure Analysis with Bio3D" vignette available on-line). Briefly, this dataset includes aligned PDB coordinates (`pdbs`), structural invariant core positions (`core`) and annotations for each PDB structure (`annotation`). Cross-correlation matrices for all structures in the ensemble are calculated with the function **dccm()**. State-specific ensemble average correlation matrices are then obtained with the function **filter.dccm()**. Correlation networks are finally constructed with the function **cna()**, and visualization of the networks performed with the function **plot.cna()**:

```
attach(transducin)
```

```
modes <- nma(pdbs, ncore=8)
```

```
cijs0 <- dccm(modes)
```

Below we perform optional filtering and averaging per state of the individual structure cross-correlation matrices. In this example, we also utilize a cutoff for correlation (0.35 in this example) and a cutoff for C-alpha distance (10 angstrom) (See `help(filter.dccm)` and main text for details).

```
cij <- filter.dccm(cijs0, xyz=pdbs, fac=annotation[, "state3"], cutoff.cij=0.35,
                dcut=10, scut=0, pcut=0.75, ncore=8)
```

```
nets <- cna(cij, cutoff.cij = 0)
net1 = mod.select(nets$GTP)
net2 = mod.select(nets$GDI)
```

```
ref.pdb <- pdbs2pdb(pdbs, inds=1, rm.gaps=TRUE)[[1]]
cent.gtp.full = layout.cna(net1, pdb=ref.pdb, full=TRUE, k=3)[,1:2]
cent.gtp = layout.cna(net1, pdb=ref.pdb, k=3)[,1:2]
cent.gdi.full = layout.cna(net2, pdb=ref.pdb, full=TRUE, k=3)[,1:2]
cent.gdi = layout.cna(net2, pdb=ref.pdb, k=3)[,1:2]
```

```
plot.cna(net1, layout=cent.gtp.full, full=TRUE, vertex.label=NA, vertex.size=5)
plot.cna(net1, layout=cent.gtp)
plot.cna(net2, layout=cent.gdi.full, full=TRUE, vertex.label=NA, vertex.size=5)
plot.cna(net2, layout=cent.gdi)
```

Document Details

This document is shipped with the Bio3D package in both R and PDF formats. All code can be extracted and automatically executed to generate Figures and/or the PDF with the following commands:

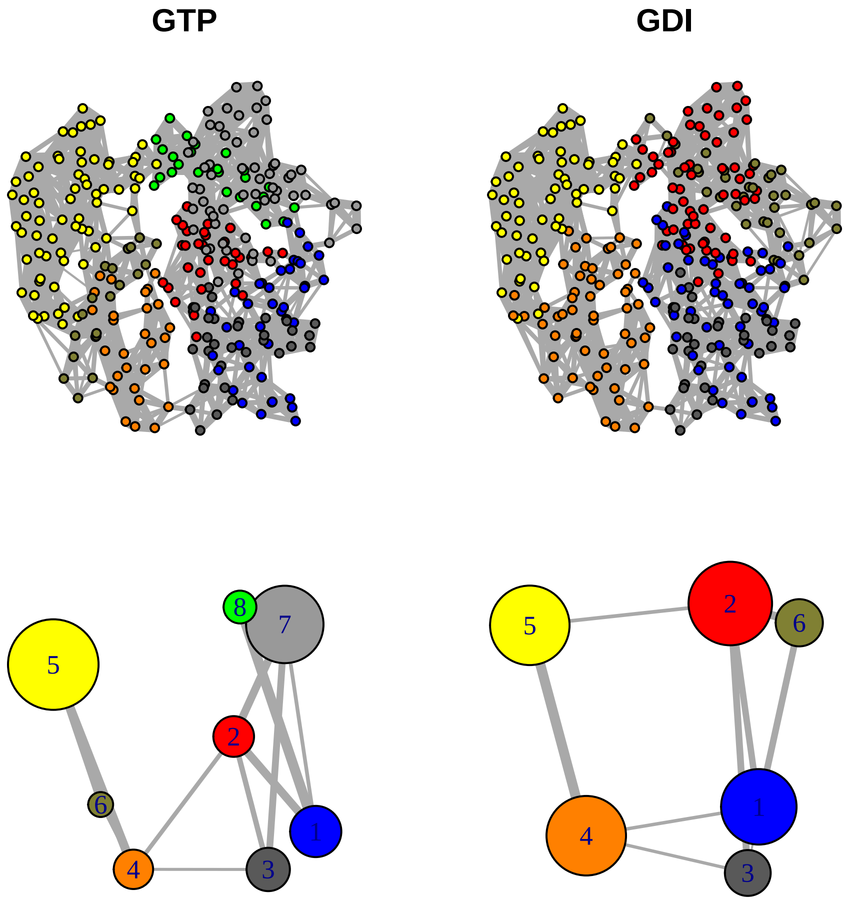

Figure 2: Correlation networks for GTP “active” and GDI “inhibitory” conformational states of transducin. Networks are derived from ensemble NMA of available GTP and GDI crystallographic structures.

```r
library(rmarkdown)
render("Bio3D_cna-transducin.Rmd", "all")
```

Information About the Current Bio3D Session

```r
sessionInfo()
```

```
## R version 3.1.1 (2014-07-10)
## Platform: x86_64-redhat-linux-gnu (64-bit)
##
## locale:
##  [1] LC_CTYPE=en_US.UTF-8       LC_NUMERIC=C
##  [3] LC_TIME=en_US.UTF-8        LC_COLLATE=en_US.UTF-8
##  [5] LC_MONETARY=en_US.UTF-8    LC_MESSAGES=en_US.UTF-8
##  [7] LC_PAPER=en_US.UTF-8       LC_NAME=C
##  [9] LC_ADDRESS=C               LC_TELEPHONE=C
## [11] LC_MEASUREMENT=en_US.UTF-8 LC_IDENTIFICATION=C
##
## attached base packages:
## [1] parallel  stats     graphics  grDevices utils     datasets  methods
## [8] base
##
## other attached packages:
## [1] bigmemory_4.4.6     BH_1.54.0-2         bigmemory.sri_0.1.2
## [4] igraph_0.7.1        rmarkdown_0.3.3     bio3d_2.1-0
##
## loaded via a namespace (and not attached):
## [1] codetools_0.2-8 digest_0.6.4    evaluate_0.5.5  formatR_0.10
## [5] htmltools_0.2.6 knitr_1.6       stringr_0.6.2   tools_3.1.1
## [9] yaml_2.1.13
```

References

Grant, B.J., A.P.D.C Rodrigues, K.M. Elsawy, A.J. Mccammon, and L.S.D. Caves. 2006. "Bio3d: An R Package for the Comparative Analysis of Protein Structures." *Bioinformatics* 22: 2695–96. doi:10.1093/bioinformatics/btl461.
